# Microwave-Accelerated Synthesis of Novel Triphosphate Nucleoside Prodrugs: Expanding the Therapeutic Arsenal of Anticancer Agents

**DOI:** 10.1021/acs.orglett.4c04379

**Published:** 2024-12-17

**Authors:** Camille Tisnerat, Samuele Di Ciano, Fabrizio Pertusati, Michaela Serpi

**Affiliations:** † School of Chemistry, Cardiff University, Main Building, Park Place, CF10 3AT Cardiff, Wales, United Kingdom; ‡ School of Pharmacy and Pharmaceutical Sciences, Redwood Building, King Edwards VII Avenue, CF10 3NB, Cardiff, Wales, United Kingdom

## Abstract

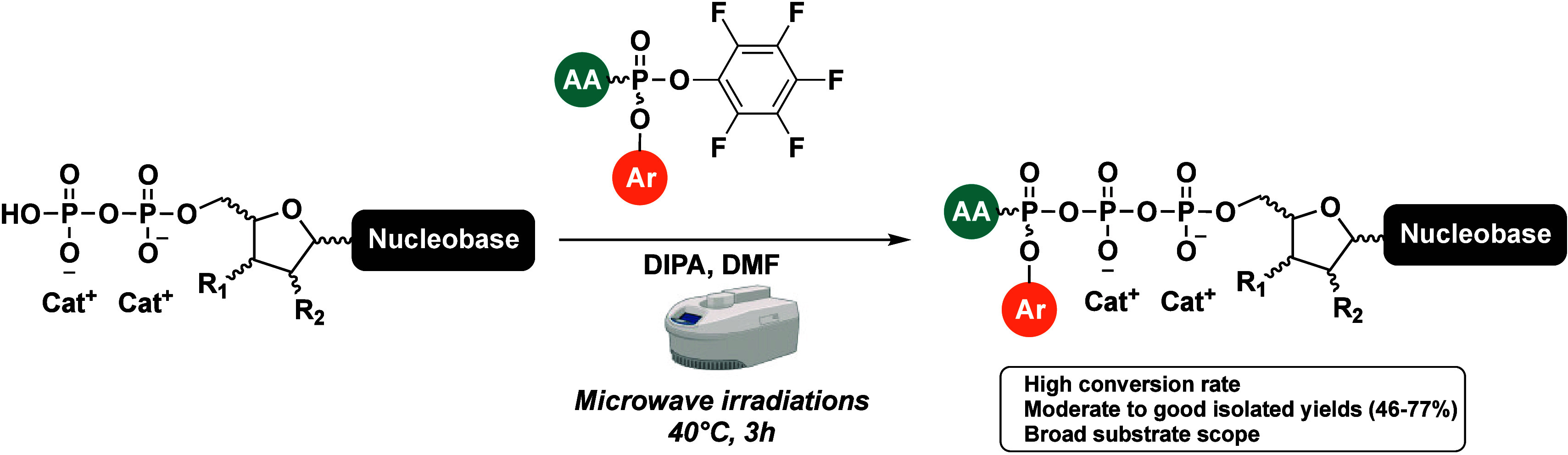

In this study, we report for the first time a microwave-accelerated synthesis of purine and pyrimidine nucleoside triphosphate prodrugs, whose γ phosphate is masked with an aryloxy moiety and an amino acid ester (γ-ProTriP). The synthetic utility of this method is illustrated by the synthesis of triphosphate prodrugs of clofarabine and gemcitabine, two FDA-approved anticancer drugs. These new prodrugs showed good chemical and rat serum stability. Remarkably the clofarabine prodrug showed significant cytotoxicity against a panel of cancer cell lines.

Nucleoside analogues (NAs) are currently used as effective drugs to treat various diseases such as viral infections and cancer conditions.^1^ They must undergo three *in vivo* phosphorylations in a stepwise manner to yield the corresponding active nucleoside triphosphate analogue, which exerts a therapeutic effect. Unfortunately, NAs suffer from many drawbacks such as poor cellular uptake because of insufficient expression of membrane transporters, premature breakdown, and slow conversion to triphosphate form due to rate-limiting phosphorylation steps.^1^ Within the kinase-catalyzed phosphorylation cascade, the first phosphorylation catalyzed by nucleoside kinases^2^ was often identified as the limiting step, and hence prodrugs of monophosphorylated NAs have been extensively studied.^3^ Among the most successful monophosphate prodrug approaches is the ProTide technology pioneered by Prof McGuigan in the late 1980.^4^ His studies have paved the way to the discovery and approval of the current marketed antiviral drugs Sofosbuvir (HCV),^5^ Tenofovir Alafenamide (HIV),^6^ and Remdesivir (COVID-19)^7^ but have also led to the development of several other clinical candidates in the anticancer area.^8^

However, less is known about the second and especially the third phosphorylation step catalyzed by the nucleoside monophosphate kinase (NMPK)^9^ and the nucleoside diphosphate kinase (NDPK), respectively.^10^ Some NAs have been reported to suffer from a second or third slow and inefficient phosphorylation step which is also associated with toxicity due to the accumulation of their mono- and diphosphate forms.^11^ Moreover, for many other synthesized NAs reported in the literature, the detailed metabolism to yield nucleoside triphosphate (NTP) is still not known.

Despite the fact that a triphosphate prodrug can clearly offer several unique advantages compared to a monophosphate prodrug, the design of higher phosphorylated NA prodrugs is relatively underexplored with only a few examples in the literature,^12^ of which the most recent are reported by Meier et al.^13^ A triphosphate prodrug can bypass the whole phosphorylation cascade and, at the same time, avoid potential metabolic hurdles and side effects caused by either deactivation or accumulation of the parent nucleoside or its di- or monophosphate forms.

As per our continuous efforts on the design and synthesis of novel prodrugs of NAs, we herein report for the first time an efficient microwave-accelerated synthesis of novel triphosphate nucleotide prodrugs that are masked at the γ phosphate with an aryloxy moiety and an amino acid ester (γ-ProTriP). Notably the strategy was successfully extended to clofarabine and gemcitabine, two FDA-approved anticancer drugs.

To identify the optimal conditions for the preparation of triphosphate prodrugs, adenosine diphosphate (ADP, **1**) and the pentafluorophenyl phosphorylating reagent **2a** bearing (*L*)-alanine benzyl ester and a phenyl group were selected as the model substrates (Table 1), and the reaction was investigated under thermal condition. The conversion of **1** into **3a** was monitored by ^31^P NMR, following both the disappearance of the two doublet peaks at δ = −10.8 and −11.3 ppm corresponding, respectively, to the α and β phosphorus of ADP (**1**) and the appearance of the three characteristic signals at δ = −7.1 (two doublet peaks), −12.5 (two doublet peaks), and −23.8 ppm (a multiplet peak) corresponding to the three phosphorus of the triphosphate prodrug **3a**, formed as a mixture of two diastereoisomers (ratio 1:1) due to the newly formed chiral center at the γ phosphorus atom (Figure S1). The integration of all these signals and the molar ratio between **1** and **3a** allowed calculation of the conversion percentage. Formation of diphosphate prodrug, arising from reaction of **2a** with adenosine monophosphate (formed by decomposition of ADP), was often observed but only in traces (<5%) if present. It was therefore not considered when calculating the conversion yield. Initially the reaction was carried out between substrate **1** (1 equiv) and a slight excess of **2a** (1.1 equiv)^5^ in the presence of an excess of Et_3_N (3 equiv) in DMF under inert atmosphere at room temperature for 16 h (Table 1, entry 1).

**Table 1:** Reaction Optimization^a^

Entry	2a (equiv)	Base (equiv)	Solvent	Heating	Temp (°C)	Time (h)	Convn (%)^d^	Yield (%)^e^
1	1.1	Et_3_N (3)	DMF	-	rt	16	61	46
2^b^	1.1	Et_3_N (3)	DMF	-	rt	16	0	
3	1.1	Et_3_N (3)	DME	-	rt	16	0	
4	1.2	Et_3_N (3)	CH_3_CN/Dioxane	-	rt	16	0	
5	1.1	DIPEA (3)	DMF	-	rt	16	63	
6	1.1	DBU (3)	DMF	-	rt	16	0	
7	1.1	NMI (3)	DMF	-	rt	16	10	
8	1.1	DMAP (3)	DMF	-	rt	16	0	
9	1.1	DIPA (3)	DMF	-	rt	16	86	
10	2	Et_3_N (3)	DMF	-	rt	16	92	
11	1.1	Et_3_N (3)	DMF	Standard	40	16	83	
12	1.1	DIPA (3)	DMF	Standard	40	16	94	
13	1.1	Et_3_N (3)	DMA	Standard	40	16	90	
14	1.1	Et_3_N (2)	DMF	Standard	40	16	92	57
15	1.1	DIPA (2)	DMF	Standard	40	16	94	67
16	1.1	DIPA (2)	DMF	MWI	40	6	88	
17	1.1	Et_3_N (2)	DMF	MWI	75	1	74	
18	1.1	Et_3_N (2)	DMF	MWI	65	1	67	
19	1.1	Et_3_N (2)	DMF	MWI	50	1	31	
20^c^	1.1	DIPA (2)	DMF	MWI	40	3	88	
						6	93	
21	2	DIPA (2)	DMF	MWI	40	3	91	
						6	96	67
22^c^	2	DIPA (2)	DMF	MWI	40	3	98	68

a Reaction conditions: ADP (**1**) (0.12 mmol) and **2a** were suspended in dry solvent (0.04M) before addition of the base. The mixture was stirred under N_2_, the solvent was evaporated, and the crude mixture was treated with 0.1 M triethylammonium bicarbonate buffer (TEAB) at pH 7.4. The progress of the reaction was monitored by ^13^P NMR (See Supporting Information for detailed protocols and spectra).

b ADP(Na)^2+^ as starting material.

c ADP (0.08M).

d Conversion yield determined by ^31^P NMR.

e Isolated yield.

The desired product **3a** was obtained in low yield (46%) after reverse-phase chromatography due to a modest conversion (61%). No reaction was observed when the disodium salt of ADP was used or when the DMF was replaced with dimethoxyethane (DME) or an acetonitrile/dioxane mixture, most probably due to the poor solubility of the starting nucleotides under these conditions (Table 1, entries 2–4). DMF was then deemed to be the best solvent for this reaction. We then began to screen the effects of organic bases. While diisopropylethylamine (DIPEA) (Table 1, entry 5) showed a similar conversion to Et_3_N, other bases such as 1,8-diazabicycloundecene DBU), *N*-methylimidazole (NMI), and 4-dimethylaminopyridine (DMAP) afforded either trace or no product (Table 1, entries 6–8). Only diisopropylamine (DIPA) showed a significant increase in conversion (86% vs 61%) (Table 1 entry 9 vs 1). Further improvements were achieved by doubling the equivalents of **2a**, which led to the excellent conversion yield of 92% (Table 1, entry 10) or by increasing the temperature up to 40 °C (under conventional heating), most probably due to an increased solubility of the starting nucleotide (Table 1, entries 11 and 12). With similar properties to DMF, DMA exhibited an excellent conversion of 90% (Table 1, entry 13) suggesting that the use of a highly aprotic polar solvent is optimal for the solubility of the starting material and the reaction. Interestingly, decreasing the equivalent of Et_3_N or DIPA did not impact the conversion (Table 1, entries 14–15) affording, after reverse-phase chromatography, compound **3a** as ditriethylammonium salt in 57% and 67% isolated yield, respectively. To shorten the reaction time and possibly improve the yield, we decided then to investigate the use of microwave irradiation (MWI), which had been previously reported for the synthesis of ProTides.^14^ Using our previous optimized conditions but under microwave irradiation, although we achieved a lower conversion (88% vs 94%), we were able to significantly decrease the reaction time (6 h compared to 16 h) without affecting selectivity (no phosphorylation of the 3′–OH is observed) (Table 1, entry 16 vs entry 15). Attempts to further increase the temperature were not compatible with the stability of ADP (**1**), leading to the formation of a significant amount of the diphosphate prodrug side product (Table 1, entries 17–19), previously seen only in traces. Successful yield enhancements were achieved by doubling the number of equivalents of **2a** or using a nucleotide concentration of 0.08 M (Table 1, entries 20–21). Using the optimal reaction conditions, the triethylammonium salt of triphosphate prodrug **3a** was obtained in 68% yield after reverse-phase column chromatography (Table 1, entry 22).

With the optimal conditions in hand, we evaluated the scope of the phosphorylating reagents (**2a**–**d**) bearing different amino acid ester moieties and aryloxy groups (Scheme 1). The different triphosphate prodrugs **3a**–**d** were isolated as a mixture of diastereoisomers (S_P_/R_P_ = 1:1) in good and consistent yields from 62% to 69%, except for **3d** that required a further purification, lowering the yield to 46%. The next step was to extend the scope of this methodology to pyrimidine nucleosides. Triethylammonium salt of uridine diphosphate (UDP, **4**) was used as a model substrate. It was prepared from UDP disodium salt by displacement on a cation exchange resin in the triethylammonium form.^15^ Again, the corresponding triphosphate prodrug **5a** was obtained as a mixture of diastereoisomers (S_P_/R_P_ = 1:1) in excellent isolated yield. Although phosphoramidate stereoisomers have the same chemical structure, it has been shown that R_p_ and S_p_ isomers can exhibit differences in their pharmacology, toxicology, and pharmacokinetics.^4,5,16^ With this in mind, we reacted the commercial phosphorylating reagent S_P_-**2b** with ADP (**1**) under the optimized reaction conditions to investigate whether it is possible to prepare pure S_P_ diastereoisomer of the triphosphate prodrug **3b**. To our surprise, **3b** was formed as a mixture of diastereoisomers S_P_/R_P_ = 2/1 as observed by ^31^P NMR, indicating that partial isomerization must occur at the phosphorus center of either S_P_-**2b** or S_P_-**3b** under the reaction condition. The same result was obtained using conventional heating at 40 °C or room temperature overnight. However, no isomerization occurred at either the phosphorus chiral center of S_P_-**2b** or at the prodrug **3b** (S_P_/R_P_ = 2:1) when they were independently irradiated by microwave at 40 °C in DMF in the presence of DIPA for 3 h. Reasoning that the acidic proton of the nucleoside diphosphate could play a role in the isomerization process, we repeated the same reaction using the ditriethylammonium salt of **1** and found that isomerization occurred to a much lesser extent leading to the formation of **3b** as a diastereoisomeric mixture S_P_/R_P_ = 9:1.

**Scheme 1:**
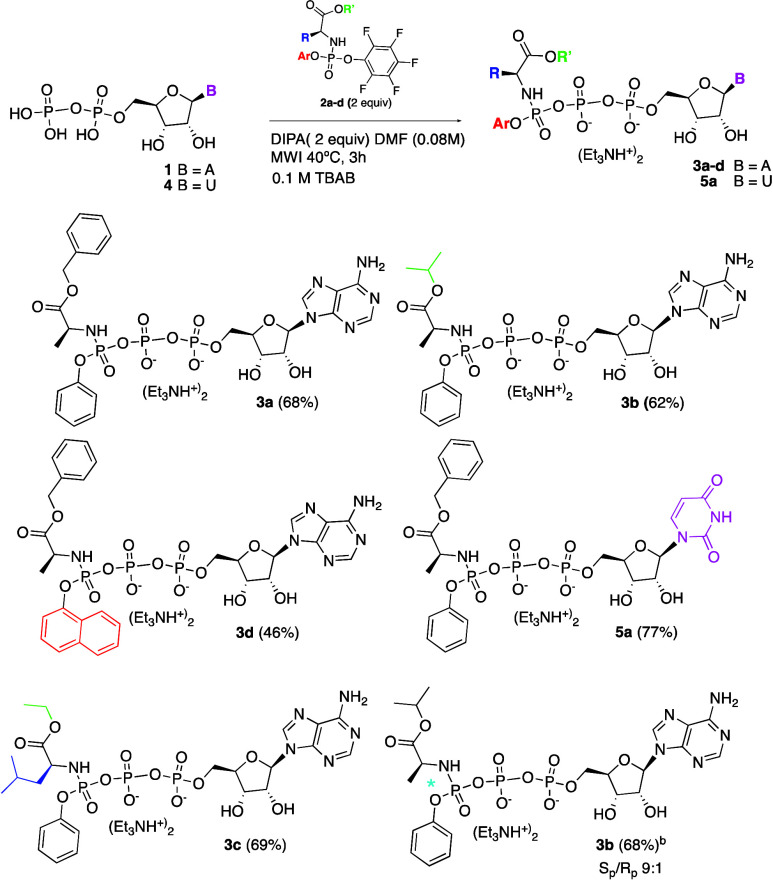
Substrate Scope: Variation of Nucleotide, Amino Acid Ester, and Aryloxy Moiety Reaction conditions: **1** or **4** (1 equiv) **2a**–**d** (2 equiv) were suspended in dry DMF (0.08M) before addition of DIPA (2 equiv). The mixture was stirred under N_2_ with microwave irradiation in a sealed vial at 40 °C for 3 h; the solvent was evaporated, and the crude mixture was treated with 0.1 M TEAB at pH 7.4. Pure diastereoisomer S_p_**-2b** and the ditriethylammonium salt of **1** were used.

Conscious that the physicochemical and biological properties of an active pharmaceutical ingredient (API) can be greatly affected by their salt forms,^17^ we decided to look at the possibility to exchange the ditriethylammonium with other cations. To our delight, we successfully converted the triethylammonium salt of prodrug **3a** to the ammonium salt form on a cation exchange resin, obtaining prodrug **6a** in 94% yield (Scheme S1) as confirmed by the disappearance of the triethylamine signals in the ^1^H and ^13^C NMR spectra (see spectra of **6a** in Supporting Information). This result demonstrates that our triphosphate prodrugs are amenable to cation exchange.

To explore the potential applications of this work in the anticancer area, we prepared γ-ProTriP prodrugs of clofarabine (**7**) and gemcitabine (**8**), two FDA-approved anticancer nucleoside analogues (Scheme 2).

**Scheme 2:**
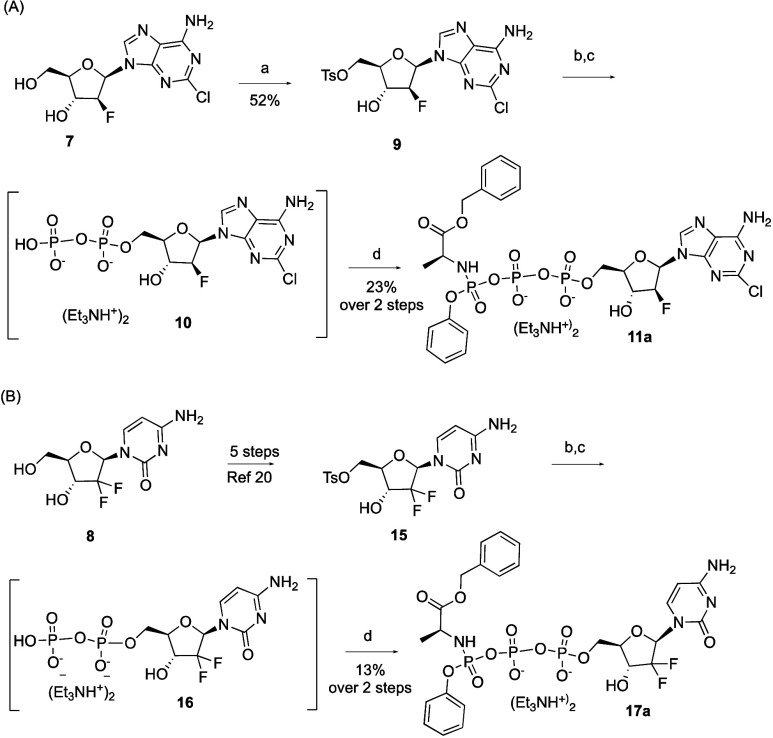
Synthesis of the Triphosphate Prodrugs of Clofarabine **11a** (A) and Gemcitabine **17a** (B) Reaction conditions: (a) TsCl (1.5 equiv), pyridine, 30 °C, 3 h; (b) tris(tetrabutylammonium) hydrogen pyrophosphate (1.5 equiv), CH_3_CN, rt, 2 days; (c) Dowex 50W-X8(H), Et_3_N (2 equiv); d) **2a** (2 equiv), DIPA (2 equiv), DMF, 40 °C (MWI), 3 h.

Briefly for the synthesis of clofarabine γ-ProTriP, C5′ selective tosylation of **7** with tosyl chloride at 30 °C for 3 h yielded **9** in 52%.^18^ Phosphorylation of **9** with tris(tetrabutylammonium) hydrogen pyrophosphate (HPP) afforded clofarabine diphosphate **10** as ditriethylammonium salt after displacement of the tetrabutylammonium salt on a cation exchange resin in the triethylammonium form.^19^ Without any further purification, **10** was reacted with **2a** under the optimized reaction conditions affording triphosphate prodrug **11a**. Despite ^31^P NMR assessment showing excellent conversion of the diphosphate nucleoside **10** into compound **11a** (91%), this prodrug was isolated in 23% yield over two steps after reverse-phase column chromatography (Scheme 2A). Given the good conversion yields calculated by ^31^P NMR for compound **11a** and considering that the diphosphate ditriethylammonium salt **10** was not purified and its yield not calculated, we attribute the low isolated yield of this prodrug to the inefficient conversion of the tosylated intermediate **9** into its diphosphate salt **10**. Similar results were found when gemcitabine diphosphate **16**, prepared by phosphorylation of 5′-tosylate gemcitabine **15** (Scheme S2) with tris(tetrabutylammonium) HPP,^19^ was reacted with **2a** to afford after reverse-phase chromatography prodrug **17a** in 13% yield over two steps (Scheme 2B).^20^ We are currently investigating more efficient methods for the synthesis of nucleoside diphosphates.

A successful prodrug should be sufficiently stable to reach the site of action, where upon activation it will release the biologically active compound. Therefore, we assessed the chemical stability of prodrugs **3a**, **5a**, **11a**, and **17a** in 100 mM phosphate buffer at physiologically relevant pHs (6.5 and 7.4) at 37 °C. The rate of disappearance of the prodrugs was estimated by high-performance liquid chromatography (HPLC), and the half-lives were determined from the apparent first-order rate constant derived from linear regression of pseudo-first-order plots of prodrug concentration versus time. Importantly, all prodrugs were found to be chemically stable at such pHs, as highlighted by long half-lives (Table S1). Prodrugs **3a**, **11a**, and **17a** were also assessed for stability in rat serum showing half-lives of 97, 117, and 97 min, respectively (Table S1, Figure S2). Finally, the preliminary *in vitro* biological activity of this new class of prodrug was evaluated. Growth inhibition assays using several established human solid and liquid tumor cell lines revealed that compound **11a** displayed significant *in vitro* cytotoxicity (Tables S2–S3). These results also suggest cellular penetration by this prodrug and its intracellular activation.

In summary, we have developed a novel and efficient microwave-accelerated synthesis of an unprecedented class of aryloxy phosphoramidate prodrugs called γ-ProTriP containing both purine and pyrimidine triphosphate nucleotides. We further extended this methodology to the preparation of γ-ProTriP of FDA-approved anticancer NAs. These prodrugs proved to be chemically robust at physiologically relevant pHs, while also showing moderate stability in rat serum with clofarabine prodrug **11a** displaying remarkable *in vitro* anticancer activity. Investigations into the activation pathway of this new class of prodrugs are underway and will be reported in due course.

The synthetic methodology reported here can be of extreme significance for those NAs that have shown severe limitations in their activation to give the corresponding NTPs, paving the way for the development of more effective nucleotide-based active drugs and allowing the delivery of NTP analogues as valuable tools for biochemical and medical research.

## Data Availability

The data underlying this study are available in the published article and its Supporting Information.

## Supporting Information Available

Experimental procedures, characterization data, and copies of ^1^H, ^13^C, ^31^P and ^19^F-NMR spectra, 2D spectra HPLC traces and HR-MS for all new compounds (PDF)
