# Supplementary material for: Microwave-Accelerated Synthesis of Novel Triphosphate Nucleoside Prodrugs: Expanding the Therapeutic Arsenal of Anticancer Agents
Source: Org Lett. 2024 Dec 17;27(1):322–7. doi: 10.1021/acs.orglett.4c04379 (PMC11731325; doi:10.1021/acs.orglett.4c04379)
Supplement: Supplementary file 1 — ol4c04379_si_001.pdf [file ol4c04379_si_001.pdf]

Supporting Information

Microwave-accelerated Synthesis of Novel Triphosphate Nucleoside Prodrugs: Expanding the Therapeutic Arsenal of Anticancer Agents

Camille Tisnerat[1], Samuele Di Ciano,[2] Fabrizio Pertusati[1], Michaela Serpi[1*]

[1]School of Chemistry, Cardiff University Main Building, Park Place, CF10 3AT Cardiff, Wales, United Kingdom.

[2]School of Pharmacy and Pharmaceutical Sciences, Redwood Building, King Edwards VII Avenue, CF10 3NB, Cardiff, Wales, United Kingdom.

Table of contents

Crude ^{31}P-NMR Spectrum Example: Figure S1 3

Chemical Stability of Prodrugs 3a, 5a and 11a: Table S1 and Figure S2 4

Biological evaluation of clofarabine (7) and prodrug 11a: TableS2-S3 5

^{31}P-NMR Crude Spectra of Table 1 6

Chemistry 16

- General information 16
- General procedures 17
- Synthesis of the pentafluorophenyl phosphorylating reagents 2a-d 18
- Synthesis of prodrugs 3a-d, 5a, 3b (S_p/R_p 9:1) and 6a 20
- Synthesis of intermediates 9, 10 and Clofarabine prodrug 11a 26
- Synthesis of 5'-tosylate intermediate 15 (Scheme S1) and Gemcitabine prodrug 17a 28

Stability Studies and Biological Evaluation Procedures 32

- Chemical stability in phosphate buffer at pH 6.5 and 7.4 32
- Chemical stability in rat serum 32
- MTS Cell Viability Assay 32

^{1}H- and ^{31}P-NMR spectra of reagents 2a-d 33

^{1}H-, ^{31}P-, ^{13}C-NMR spectra, HRMS spectra and HPLC chromatograms of prodrugs 3a-d, 5a, and 3b (S_p/R_p 9:1) 37

^{1}H-, ^{19}F- and ^{13}C-NMR spectra and MS spectra of intermediates 9-10. 60

^{1}H-, ^{31}P-, ^{19}F-, ^{13}C-NMR spectra, HRMS spectra and HPLC chromatograms of prodrugs 11a 63

^{1}H-, ^{19}F- and ^{13}C-NMR spectra and MS spectra of intermediates 12-16. 67

^{1}H-, ^{31}P-, ^{19}F, ^{13}C-NMR spectra, HRMS spectra and HPLC chromatograms of prodrugs 17a 75

Crude ^{31}P-NMR Spectrum Example: Figure S1

For each experiment, the reaction is monitored by ^{31}P-NMR where the crude is directly solubilised in DMSO-*d*6. As shown on the zoomed spectrum below, the conversion is tracked following the disappearance of the two doublets peaks corresponding to the α and β phosphorus of ADP **1** at δ = -10.8 and -11.3 ppm (in green), and the appearance of the three characteristic signals corresponding to the α, β and γ phosphorus of the triphosphate prodrug **3a** at δ = -7.1 (two doublet peaks), -12.5 (two doublet peaks) and -23.8 (multiplet peak) ppm (in blue). The integration of all these signals and the molar ratio between **1** and **3a** allowed to calculate the conversion percentage.

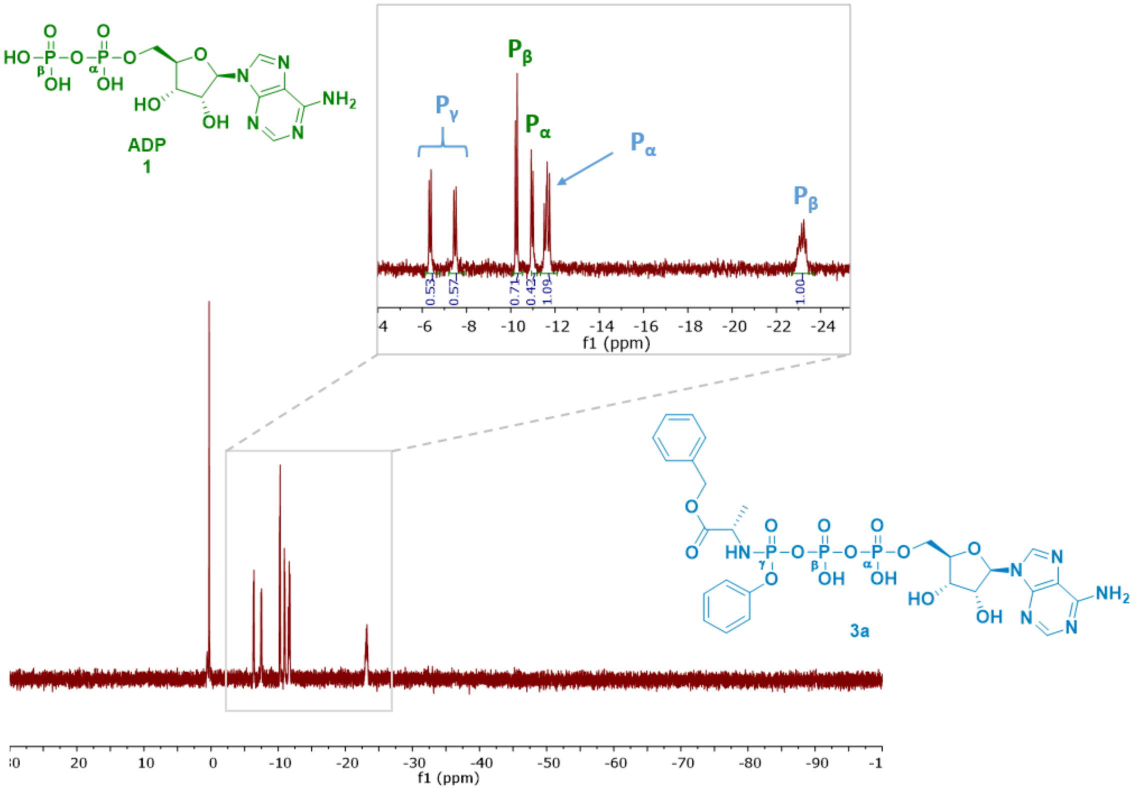

Figure S1. Analysis of ^{31}P-NMR spectrum of crude.

Chemical Stability of Prodrugs 3a, 5a and 11a: Table S1 and Figure S2

Table S1. Half-lives of the prodrugs **3a**, **3e** and **3f** in phosphate buffer (pH 6.5 and pH 7.4) at 37°C and in rat serum.

	Half-life $t_{1/2}$		
	Phosphate Buffer		Rat Serum
**Prodrug**	pH 6.5	pH 7.4	
**3a**	> 6 days (0.90)	> 6 days (0.99)	97 min (0.98)
**5a**	> 6 days (0.95)	> 6 days (0.99)	ND
**11a**	> 6 days (0.95)	> 6 days (0.99)	117 min (0.93)
**17a**	>24h	>24h	97 min (0.96)

R^2 are reported in bracket.

ND: not determined.

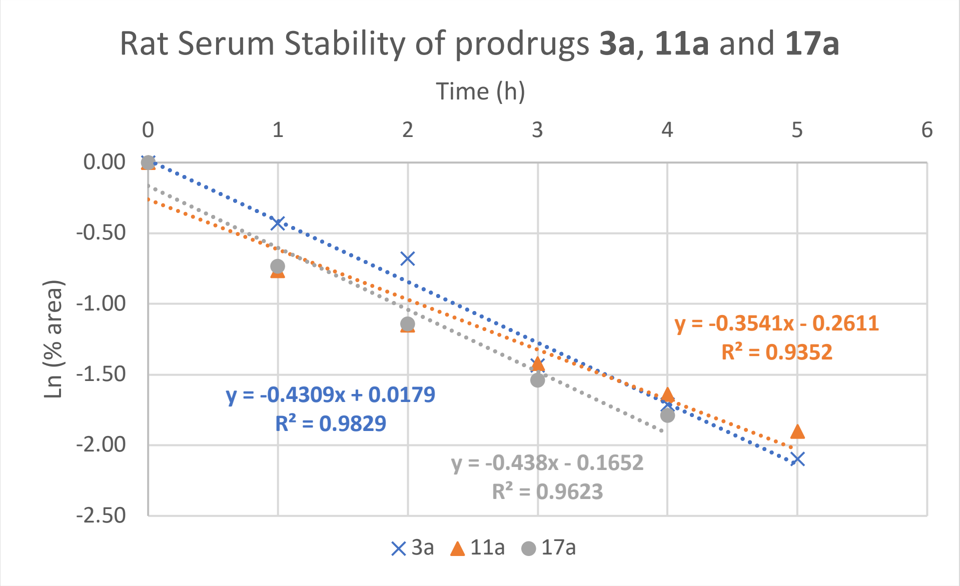

Figure S2. Stability studies of prodrugs **3a**, **11a** and **17a** in rat serum.

Biological evaluation of clofarabine (7) and prodrug 11a: TableS2-S3

Table S2. Cytotoxic activity of Clofarabine (**7**) and prodrug **11a**.

Cpd	HT29		HepG2		OVCAR3		MCF-7	
	IC_{50}	$MI_{\%}$	IC_{50}	$MI_{\%}$	IC_{50}	$MI_{\%}$	IC_{50}	$MI_{\%}$
**Clofarabine**	0.16	68	0.11	57	0.19	42	0.25	54
**11a**	0.23	64	0.15	51	0.40	43	0.37	40
**PTX**	0.003	79	0.006	54	0.03	67	0.002	76

Cytotoxicity data reported as µM IC_{50} values (concentration of drug causing 50% inhibition of cell viability) and MI% values (maximum inhibitory effect of the drug at the range of concentrations considered). PTX: paclitaxel (control); (-) not tested.

Table S3. Cytotoxic activity of clofarabine (**7**) and prodrug **11a**.

Cpd	CCRF-CEM		HL-60		KG-1		MOLT-4		K562		RL		RPMI-8226	
	IC_{50}	$MI_{\%}$	IC_{50}	$MI_{\%}$	IC_{50}	$MI_{\%}$	IC_{50}	$MI_{\%}$	IC_{50}	$MI_{\%}$	IC_{50}	$MI_{\%}$	IC_{50}	$MI_{\%}$
**Clofarabine**	0.01	99	0.04	97	0.06	93	0.04	94	0.11	32	0.08	56	13.31	81
**11a**	0.03	99	0.25	96	0.23	92	0.06	86	1.89	34	3.77	43	15.32	83
**PTX**	0.05	93	0.012	97	0.068	79	0.004	98	0.013	87	0.007	63	0.004	95

Cytotoxicity data reported as µM IC_{50} values (concentration of drug causing 50% inhibition of cell viability) and MI% values (maximum inhibitory effect of the drug at the range of concentrations considered). PTX: paclitaxel (control); (-) not tested

^{31}P-NMR Crude Spectra of Table 1

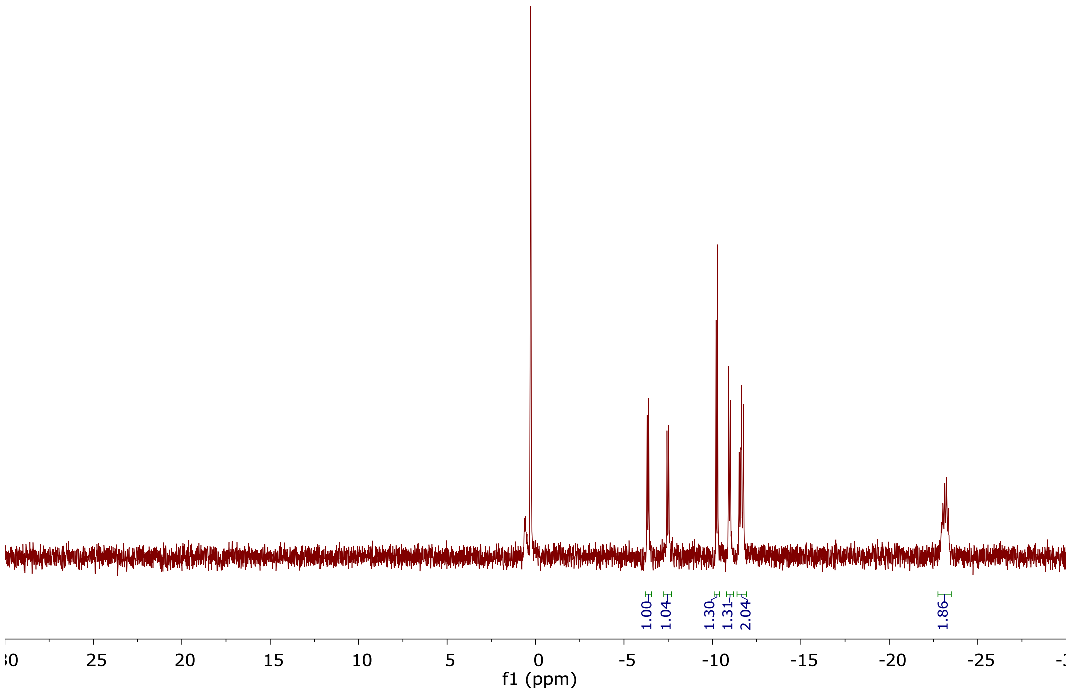

^{31}P-NMR (202 MHz, DMSO-*d6*)

Table 1, entry 1 – Crude (16h)

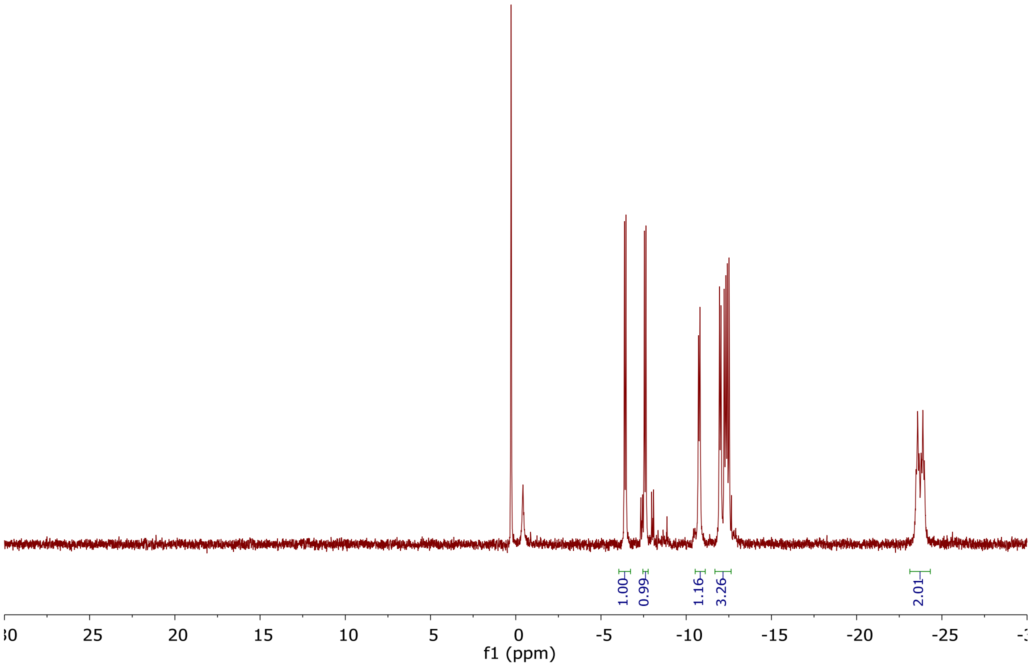

^{31}P-NMR (202 MHz, DMSO-*d6*)

Table 1, entry 5 – Crude (16h)

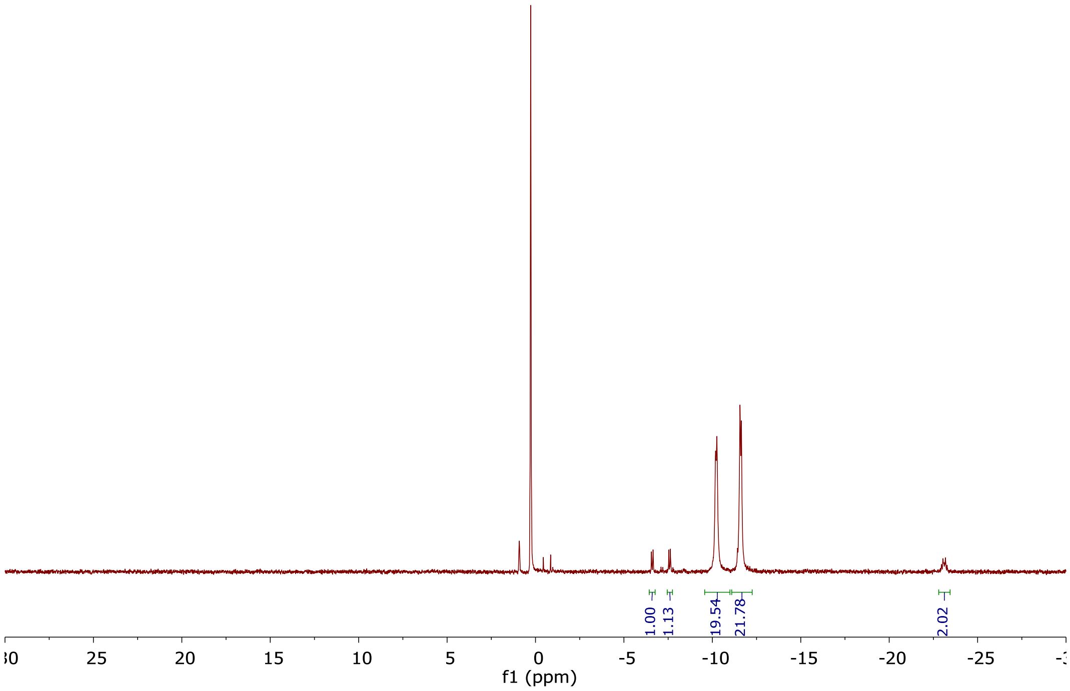

^{31}P-NMR (202 MHz, DMSO-*d*6)

Table 1, entry 7 – Crude (16h)

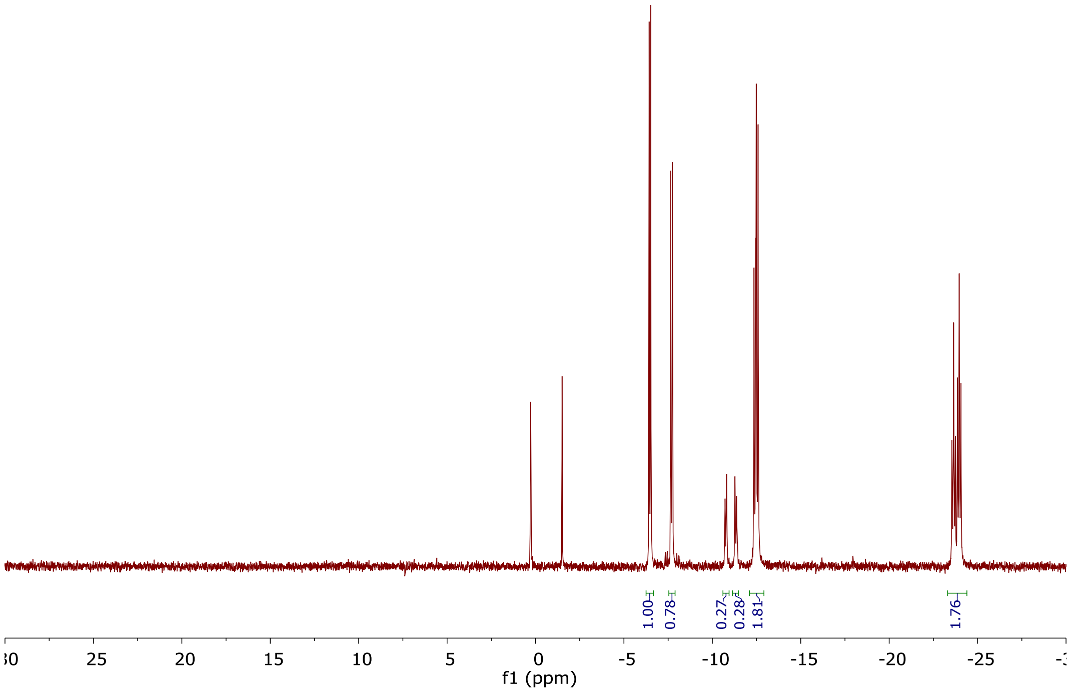

^{31}P-NMR (202 MHz, DMSO-*d*6)

Table 1, entry 9 – Crude (16h)

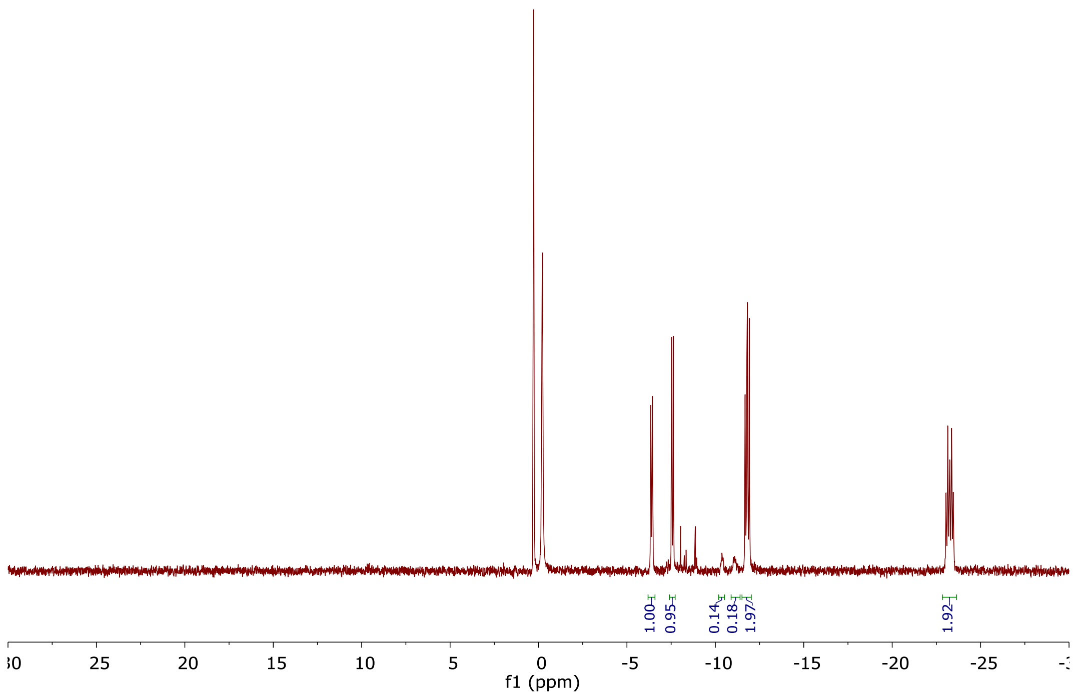

^{31}P-NMR (202 MHz, DMSO-*d*6)
Table 1, entry 10 – Crude (16h)

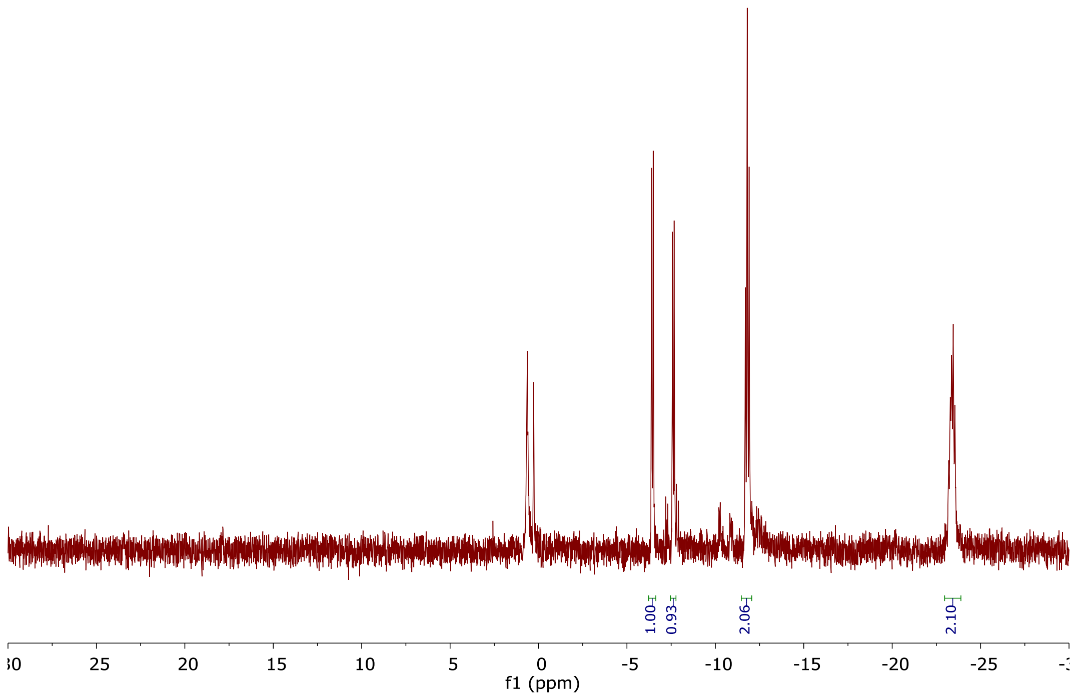

^{31}P-NMR (202 MHz, DMSO-*d*6)

Table 1, entry 11 – Crude (16h)

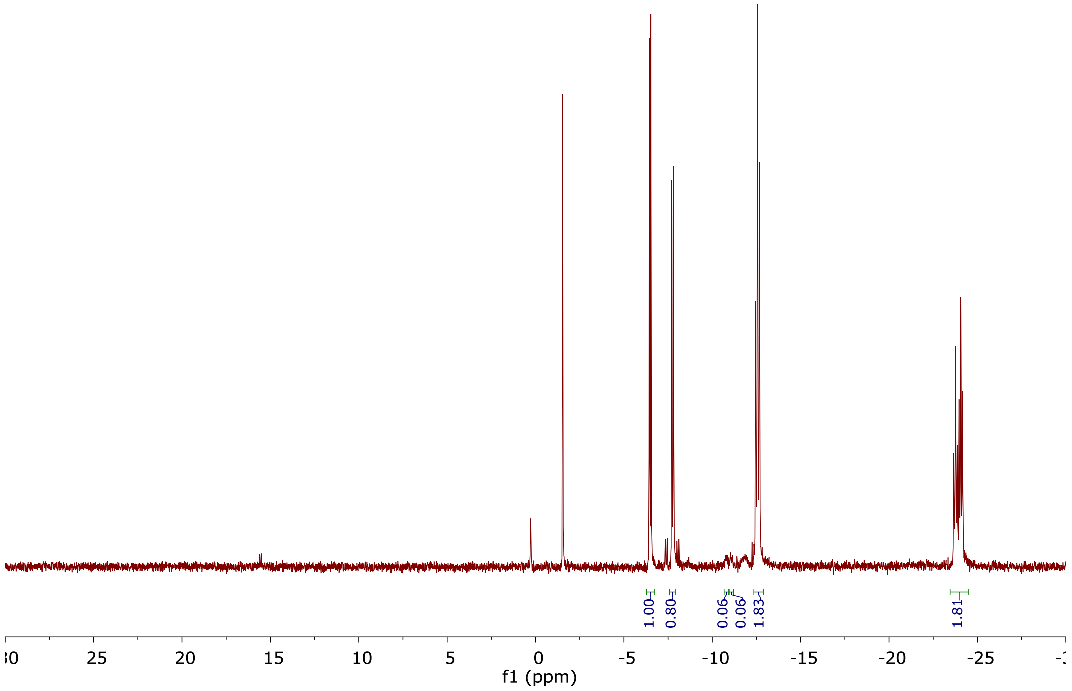

^{31}P-NMR (202 MHz, DMSO-*d*6)
Table 1, entry 12 – Crude (16h)

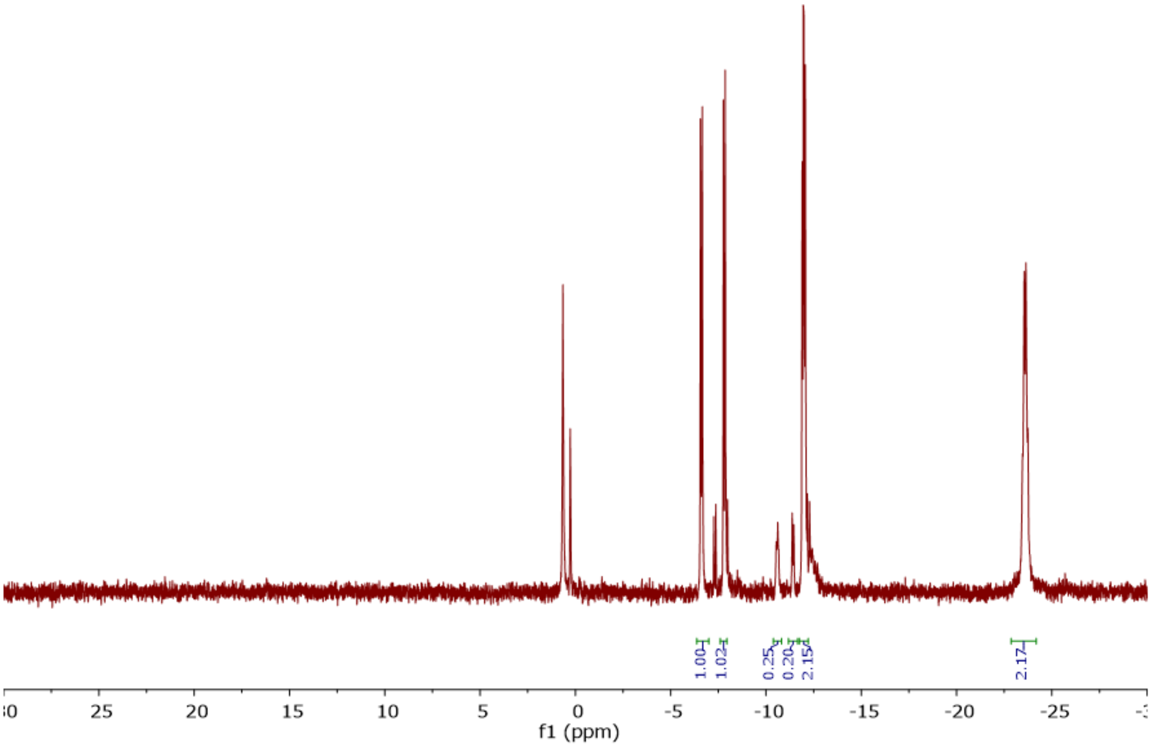

^{31}P-NMR (202 MHz, DMSO-*d*6)

Table 1, entry 13 – Crude (16h)

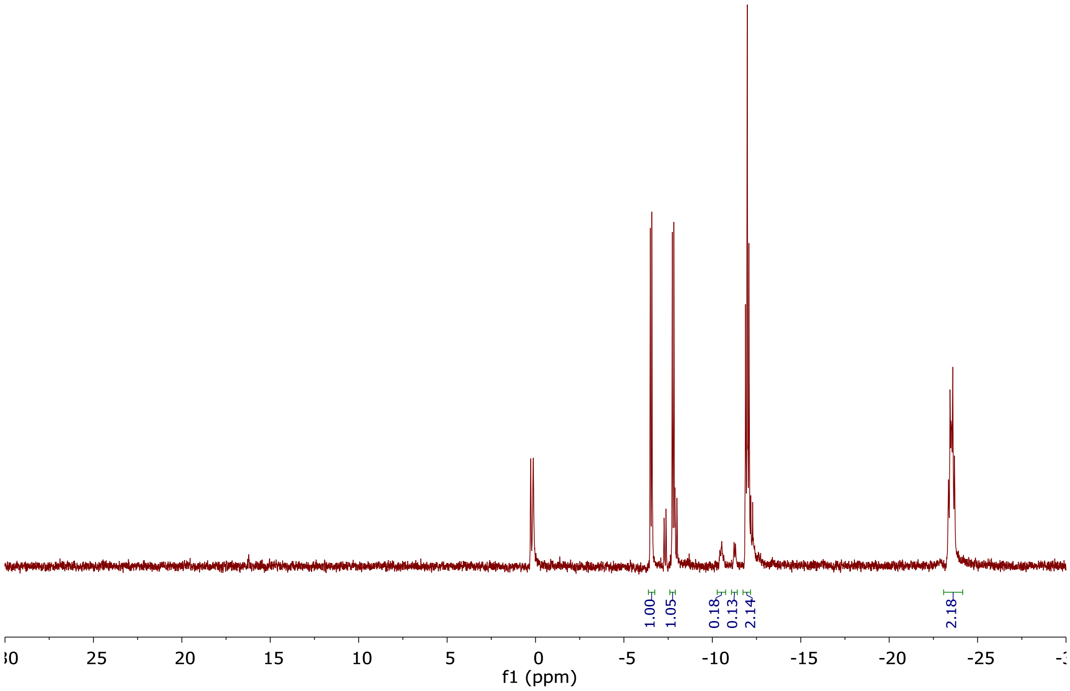

^{31}P-NMR (202 MHz, DMSO-*d*6)

Table 1, entry 14 – Crude (16h)

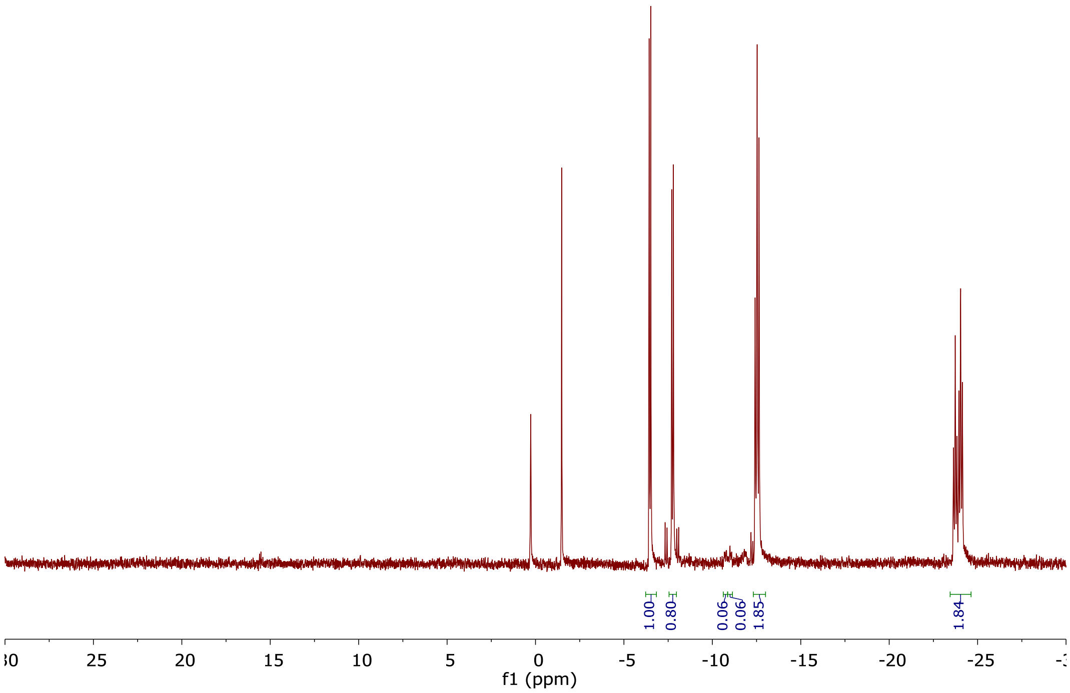

^{31}P-NMR (202 MHz, DMSO-*d*6)

Table 1, entry 15 – Crude (16h)

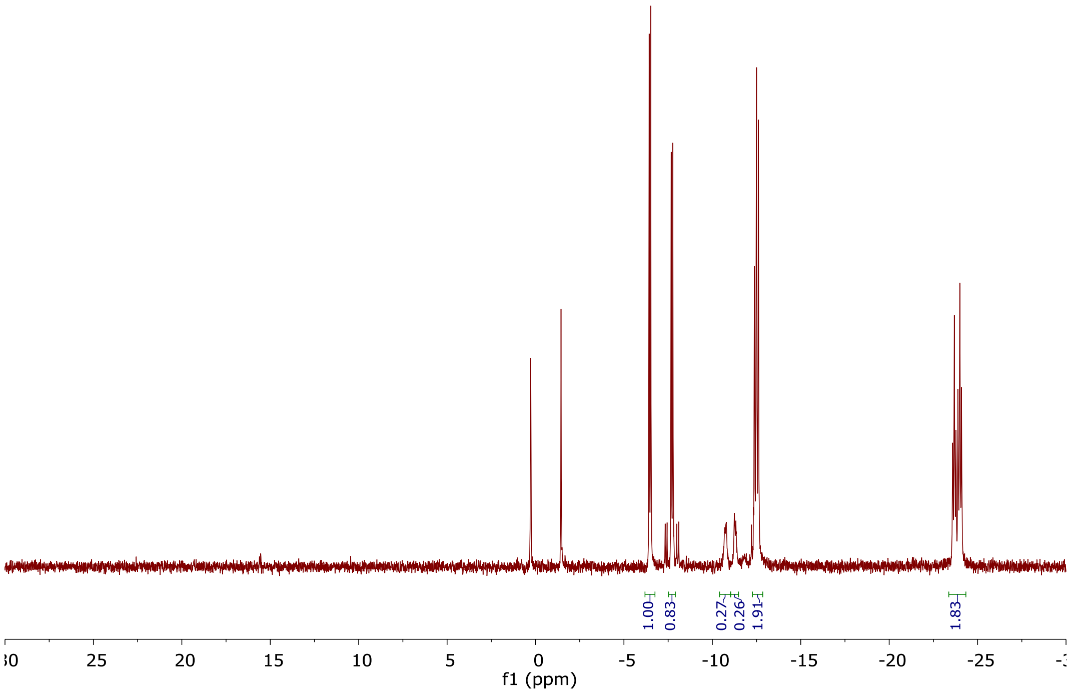

^{31}P-NMR (202 MHz, DMSO-*d6*)

Table 1, entry 16 – Crude (6h)

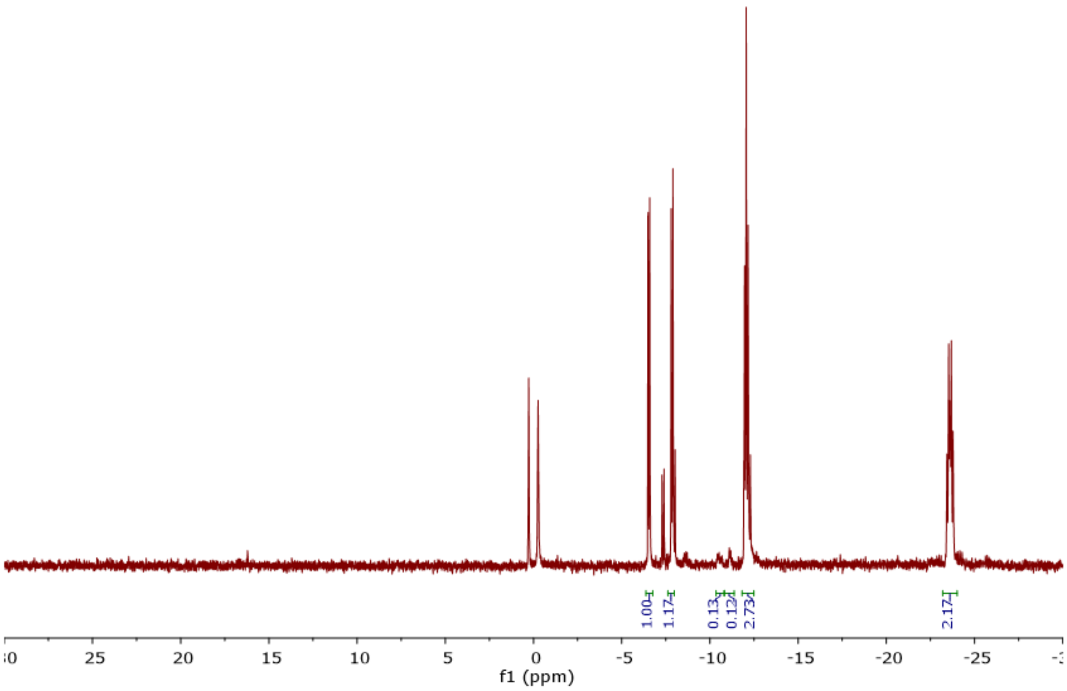

^{31}P-NMR (202 MHz, DMSO-*d6*)

Table 1, entry 17 – Crude (1h)

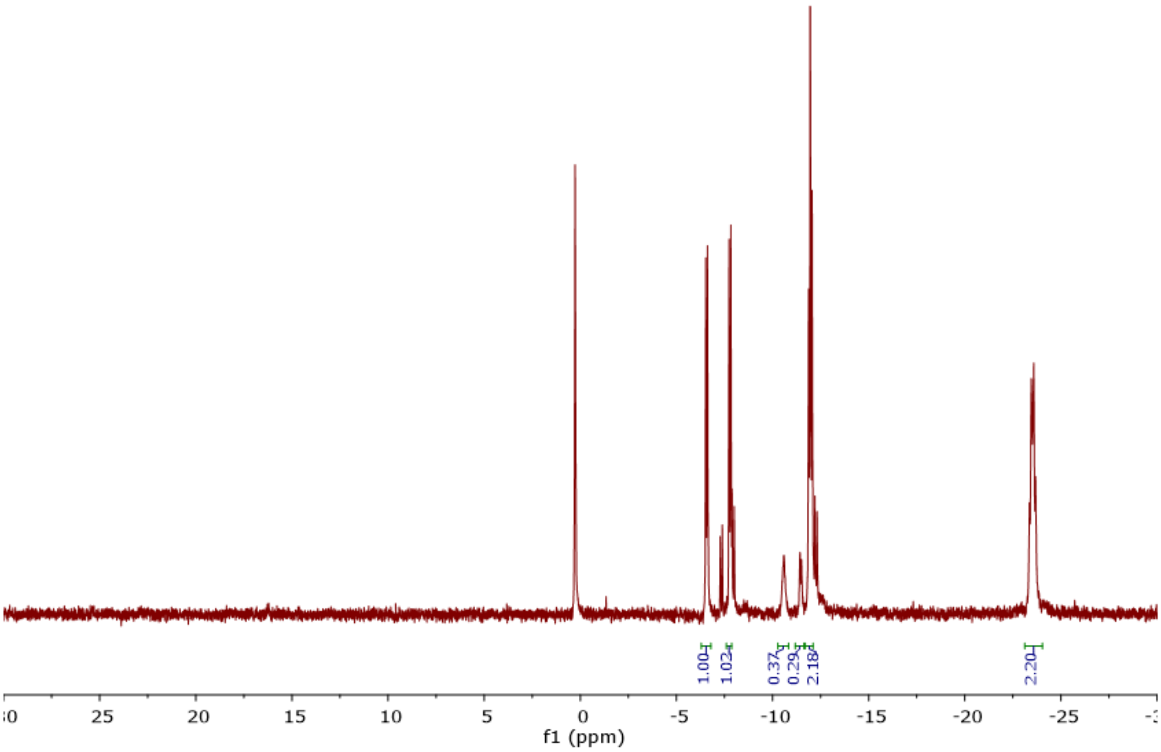

^{31}P-NMR (202 MHz, DMSO-*d*6)

Table 2, entry 18 – Crude (1h)

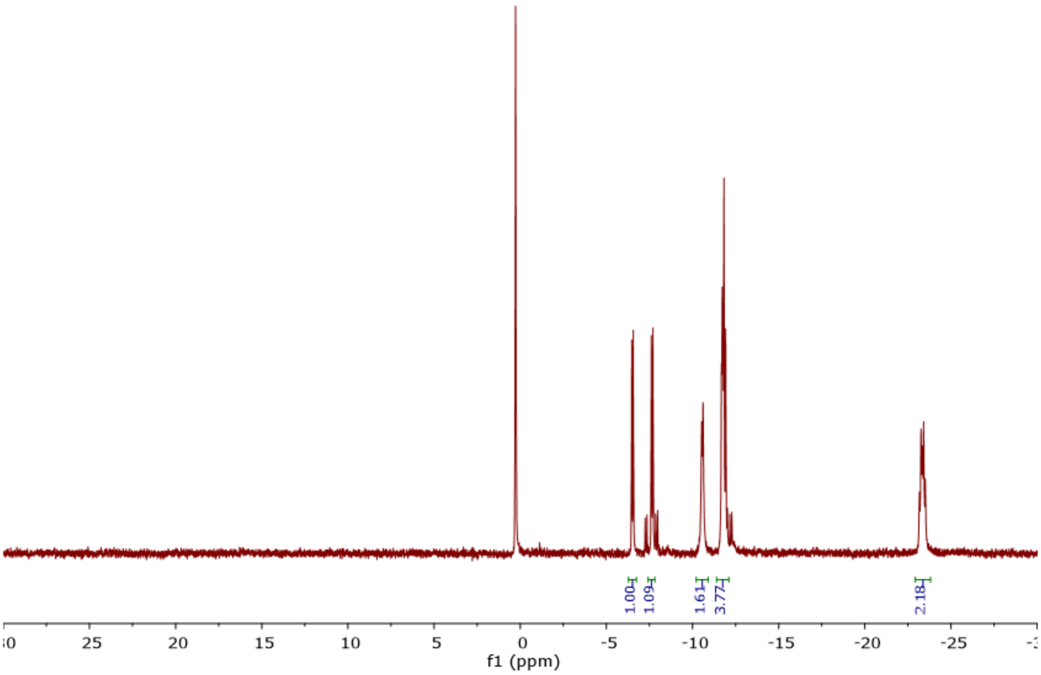

^{31}P-NMR (202 MHz, DMSO-*d*6)

Table 1, entry 19 – Crude (1h)

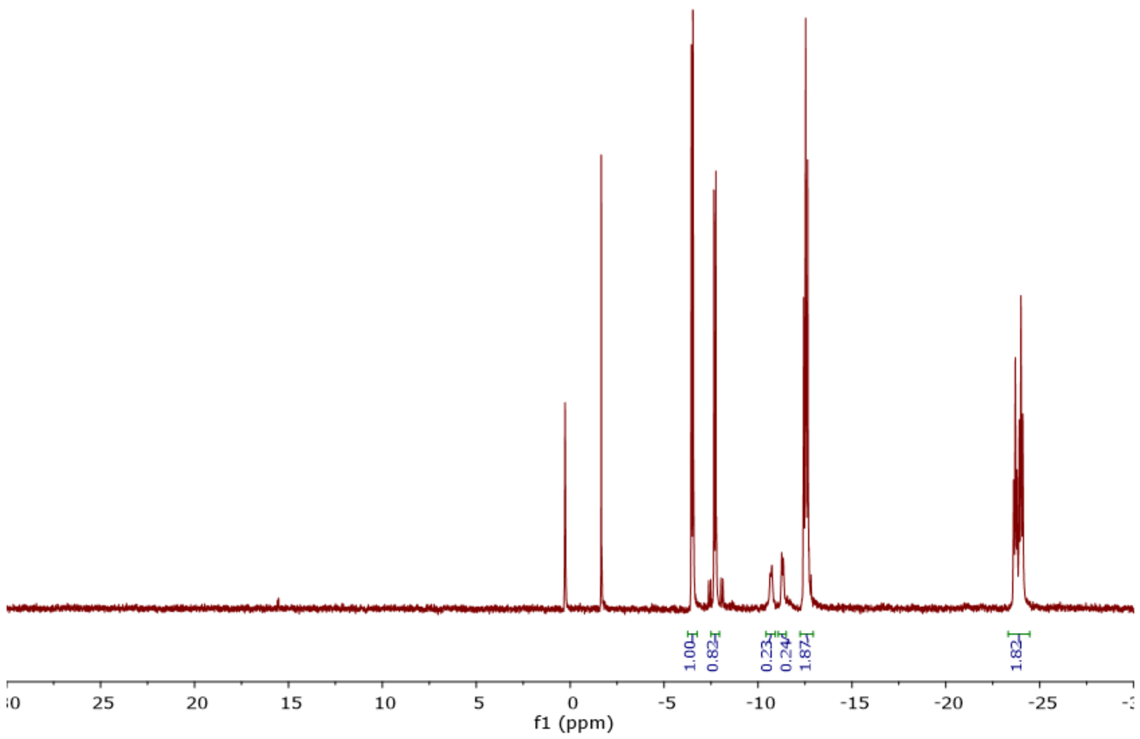

^{31}P-NMR (202 MHz, DMSO-*d*6)

Table 1, entry 20 – Crude (3h)

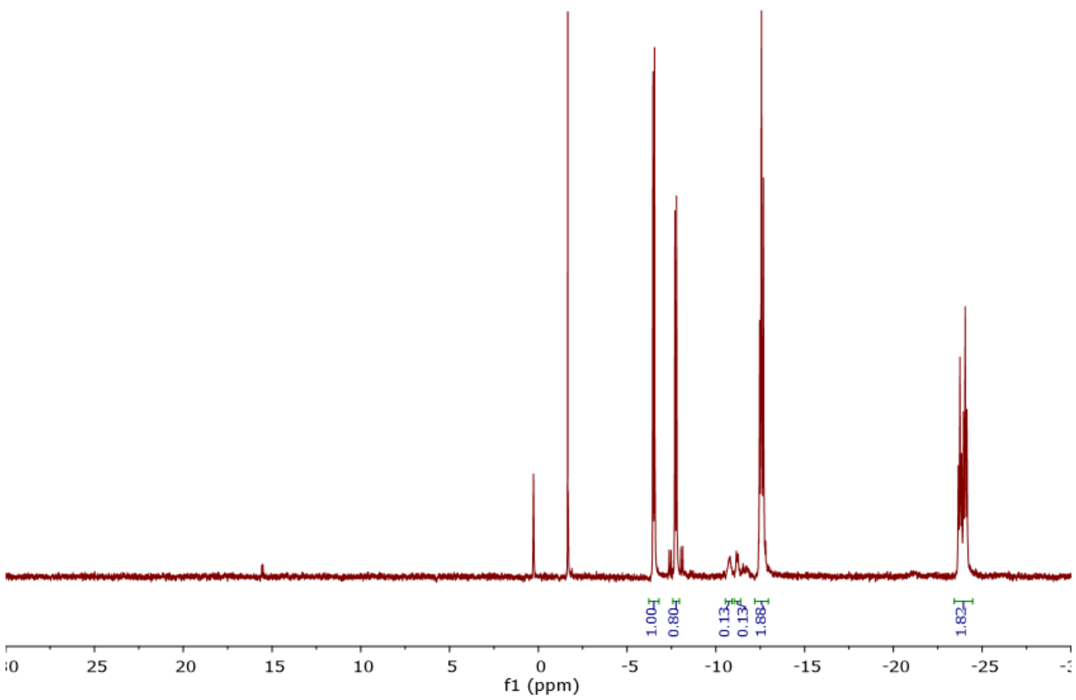

^{31}P-NMR (202 MHz, DMSO-*d*6)

Table 1, entry 20 – Crude (6h)

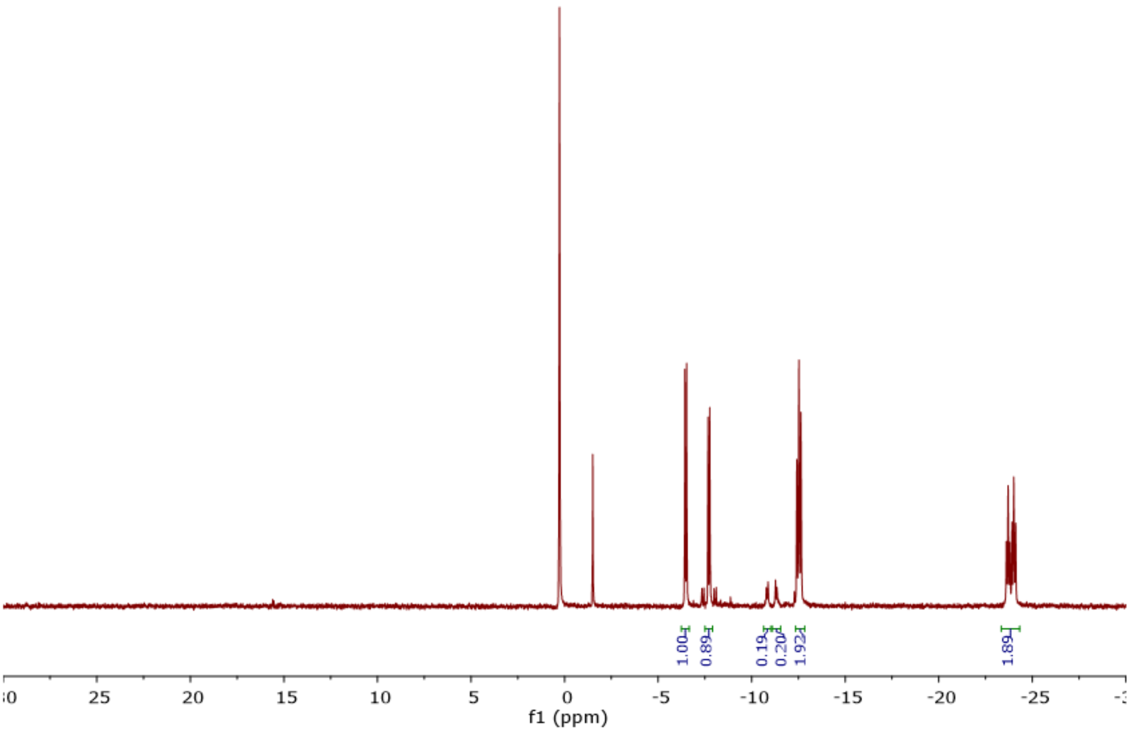

^{31}P-NMR (202 MHz, DMSO-*d*6)

Table 1, entry 21 – Crude (3h)

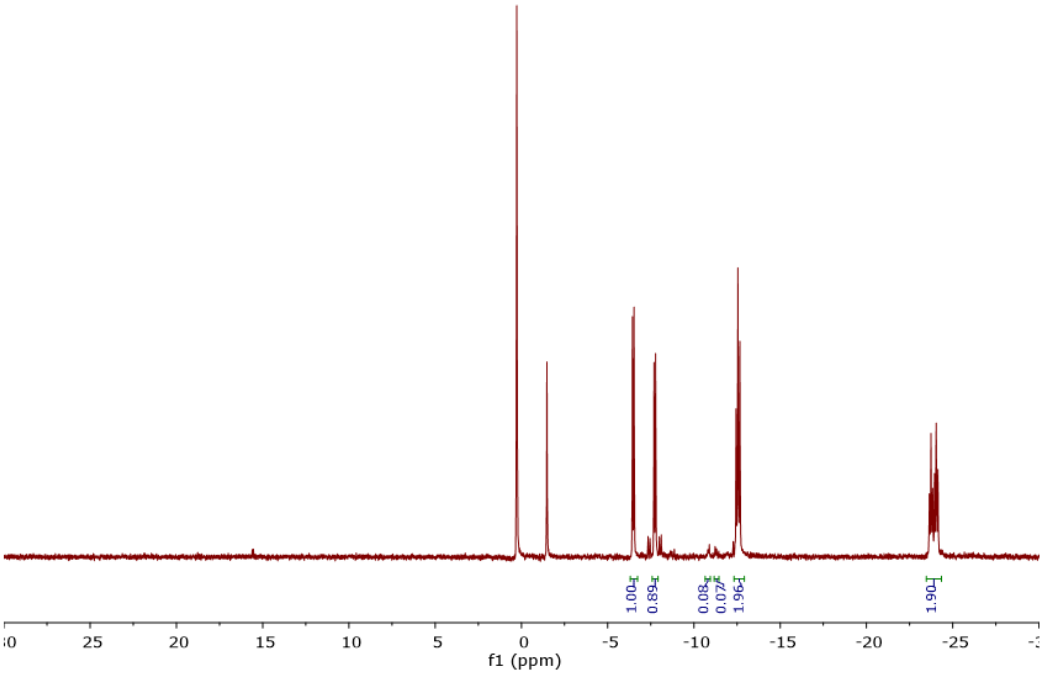

^{31}P-NMR (202 MHz, DMSO-*d*6)

Table 1, entry 21 – Crude (6h)

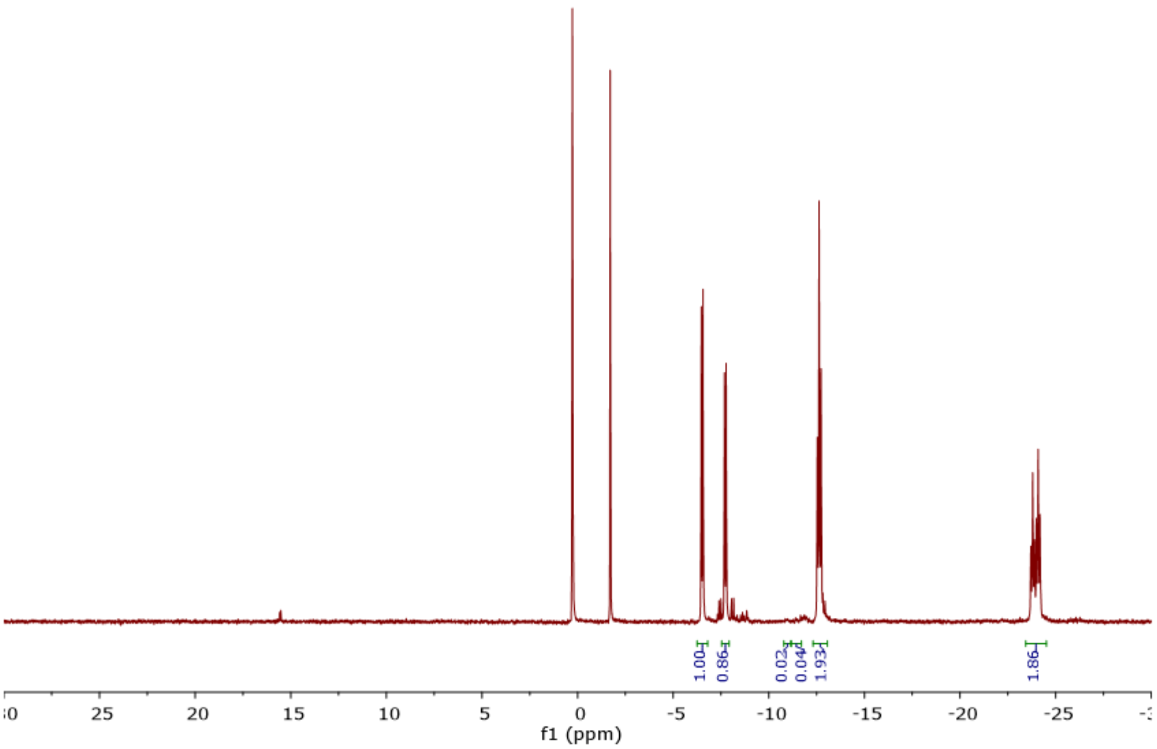

^{31}P-NMR (202 MHz, DMSO-*d*6)

Table 1, entry 22 – Crude (3h)

Chemistry

General information

All solvents used were anhydrous and used as supplied by Sigma-Aldrich. All commercially available reagents were supplied by Sigma-Aldrich, Fisher, Fluorochem, Apollo Scientific, VWR Chemicals, Acros Organics, Carbosynth or Bioplus Chemicals and used without further purification. For analytical thin-layer chromatography (TLC), precoated aluminium-backed plates (60 F-54, 0.2 mm thickness; supplied by Supelco) were used and developed by an ascending elution method. After solvent evaporation, compounds were detected by quenching of the fluorescence at 254 nm upon irradiation with a UV lamp. Column chromatography purifications were carried out by means of automatic Biotage Isolera One. ^{1}H, ^{31}P, ^{13}C and ^{19}F-NMR spectra were recorded in a Bruker Avance 500 spectrometer at 500, 202, 125, and 471 MHz, respectively, or in a Bruker Avance 400 spectrometer at 400, 162, 101 and 376 MHz, respectively, and auto-calibrated to the deuterated solvent reference peak in case of ^{1}H and ^{13}C-NMR. All ^{31}P, ^{13}C and ^{19}F-NMR spectra were proton-decoupled. Chemical shifts are given in parts per million (ppm), and coupling constants (*J*) are measured in Hertz (Hz). The following abbreviations are used in the assignment of NMR signals: s (singlet), d (doublet), t (triplet), q (quartet), m (multiplet), bs (broad singlet), dd (doublet of doublet), ddd (doublet of doublet of doublet), dt (doublet of triplet). The assignment of the signals in ^{1}H-NMR and ^{13}C-NMR was done based on the analysis of coupling constants and additional two-dimensional experiments (correlation spectroscopy (COSY), heteronuclear single-quantum coherence (HSQC)). Analytical high-performance liquid chromatography (HPLC) analysis was performed using Agilent Technologies 1260 Infinity apparatus with Agilent Technologies Zorbax pursuit 3 C18, 150x4.6 mm column. Low-resolution mass spectrometry was performed on a Bruker Daltonics MicroTof-LC system (atmospheric pressure ionization, electron spray mass spectroscopy) in both positive and negative mode. Microwave-irradiation reactions were performed on a Biotage Initiator+, operating at a frequency of 2.45 GHz.

General procedures

General procedure 1 to prepare the pentafluorophenyl phosphorylating reagents (2a-d).

To a solution of the amino ester hydrochloride (1 eq) in dry dichloromethane at -78°C under nitrogen atmosphere were added successively triethylamine (2 eq) and aryl dichlorophosphate (1 eq) dropwise. The reaction mixture was stirred at -78°C for 30 min and then allowed to warm to 0°C over 3 h. To this mixture was added a solution of 2,3,4,5,6-pentafluoro phenol (1 eq) and triethylamine (1.1 eq) in dry dichloromethane dropwise over 15 min. The mixture was allowed to stir at 0°C for 2 h. The white solid (triethylamine hydrochloride) was filtered off and washed with dichloromethane, then diethyl ether. After concentration of the filtrate under reduced pressure, the crude was purified on Biotage Isolera (silica cartridge).

General procedure 2 to prepare the triphosphate phosphoramidate prodrug 3a with conventional heating.

In a round-bottom flask, adenosine diphosphate **1a** (1 eq) and pentafluorophenyl phosphorylating reagent **2a** (1.1 eq) in dry solvent (0.04 or 0.08 M) were suspended in the appropriate solvent under nitrogen atmosphere. Then, a base (2 or 3 eq) was added, and the mixture was stirred overnight at the stipulated temperature. The solvent was removed under reduced pressure and the resulting crude mixture was dissolved in 0.1 M TEAB affording a precipitate. After filtration, the aqueous phase was purified on a reverse phase column chromatography by Biotage Isolera (Biotage SNAP KP-C18-HS 60 g cartridge) with a gradient of acetonitrile in 0.1 M TEAB from 5% to 100% for 40 min) to afford the desired compound **3a**.

General procedure 3 to prepare the triphosphate phosphoramidate prodrugs 3a-d, 3b (S_P/R_p 9:1)**, 5a, 11a and 17a with microwaves.**

Nucleoside diphosphate (1 eq) and pentafluorophenyl phosphorylating reagent (2 eq) were suspended in dry *N,N*-dimethylformamide (0.08 M) under nitrogen atmosphere in a 20mL glass microwave vial, sealed with aluminium cap with a Teflon septum. Then, diisopropylamine (2 eq) was added and the mixture was stirred for 3 h at 40^{0}C under microwave irradiation with continuous irradiation power from 0 to 400W with utilization of the high absorbance level of 400W maximum power. After cooling the reaction mixture to ambient temperature with gas jet cooling, the solvent was removed under reduced pressure and the resulting crude mixture was dissolved in 0.1 M TEAB affording a precipitate. Unless otherwise indicated, after filtration, the aqueous phase was purified on reverse phase column chromatrography by Biotage Isolera (Biotage SNAP KP-C18-HS 60 g cartridge)

with a gradient of acetonitrile in 0.1 M TEAB from 5% to 100% for 40 min) to afford the desired prodrugs.

Synthesis of the pentafluorophenyl phosphorylating reagents 2a-d

(*S*)-2-[-(*S*)-(2,3,4,5,6-Pentafluoro-phenoxy)-phenoxy-phosphorylamino] Propionic Acid Benzyl Ester (2a)

Prepared according to general procedure **1** starting from *L*-alanine benzyl ester hydrochloride (1 eq, 4.64 mmol, 1 g), triethylamine (2 eq, 9.28 mmol, 1.3 mL) and phenyl dichlorophosphate (1 eq, 4.64 mmol, 690 µL) in dichloromethane (6 mL) . Then, from 2,3,4,5,6-pentafluoro phenol (1 eq, 4.64 mmol, 854 mg) and triethylamine (1.1 eq, 5.10 mmol, 710 µL) in dichloromethane (6 mL) to obtain after purification (silica 50 g cartridge, gradient of ethyl acetate in hexanes from 0% to 30%) 1.494 g of desired compound **2a** as a white solid (64%). ^{1}H and ^{31}P NMR are in accordance with ACS Med. Chem. Lett. 2016, *7*, 1197-1201.

(*S*)-2-[-(*S*)-(2,3,4,5,6-Pentafluoro-phenoxy)-phenoxy-phosphorylamino] Propionic Acid Isopropyl Ester (2b)

Prepared according to general procedure **1** starting from *L*-alanine isopropyl ester hydrochloride (1 eq, 5.96 mmol, 1 g), triethylamine (2 eq, 11.92 mmol, 1.7 mL) and phenyl dichlorophosphate (1 eq, 5.96 mmol, 890 µL) in dichloromethane (8 mL) . Then, from 2,3,4,5,6-pentafluoro phenol (1 eq, 5.96 mmol, 1.1 g) and triethylamine (1.1 eq, 6.56 mmol, 914 µL) in dichloromethane (8 mL) to obtain after purification (silica 50 g cartridge, gradient of ethyl acetate in hexanes from 20% to 40%) 992 mg of desired compound **2b** as a white solid (37%). ^{1}H and ^{31}P NMR are in accordance with J. Org. Chem. 2011, *76*, 8311-8319.

Ethyl ((perfluorophenoxy)(phenoxy)phosphoryl)-*L*-leucinate (2c)

Prepared according to general **1** procedure starting from *L*-leucine ethyl ester hydrochloride (1 eq, 5.11 mmol, 1 g), triethylamine (2 eq, 10.22 mmol, 1.4 mL) and phenyl dichlorophosphate (1 eq, 5.11 mmol, 760 µL) in dichloromethane (7 mL). Then, from 2,3,4,5,6-pentafluoro phenol (1 eq, 5.11 mmol, 940 mg) and triethylamine (1.1 eq, 5.62 mmol, 780 µL) in dichloromethane (7 mL) to obtain after purification (silica 50 g cartridge, gradient of ethyl acetate in dichloromethane from 0% to 10%) 1.510 g of desired compound **2c** as a white solid (61%). ^{1}H NMR (500 MHz, $CDCl_3$) δ 7.39 – 7.31 (m, 2H, *H*-Ar), 7.30 – 7.17 (m, 3H, H-Ar), 4.24 – 4.05 (m, 3H, $CHCH_2CH(CH_3)_2$, OCH_2CH_3), 3.86 – 3.68 (m, NH), 1.77 – 1.49 (m, 3H, $CHCH_2CH(CH_3)_2$, $CHCH_2CH(CH_3)_2$), 1.26 (dt, *J* = 9.7, 7.1 Hz, 3H, OCH_2CH_3), 0.95 – 0.87 (m, 6H, $CHCH_2CH(CH_3)_2$). ^{31}P (202 MHz, $CDCl_3$,) δ -1.23 (0.5P), -1.50 (0.5P). ^{13}C NMR (126 MHz, $CDCl_3$,) δ

173.2 (d, J = 5.0 Hz, C=O), 172.98 (d, J = 5.4 Hz, C=O), 150.35 (d, J_{CP} = 7.1 Hz, C-Ar), 150.34 (d, J_{CP} = 7.1 Hz, C-Ar), 142.6 – 142.2 (m, C-F), 140.6 – 140.3 (m, C-F), 140.3 – 139.7 (m, C-F), 139.2 – 138.8 (m, C-F), 138.2 – 137.8 (m, C-F), 137.3 – 136.7 (m, C-F), 129.96 (CH-Ar), 129.95 (CH-Ar), 125.8 (d, J = 1.3 Hz, CH-Ar), 125.7 (d, J = 1.2 Hz, CH-Ar), 120.2 (d, J_{CP} = 4.9 Hz, CH-Ar), 120.1 (d, J_{CP} = 5.1 Hz, CH-Ar), 61.8 (OCH$_2$CH$_3$), 61.7 (OCH$_2$CH$_3$), 53.62 (CHCH$_2$CH(CH$_3$)$_2$), 53.56 (CHCH$_2$CH(CH$_3$)$_2$), 44.14 (d, J_{CP} = 5.7 Hz, CHCH$_2$CH(CH$_3$)$_2$), 44.06 (d, J_{CP} = 6.0 Hz, CHCH$_2$CH(CH$_3$)$_2$), 24.54 (CHCH$_2$$C$H(CH$_3$)$_2$), 24.51, (CHCH$_2$$C$H(CH$_3$)$_2$), 22.7 (CHCH$_2$CH($CH_3$)$_2$), 22.6 (CHCH$_2$CH($CH_3$)$_2$), 22.2 (CHCH$_2$CH($CH_3$)$_2$), 22.0 (CHCH$_2$CH($CH_3$)$_2$), 14.23 (OCH$_2$$CH_3$), 14.21 (OCH$_2$$CH_3$). ^{19}F NMR (471 MHz, CDCl$_3$,) δ -153.04 (d, J = 18.9 Hz), -153.25 (d, J = 19.8 Hz), -159.55 (dt, J = 74.0, 22.1 Hz), -162.18 (dt, J = 40.3, 22.2 Hz). HRMS-ESI (*m/z*): calcd for $C_{20}H_{22}F_5NO_5P$ $[M+H]^+$ 482.1156, found 482.1160.

1-naphtyl dichlorophosphate

To a solution of 1-naphtol (1 eq, 13.87 mmol, 2 g) in diethyl ether (70 mL) at -78°C under nitrogen atmosphere was added phosphorus oxychloride (1 eq, 13.87 mmol, 1.30 mL) dropwise. To this mixture was added triethylamine (1 eq, 13.87 mmol, 1.94 mL) dropwise over 10 min. The reaction mixture was stirred at -78°C for 15 min and then allowed to warm to room temperature over 1 h. The white solid (triethylamine hydrochloride) was filtered off. The filtrate was concentrated under reduced pressure to give the desired product without further purification as a yellow oil (93% yield, 3.367 g). ^{1}H and ^{31}P NMR are in accordance with Current Protocols in Nucleic Acid Chemistry 2013, 15.5.1-15.5.15.

***N*-[(*S*)-(1-Naphthalenyloxy)(2,3,4,5,6-pentafluorophenoxy)phosphinyl]-*L*-alanine phenylmethyl ester (2d)**

Prepared according to general procedure **1** starting from *L*-alanine benzyl ester hydrochloride (1 eq, 4.64 mmol, 1 g), triethylamine (2 eq, 9.28 mmol, 1.3 mL) and α-naphtyl dichlorophosphate (1 eq, 4.64 mmol, 1.219 g) in dichloromethane (6 mL). Then, from 2,3,4,5,6-pentafluoro phenol (1 eq, 4.64 mmol, 854 mg) and triethylamine (1.1 eq, 5.10 mmol, 710 µL) in dichloromethane (6 mL) to obtain after purification (silica 50 g cartridge, gradient of dichloromethane in hexanes from 0% to 100%) 1.093 g of desired compound **2d** as a brown oil (43%). ^{1}H and ^{31}P NMR are in accordance with the patent WO2019053476 (2019).

Synthesis of prodrugs 3a-d, 5a, 3b (S_p/R_p 9:1) and 6a

Benzyl-((((((((2*R*,3*S*,4*R*,5*R*)-5-(6-amino-9*H*-purin-9-yl)-3,4 dihydroxytetrahydrofuran-2-yl)methoxy)(hydroxy)phosphoryl)oxy)(hydroxy)phosphoryl)oxy)(phenoxy)phosphoryl)-*L*-alaninate di-triethylammonium salt (3a)

Prepared according to general procedure **3** starting from adenosine diphosphate **1** (0.12 mmol, 50 mg), pentafluorophenyl phosphorylating reagent **2a** (0.24 mmol, 120 mg) and diisopropylamine (0.24 mmol, 34 µL) in *N*,*N*-dimethylformamide (1.5 mL) to obtain after purification 78 mg of desired compound **3a** as a light yellow oil (68%). ^{1}H NMR (500 MHz, CD_3OD) δ 8.54 (d, *J* = 1.8 Hz, 1H, *H*-8), 8.19 (d, *J* = 1.5 Hz, 1H, *H*-2), 7.35 – 7.20 (m, 9H, Ar-*H*), 7.13 – 7.06 (m, 1H, Ar-*H*), 6.08 (dd, *J* = 5.8, 4.6 Hz, 1H, *H*-1'), 5.10 – 5.06 (m, 2H, OCH_2Ph), 4.69 (dd, *J* = 5.7, 5.3 Hz, 0.5H, *H*-2'), 4.64 (dd, *J* = 5.4, 5.4 Hz, 0.5H, *H*-2'), 4.51 – 4.45 (m, 1H, *H*-3'), 4.35 – 4.19 (m, 3H, *H*-4', *H*-5'), 4.21 – 4.08 (m, 1H, C*H*CH_3), 2.97 (q, *J* = 7.3 Hz, 12H, $CH_3CH_2NH^+$), 1.45 (dd, *J* = 7.2, 0.9 Hz, 1.5H, $CHCH_3$), 1.33 (dd, *J* = 7.1, 0.9 Hz, 1.5H, $CHCH_3$), 1.20 (t, *J* = 7.3 Hz, 18H, $CH_3CH_2NH^+$). ^{31}P NMR (202 MHz, CD_3OD) δ -7.52 (d, *J* = 18.2 Hz, 0.5P), -8.17 (d, *J* = 19.1 Hz, 0.5P), -11.74 (d, *J* = 20.7 Hz, 0.5P), -11.77 (d, *J* = 20.0 Hz, 0.5P), -23.81 – (-25.02) (m, 1P). ^{13}C NMR (126 MHz, CD_3OD) δ 174.9 (d, J_{CP} = 6.1 Hz, *C*=O), 174.8 (d, J_{CP} = 7.1 Hz, *C*=O), 157.3 (*C*-2), 153.82 (*C*-6), 153.80 (*C*-6), 152.4 (d, J_{CP} = 6.8 Hz, *C*-Ar *ipso* OP), 152.3 (d, J_{CP} = 6.4 Hz, *C*-Ar *ipso* OP), 151.0 (*C*-4), 150.9 (*C*-4), 141.13 (*C*-8), 141.10 (*C*-8), 137.40 (*C*-Ar), 137.38 (*C*-Ar), 130.52 (*C*H-Ar), 130.52 (*C*H-Ar), 130.49 (*C*H-Ar), 130.48 (*C*H-Ar), 129.5 (*C*H-Ar), 129.2 (*C*H-Ar), 125.81 (*C*H-Ar), 125.79 (*C*H-Ar), 125.74 (*C*H-Ar), 125.73 (*C*H-Ar), 121.95 (d, J_{CP} = 4.8 Hz, *C*H-Ar), 121.91 (d, J_{CP} = 4.8 Hz, *C*H-Ar), 120.17 (*C*-5), 120.14 (*C*-5), 88.9 (*C*-1'), 88.8 (*C*-1'), 85.7 (d, J_{CP} = 8.8 Hz, *C*-4'), 85.6 (d, J_{CP} = 8.7 Hz, *C*-4'), 76.13 (*C*-2'), 76.09 (*C*-2'), 72.16 (*C*-3'), 71.96 (*C*-3'), 67.73 (O*C*H_2Ph), 67.69 (O*C*H_2Ph), 66.8 (d, J_{CP} = 5.9 Hz, *C*-5'), 66.7 (d, J_{CP} = 6.0 Hz, *C*-5'), 51.9 (d, J_{CP} = 2.7 Hz, *C*HCH_3), 51.6 (d, J_{CP} = 1.5 Hz, *C*HCH_3), 47.31 ($CH_3CH_2NH^+$), 20.81 ($CHCH_3$), 20.76 ($CHCH_3$), 9.74 ($CH_3CH_2NH^+$). HRMS-ESI (*m/z*): calcd for $C_{26}H_{32}N_6O_{14}P_3$ $[M-(Et_3N)_2+H]^+$ 745.1190, found 745.1189. HPLC Reversed-phase eluting with TEAB buffer/CH_3CN from 10/90 to 100/0 in 30 minutes, F = 1 mL/min, λ = 264 nm, t_R 8.507 min, purity 95%.

Isopropyl ((((((((2*R*,3*S*,4*R*,5*R*)-5-(6-amino-9*H*-purin-9-yl)-3,4-dihydroxytetrahydrofuran-2-yl)methoxy)(hydroxy)phosphoryl)oxy)(hydroxy)phosphoryl)oxy)(phenoxy)phosphoryl)-*L*-alaninate di-triethylammonium salt (3b)

Prepared according to general procedure **3** starting from adenosine diphosphate **1** (0.12 mmol, 50 mg), pentafluorophenyl phosphorylating reagent **2b** (0.24 mmol, 109 mg) and diisopropylamine (0.24 mmol, 34 µL) in *N*,*N*-dimethylformamide (1.5 mL) to obtain after purification 67 mg of desired compound **3b** as a colourless oil (62%). ^{1}H NMR (500 MHz, CD_3OD) δ 8.56 (s, 1H, *H*-8), 8.20 (s, 1H, *H*-2), 7.34 – 7.25 (m, 9H, Ar-*H*), 7.16 – 7.06 (m, 1H, Ar-*H*), 6.08 (dd, *J* = 5.7, 4.7 Hz, 1H, *H*-1'), 4.97 – 4.87

(m, 1H, OC*H*(CH_3)$_2$), 4.69 (dd, *J* = 5.5, 5.5 Hz, 0.5H, *H*-2'), 4.65 (dd, *J* = 5.3, 5.3 Hz, 0.5H, *H*-2'), 4.51 – 4.44 (m, 1H, *H*-3'), 4.34 – 4.18 (m, 3H, *H*-4', *H*-5'), 4.09 – 3.97 (m, 1H, C*H*CH_3), 3.16 (q, *J* = 7.3 Hz, 12H, CH_3C*H*$_2$$NH^+$), 1.42 (dd, *J* = 7.1, 0.9 Hz, 1.5H, CHC*H*$_3$), 1.31 (dd, *J* = 7.1, 0.8 Hz, 1.5H, CHC*H*$_3$), 1.28 (t, *J* = 7.3 Hz, 18H, C*H*$_3$$CH_2NH^+$), 1.22 – 1.15 (m, 6H, OCH(C*H*$_3$)$_2$). ^{31}P NMR (202 MHz, CD_3OD) δ -7.48 (d, *J* = 18.2 Hz, 0.5P), -8.13 (d, *J* = 19.0 Hz, 0.5P), -11.75 (d, *J* = 20.4 Hz, 0.5P), -11.79 (d, *J* = 19.9 Hz, 0.5P), -24.26 – (-24.61) (m, 1P). ^{13}C NMR (126 MHz, CD_3OD) δ 174.7 (d, J_{CP} = 6.6 Hz, *C*=O), 174.5 (d, J_{CP} = 7.7 Hz, *C*=O), 157.0 (*C*-2), 153.51 (*C*-6), 153.48 (*C*-6), 152.4 (d, J_{CP} = 7.2 Hz, *C*-Ar *ipso* OP), 152.3 (d, J_{CP} = 7.1 Hz, *C*-Ar), 150.9 (*C*-4), 150.8 (*C*-4), 141.22 (*C*-8), 141.20 (*C*-8), 130.53 (*C*H-Ar), 130.51 (*C*H-Ar), 130.50 (*C*H-Ar), 125.82 (*C*H-Ar), 125.82 (*C*H-Ar), 125.77 (*C*H-Ar), 125.76 (*C*H-Ar), 121.93 (d, J_{CP} = 4.8 Hz, *C*H-Ar), 121.87 (d, J_{CP} = 4.9 Hz, *C*H-Ar), 120.14 (*C*-5), 88.9 (*C*-1'), 88.8 (*C*-1'), 85.65 (d, J_{CP} = 9.0 Hz, *C*-4'), 85.58 (d, J_{CP} = 8.8 Hz, *C*-4'), 76.15 (*C*-2'), 76.11 (*C*-2'), 72.1 (*C*-3'), 71.9 (*C*-3'), 69.92 (O*C*H(CH_3)$_2$), 69.87 (O*C*H(CH_3)$_2$), 66.8 (d, J_{CP} = 5.7 Hz, *C*-5'), 66.7 (d, J_{CP} = 5.7 Hz, *C*-5'), 51.9 (d, J_{CP} = 2.7 Hz, *C*HCH_3), 51.6 (d, J_{CP} = 2.0 Hz, *C*HCH_3), 47.4 (CH_3*C*H$_2$$NH^+$), 22.0 (OCH(*C*H$_3$)$_2$), 21.95 (OCH(*C*H$_3$)$_2$), 21.88 (OCH(*C*H$_3$)$_2$), 20.91 (d, J_{CP} = 6.3 Hz, CH*C*H$_3$), 20.87 (d, J_{CP} = 5.7 Hz, CH*C*H$_3$), 9.1 (*C*H$_3$$CH_2NH^+$). HRMS-ESI (*m/z*): calcd for $C_{22}H_{32}N_6O_{14}P_3$ $[M-(Et_3N)_2+H]^+$ 697.1189, found 697.1191. HPLC Reversed-phase eluting with TEAB buffer/CH_3CN from 10/90 to 100/0 in 30 minutes, F = 1 mL/min, λ = 254 nm, t_R 9.766 min, purity 93%.

Ethyl (((((((2*R*,3*S*,4*R*,5*R*)-5-(6-amino-9*H*-purin-9-yl)-3,4-dihydroxytetrahydrofuran-2-yl)methoxy)(hydroxy)phosphoryl)oxy)(hydroxy)phosphoryl)oxy)(phenoxy)phosphoryl)-*L*-leucinate di-triethylammonium salt (3c)

Prepared according to general procedure **3** starting from adenosine diphosphate **1** (0.12 mmol, 50 mg), pentafluorophenyl phosphorylating reagent **2c** (0.24 mmol, 115 mg) and diisopropylamine (0.24 mmol, 34 µL) in *N*,*N*-dimethylformamide (1.5 mL) to obtain after purification 77 mg of desired compound **3c** as a colourless oil (69%). ^{1}H NMR (500 MHz, CD_3OD) δ 8.57 (s, 1H, *H*-8), 8.21 (s, 1H, *H*-2), 7.32 – 7.24 (m, 4H, Ar-*H*), 7.15 – 7.07 (m, 1H, Ar-*H*), 6.08 (dd, *J* = 5.7, 4.6 Hz, 1H, *H*-1'), 4.68 (dd, *J* = 5.4, 5.4 Hz, 0.5H, *H*-2'), 4.63 (dd, *J* = 5.3, 5.3 Hz, 0.5H, *H*-2'), 4.51 – 4.43 (m, 1H, *H*-3'), 4.34 – 4.20 (m, 3H, *H*-4', *H*-5'), 4.15 – 4.07 (m, 1H, OC*H*$_2$$CH_3$), 4.03 (q, *J* = 7.1 Hz, 1H, OC*H*$_2$$CH_3$), 3.97 (ddd, *J* = 9.8, 8.1, 6.7 Hz, 0.5H, C*H*$CH_2CH(CH_3)_2$), 3.91 (ddd, *J* = 9.2, 7.9, 6.5 Hz, 0.5H, C*H*$CH_2CH(CH_3)_2$), 3.16 (q, *J* = 7.3 Hz, 12H, CH_3C*H*$_2$$NH^+$), 1.82 – 1.73 (m, 0.5H, $CHCH_2$C*H*(CH_3)$_2$), 1.65 – 1.48 (m, 1.5H, CHC*H*$_2$C*H*(CH_3)$_2$), 1.48 – 1.43 (m, 1H, CHC*H*$_2$CH(CH_3)$_2$), 1.28 (t, *J* = 7.3 Hz, 18H, C*H*$_3$$CH_2NH^+$), 1.22 (t, *J* = 7.1 Hz, 1.5H, CH_2C*H*$_3$), 1.17 (t, *J* = 7.1 Hz, 1.5H, CH_2C*H*$_3$), 0.89 (dd, *J* = 6.6, 4.8 Hz, 3H, $CHCH_2CH$(C*H*$_3$)$_2$), 0.81 (d, *J* = 6.4 Hz, 1.5H, $CHCH_2CH$(C*H*$_3$)$_2$), 0.76 (d, *J* = 6.4 Hz, 1.5H, $CHCH_2CH$(C*H*$_3$)$_2$). ^{31}P NMR (202 MHz, CD_3OD) δ -7.43 (d, *J* = 18.4 Hz, 0.5P), -7.57 (d, *J* = 18.5 Hz, 0.5P), -11.69 (d, *J* = 19.7 Hz, 0.5P), -11.74 (d, *J* = 19.6 Hz, 0.5P), -24.37 (d, *J* = 19.9 Hz, 0.5P), -24.52 (d, *J* = 19.6 Hz, 0.5P). ^{13}C NMR (126 MHz, CD_3OD) δ 175.0 (d, J_{CP} = 7.1 Hz, *C*=O), 174.9 (d, J_{CP} = 6.9 Hz, *C*=O), 156.62 (*C*-6), 156.58 (*C*-6), 152.95 (*C*-2), 152.88 (*C*-2), 152.45

(d, J_{CP} = 7.3 Hz, *C*-Ar *ipso* OP), 152.41 (d, J_{CP} = 6.9 Hz, *C*-Ar *ipso* OP), 150.82 (*C*-4), 150.75 (*C*-4), 141.4 (*C*-8), 130.52 (*C*H-Ar), 130.51 (*C*H-Ar), 130.50 (*C*H-Ar), 130.49 (*C*H-Ar), 125.85 (*C*H-Ar), 125.84 (*C*H-Ar), 125.73 (*C*H-Ar), 125.71 (*C*H-Ar), 122.0 (d, J_{CP} = 4.8 Hz, *C*H-Ar), 121.9 (d, J_{CP} = 4.9 Hz, *C*H-Ar), 120.1 (*C*-5), 89.00 (*C*-1'), 88.9 (*C*-1'), 85.64 (d, J_{CP} = 9.1 Hz, *C*-4'), 85.60 (d, J_{CP} = 9.1 Hz, *C*-4'), 76.22 (*C*-2'), 76.16 (*C*-2'), 72.1 (*C*-3'), 71.9 (*C*-3'), 66.72 (d, J_{CP} = 6.5 Hz, *C*-5'), 66.67 (d, J_{CP} = 7.1 Hz, *C*-5'), 62.1 (O*C*$H_2$$CH_3$), 62.0 (O*C*$H_2$$CH_3$), 54.63 (d, J_{CP} = 9.1 Hz, *C*HCH_2CH$(CH_3)_2$), 54.62 (d, J_{CP} = 7.9 Hz, *C*HCH_2CH$(CH_3)_2$), 47.4 (CH_3*C*H_2NH^+), 44.6 (d, J_{CP} = 7.1 Hz, CH*C*H_2CH$(CH_3)_2$), 44.4 (d, J_{CP} = 7.1 Hz, CH*C*H_2CH$(CH_3)_2$), 25.5 (CHCH_2*C*H$(CH_3)_2$), 25.4 (CHCH_2*C*H$(CH_3)_2$), 23.02 (CHCH_2CH$(CH_3)_2$), 22.97 (CHCH_2CH$(CH_3)_2$), 22.5 (CHCH_2CH$(CH_3)_2$), 22.3 (CHCH_2CH$(CH_3)_2$), 14.5 (OCH_2*C*H_3), 14.4 (OCH_2*C*H_3), 9.1 (*C*$H_3$$CH_2NH^+$). HRMS-ESI (*m/z*): calcd for $C_{24}H_{36}N_6O_{14}P_3$ $[M-(Et_3N)_2+H]^+$ 725.1502, found 725.1508. HPLC Reversed-phase eluting with TEAB buffer/CH_3CN from 10/90 to 100/0 in 30 minutes, F = 1 mL/min, λ = 254 nm, t_R 11.120 min (fast eluting isomer) and 11.383 min (slow eluting isomer), purity 95%.

Benzyl (((((((2*R*,3*S*,4*R*,5*R*)-5-(6-amino-9*H*-purin-9-yl)-3,4-dihydroxytetrahydrofuran-2-yl)methoxy)(hydroxy)phosphoryl)oxy)(hydroxy)phosphoryl)oxy)(naphthalen-1-yloxy)phosphoryl)-*L*-alaninate di-triethylammonium salt (3d)

Prepared according to general procedure **3** starting from adenosine diphosphate **1** (0.23 mmol, 100 mg), pentafluorophenyl phosphorylating reagent **2d** (0.46 mmol, 254 mg) and diisopropylamine (0.46 mmol, 64 µL) in *N*,*N*-dimethylformamide (3 mL) to obtain after two purifications 105 mg of desired compound **3d** as a colourless oil (46%). ^{1}H NMR (500 MHz, CD_3OD) δ 8.54 (d, *J* = 6.5 Hz, 1H, *H*-8), 8.39 – 8.26 (m, 1H, Ar-*H*), 8.18 (d, *J* = 0.7 Hz, 1H, *H*-2), 7.87 – 7.78 (m, 1H, Ar-*H*), 7.67 – 7.56 (m, 2H, Ar-*H*), 7.51 – 7.43 (m, 2H, Ar-*H*), 7.41 – 7.31 (m, 1H, Ar-*H*), 7.29 – 7.16 (m, 5H, Ar-*H*), 6.07 (dd, *J* = 5.4, 5.4 Hz, 1H, *H*-1'), 5.02 – 4.91 (m, 2H, OC*H*$_2$Ph), 4.68 (dd, *J* = 5.4, 5.4 Hz, 0.5H, *H*-2'), 4.64 (dd, *J* = 5.4, 5.4 Hz, 0.5H, *H*-2'), 4.47 (ddd, *J* = 6.3, 5.0, 3.4 Hz, 1H, *H*-3'), 4.36 – 4.15 (m, 4H, *H*-4', *H*-5', C*H*CH_3), 3.12 (q, *J* = 7.3 Hz, 12H, CH_3C*H*$_2NH^+$), 1.44 (dd, *J* = 7.1, 1.0 Hz, 1.5H, CHC*H*$_3$), 1.28 (dd, *J* = 7.1, 0.9 Hz, 1.5H, CHC*H*$_3$), 1.24 (t, *J* = 7.3 Hz, 18H, C*H*$_3$$CH_2NH^+$). ^{31}P NMR (202 MHz, CD_3OD) δ -7.21 (d, *J* = 18.1 Hz, 0.5P), -8.07 (d, *J* = 18.9 Hz, 0.5P), -11.70 (d, *J* = 20.7 Hz, 0.5P), -11.73 (d, *J* = 19.9 Hz, 0.5P), -24.16 – (-24.58) (m, 1P). ^{13}C NMR (126 MHz, CD_3OD) δ 174.9 (d, J_{CP} = 6.1 Hz, *C*=O), 174.7 (d, J_{CP} = 6.5 Hz, *C*=O), 157.21 (*C*-6), 157.19 (*C*-6), 153.75 (*C*-2), 150.91 (*C*-4), 150.86 (*C*-4), 148.7 (d, J_{CP} = 7.3 Hz, *C*-Ar *ipso* OP), 148.2 (d, J_{CP} = 7.1 Hz, *C*-Ar *ipso* OP), 141.12 (*C*-8), 141.09 (*C*-8), 137.25 (*C*-Ar), 137.24 (*C*-Ar), 136.15 (*C*-Ar), 136.15 (*C*-Ar), 129.46 (*C*H-Ar), 129.45 (*C*H-Ar), 129.42 (*C*H-Ar), 129.11 (*C*H-Ar), 129.07 (*C*H-Ar), 129.06 (*C*H-Ar), 128.57 (*C*H-Ar), 128.53 (*C*H-Ar), 128.12 (d, J_{CP} = 6.4 Hz, *C*H-Ar), 128.08 (d, J_{CP} = 6.5 Hz, *C*H-Ar), 127.63 (*C*H-Ar), 127.58 (*C*H-Ar), 127.35 (*C*H-Ar), 127.29 (*C*H-Ar), 126.60 (d, J_{CP} = 1.8 Hz, *C*-Ar), 126.58 (d, J_{CP} = 1.9 Hz, *C*-Ar), 125.61 (*C*H-Ar), 125.54 (*C*H-Ar), 123.54 (*C*H-Ar), 120.17 (*C*-5), 120.12 (*C*-5), 116.50 (d, J_{CP} = 3.0 Hz, *C*H-Ar), 116.46 (d, J_{CP} = 3.4 Hz, *C*H-Ar), 88.93 (*C*-1'), 88.84 (*C*-1'), 85.60 (d, J_{CP} = 8.9 Hz, *C*-4'),

85.55 (d, J_{CP} = 9.0 Hz, *C*-4'), 76.08 (*C*-2'), 72.15 (*C*-3'), 71.97 (*C*-3'), 67.68 (O*C*H_2Ph), 67.65 (O*C*H_2Ph), 66.81 (d, J_{CP} = 6.2 Hz, *C*-5'), 66.76 (d, J_{CP} = 6.9 Hz, *C*-5'), 52.02 (d, J_{CP} = 2.7 Hz, *C*HCH_3), 51.66 (d, J_{CP} = 1.5 Hz, *C*HCH_3), 47.43 (CH_3*C*H_2NH^+), 20.85 (d, J_{CP} = 6.6 Hz, CH*C*H_3), 20.80 (d, J_{CP} = 6.4 Hz, CH*C*H_3), 9.10 (*C*$H_3CH_2NH^+$). HRMS-ESI (*m/z*): calcd for $C_{30}H_{34}N_6O_{14}P_3$ [M-$(Et_3N)_2$+H]$^+$ 795.1346, found 795.1347. HPLC Reversed-phase eluting with TEAB buffer/CH_3CN from 10/90 to 100/0 in 30 minutes, F = 1 mL/min, λ = 254 nm, t_R 12.832 min (fast eluting isomer) and 12.936 min (slow eluting isomer), purity 90%. ^{31}P-NMR purity 99%.

Uridine diphosphate di-triethylammonium salt

UDP disodium salt **4** (1 eq, 0.22 mmol, 100 mg) was dissolved in deionised water (5 mL), and the solution was passed through a Dowex 50W-X8 (H^+) column (10 x 55 mm). The pH of the eluent was neutralized with triethylamine (2 eq, 0.44 mmol, 61 µL). The mixture was evaporated and freeze-dried to afford the desired di-triethylammonium salt as a white solid (quant.). The compound was used without any further purification in the following step. ^{1}H NMR (500 MHz, CD_3OD) δ 8.10 – 8.02 (m, 1H, *H*-6), 5.98 – 5.92 (m, 1H, *H*-1'), 5.85 – 5.79 (m, 1H, *H*-5), 4.38 – 4.31 (m, 1H, *H*-2'), 4.30 – 4.17 (m, 3H, *H*-4', *H*-5'), 4.14 – 4.08 (m, 1H, *H*-3'), 3.15 (q, *J* = 7.2 Hz, 12H, CH_3C*H*$_2NH^+$), 1.30 (t, *J* = 6.6 Hz, 18H, C*H*$_3CH_2NH^+$). ^{31}P NMR (202 MHz, CD_3OD) *δ* -9.95 (d, *J* = 20.4 Hz, 1P), -11.14 (d, *J* = 20.4 Hz, 1P).

Benzyl ((*S*)-((((((2*R*,3*S*,4*R*,5*R*)-5-(2,4-dioxo-3,4-dihydropyrimidin-1(2*H*)-yl)-3,4-dihydroxytetrahydro furan-2-yl)methoxy)(hydroxy)phosphoryl)oxy)(hydroxy)phosphoryl)oxy)(phenoxy)phosphoryl)-*L*-alaninate tri-triethylammonium salt (5a)

Prepared according general procedure starting from uridine diphosphate di-triethylammonium salt (0.18 mmol, 108 mg), pentafluorophenyl phosphorylating reagent **2a** (0.36 mmol, 180 mg) and diisopropylamine (0.36 mmol, 50 µL) in *N*,*N*-dimethylformamide (2.25 mL) to obtain after purification 141 mg of desired compound **5a** as a white solid (77% over 2 steps). ^{1}H NMR (500 MHz, CD_3OD) *δ* 8.04 (dd, *J* = 8.1, 6.0 Hz, 1H, *H*-6), 7.37 – 7.24 (m, 9H, Ar-*H*), 7.17 – 7.09 (m, 1H, Ar-*H*), 5.95 (dd, *J* = 12.5, 5.2 Hz, 1H, *H*-1'), 5.81 (dd, *J* = 8.1, 2.7 Hz, 1H, *H*-5), 5.13 – 5.06 (m, 2H, OC*H*$_2$Ph), 4.35 – 4.06 (m, 6H, *H*-2', *H*-3', *H*-4', *H*-5', C*H*CH_3), 2.85 (q, *J* = 7.3 Hz, 18H, CH_3C*H*$_2NH^+$), 1.45 (dd, *J* = 7.1, 1.0 Hz, 1.5H, CHC*H*$_3$), 1.34 (dd, *J* = 7.1, 0.8 Hz, 1H, CHC*H*$_3$), 1.16 (t, *J* = 7.3 Hz, 27H, C*H*$_3CH_2NH^+$). ^{31}P NMR (202 MHz, CD_3OD) *δ* -7.48 (d, *J* = 18.4 Hz, 0.5P), -8.13 (d, *J* = 19.2 Hz, 0.5P), -11.87 (d, *J* = 21.0 Hz, 0.5P), -11.89 (d, *J* = 20.3 Hz, 0.5P), -24.32 – (-24.73) (m, 1P). ^{13}C NMR (126 MHz, CD_3OD) *δ* 174.8 (d, J_{CP} = 6.1 Hz, *C*=O), 174.7 (d, J_{CP} = 7.24 Hz, *C*=O), 166.22 (*C*=O), 166.19 (*C*=O), 152.7 (*C*=O), 152.6 (*C*=O), 152.41 (d, J_{CP} = 7.2 Hz, *C*-Ar *ipso* OP), 152.36 (d, J_{CP} = 7.1 Hz, *C*-Ar *ipso* OP), 142.8 (*C*-6), 137.43 (*C*-Ar), 137.41 (*C*-Ar), 130.6 (*C*H-Ar), 130.52 (*C*H-Ar), 130.51 (*C*H-Ar), 129.53 (*C*H-Ar), 129.52 (*C*H-Ar), 129.21 (*C*H-Ar), 129.19 (*C*H-Ar), 129.18 (*C*H-Ar), 129.16 (*C*H-Ar), 125.85 (*C*H-Ar), 125.84 (*C*H-Ar), 125.78 (*C*H-Ar), 125.77 (*C*H-Ar), 122.0 (d, J_{CP}

= 4.8 Hz, *C*H-Ar), 121.9 (d, J_{CP} = 5.0 Hz, *C*H-Ar), 103.3 (*C*-5), 103.2 (*C*-5), 89.7 (*C*-1'), 89.6 (*C*-1'), 85.10 (d, J_{CP} = 7.9 Hz, *C*-4'), 85.03 (d, J_{CP} = 7.9 Hz, *C*-4'), 75.7 (*C*-2'), 75.6 (*C*-2'), 71.4 (*C*-3'), 71.2 (*C*-3'), 67.74 (O*C*H_2Ph), 67.68 (O*C*H_2Ph), 66.35 (d, J_{CP} = 5.7 Hz, *C*-5'), 66.3 (d, J_{CP} = 5.6 Hz, *C*-5'), 51.9 (d, J_{CP} = 2.5 Hz, *C*HCH_3), 51.6 (d, J_{CP} = 1.9 Hz, *C*HCH_3), 47.2 (CH_3*C*H_2NH^+), 20.80 (d, J_{CP} = 5.3 Hz, CH*C*H_3), 20.76 (d, J_{CP} = 6.3 Hz, CH*C*H_3), 10.2 (*C*$H_3CH_2NH^+$). HRMS-ESI (*m/z*): calcd for $C_{25}H_{31}N_3O_{16}P_3$ [M-($Et_3N)_3$+H]$^+$ 722.0917, found 722.0917. HPLC Reversed-phase eluting with TEAB buffer/CH_3CN from 10/90 to 100/0 in 30 minutes, F = 1 mL/min, λ = 270 nm, t_R 11.534 min (fast eluting isomer) and 11.62 min (slow eluting isomer), purity 96%.

Adenosine diphosphate di-triethylammonium salt

ADP disodium salt (1 eq, 0.11 mmol, 50 mg) was dissolved in deionised water (1 mL), and the solution was passed through a Dowex 50W-X8 (Et_3NH^+) column (10 x 80 mm). The mixture was evaporated to afford the desired di-triethylammonium salt as a glassy solid (64 mg, 93%). The compound was used without any further purification in the following step. ^{1}H NMR (500 MHz, CD_3OD) δ 8.56 (s, 1H, *H*-8), 8.20 (s, 1H, *H*-2), 6.09 (d, *J* = 5.6 Hz, *H*-1'), 4.68 (dd, *J* = 5.6, 5.1 Hz, 1H, *H*-2'), 4.54 – 4.48 (m, 1H, *H*-3'), 4.32 – 4.17 (m, 3H, *H*-4', *H*-5'), 3.13 (q, *J* = 7.3 Hz, 12H, CH_3*C*H_2NH^+), 1.28 (t, *J* = 7.3 Hz, 18H, *C*$H_3CH_2NH^+$). ^{31}P NMR (202 MHz, MeOD) δ -9.95 (d, *J* = 20.5 Hz, 1P), -10.96 (d, *J* = 20.2 Hz, 1P).

Isopropyl ((*S*)-(((((((2*R*,3*S*,4*R*,5*R*)-5-(6-amino-9*H*-purin-9-yl)-3,4-dihydroxytetrahydrofuran-2-yl)methoxy)(hydroxy)phosphoryl)oxy)(hydroxy)phosphoryl)oxy)(phenoxy)phosphoryl)-*L*-alaninate di-triethylammonium salt (3b, S_P/R_P 9:1**)**

Prepared according to general procedure **3** starting from ADP di-triethylammonium salt (0.10 mmol, 64 mg), S_P-**2b** (0.20 mmol, 91 mg) and diisopropylamine (0.20 mmol, 28 µL) in *N*,*N*-dimethylformamide (1.2 mL) to obtain after purification 68 mg of desired compound **3b** (S_P/R_P 9:1) as a colourless oil (68%). Only major S_p-isomer is described ^{1}H NMR (500 MHz, CD_3OD) δ 8.57 (s, 1H, *H*-8), 8.21 (s, 1H, *H*-2), 7.33 – 7.24 (m, 4H, Ar-*H*), 7.15 – 7.06 (m, 1H, Ar-*H*), 6.09 (d, *J* = 5.8 Hz, 1H, *H*-1'), 4.94 – 4.90 (m, 1H, OC*H*($CH_3)_2$), 4.69 (dd, *J* = 5.8, 5.0 Hz, 1H, *H*-2'), 4.47 (dd, *J* = 5.1, 3.2 Hz, 1H, *H*-3'), 4.28 – 4.20 (m, 3H, *H*-4', *H*-5'), 4.04 (dq, *J* = 9.6, 7.1 Hz, 1H, C*H*CH_3), 3.16 (q, *J* = 7.3 Hz, 12H, CH_3C*H*$_2NH^+$), 1.42 (dd, *J* = 7.1, 0.9 Hz, 3H, CHC*H*$_3$), 1.27 (t, *J* = 7.3 Hz, 18H, C*H*$_3CH_2NH^+$), 1.18 (dd, *J* = 6.3, 5.2 Hz, 6H, OCH(C*H*$_3)_2$). ^{31}P NMR (202 MHz, CD_3OD) δ -7.46 (d, *J* = 18.2 Hz, 1P), -11.74 (d, *J* = 20.3 Hz, 1P), -24.35 (t, *J* = 19.2 Hz, 1P). ^{13}C NMR (126 MHz, CD_3OD) δ 174.7 (d, J_{CP} = 6.7 Hz, *C*=O), 157.2 (*C*-2), 153.8 (*C*-6), 152.4 (d, J_{CP} = 7.2 Hz, *C*-Ar *ipso* OP), 150.9 (*C*-4), 141.1 (*C*-8), 130.52 (*C*H-Ar), 130.51 (*C*H-Ar), 125.79 (*C*H-Ar), 125.78 (*C*H-Ar), 121.9 (d, J_{CP} = 4.9 Hz, *C*H-Ar), 120.1 (*C*-5), 88.8 (*C*-1'), 85.6 (d, J_{CP} = 9.2 Hz, *C*-4'), 76.1 (*C*-2'), 72.2 (*C*-3'), 69.9 (O*C*H($CH_3)_2$), 66.8 (d, J_{CP} = 5.8 Hz, *C*-5'), 51.9 (d, J_{CP} = 2.8 Hz, *C*HCH_3), 47.4 (CH_3*C*H_2NH^+), 22.0 (OCH(*C*$H_3)_2$), 21.9 (OCH(*C*$H_3)_2$), 20.9 (d, J_{CP} = 6.1 Hz, CH*C*H_3), 9.1 (*C*$H_3CH_2NH^+$). HRMS-ESI (*m/z*):

calcd for $C_{22}H_{32}N_6O_{14}P_3$ [M-$(Et_3N)_2$+H]$^+$ 697.1189, found 697.1202. HPLC Reversed-phase eluting with TEAB buffer/CH_3CN from 10/90 to 100/0 in 30 minutes, F = 1 mL/min, λ = 264 nm, t_R 9.705 min, purity 98%.

Scheme S1. Conversion of the triethylammonium salt of prodrug **3a** to the ammonium salt form (**6a**)

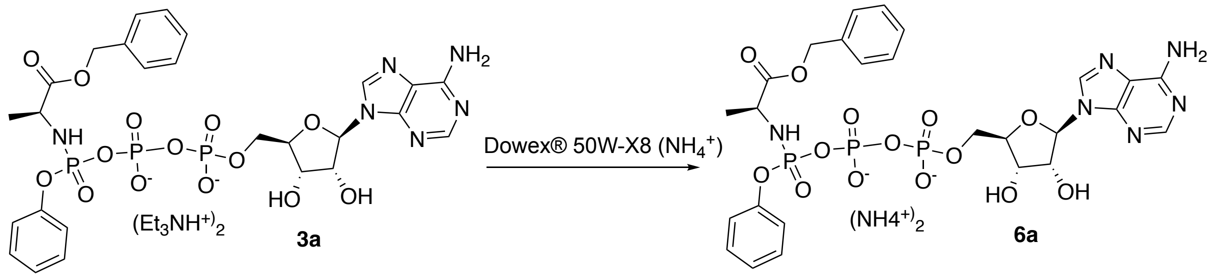

Benzyl-(((((((2*R*,3*S*,4*R*,5*R*)-5-(6-amino-9*H*-purin-9-yl)-3,4 dihydroxytetrahydrofuran-2-yl)methoxy)(hydroxy)phosphoryl)oxy)(hydroxy)phosphoryl)oxy)(phenoxy)phosphoryl)-*L*-alaninate di-ammonium salt (6a)

A 28-30% ammonia solution in water was passed through a Dowex® 50W-X8 (NH_4^+) column (10 x 70 mm). The column was rinsed with deionised water until pH 7 and then, a solution containing the prodrug **3a** as di-triethylammonium salt (0.06 mmol, 55 mg) in deionised water (1 mL) was applied. The eluate was evaporated to afford 41 mg of the desired di-ammonium salt **6a** as a glassy oil (94%). ^{1}H NMR (500 MHz, CD_3OD) δ 8.54 (d, *J* = 1.8 Hz, 1H, *H*-8), 8.19 (d, *J* = 1.5 Hz, 1H, *H*-2), 7.35 – 7.20 (m, 9H, Ar-*H*), 7.13 – 7.06 (m, 1H, Ar-*H*), 6.08 (dd, *J* = 5.8, 4.6 Hz, 1H, *H*-1’), 5.10 – 5.06 (m, 2H, OCH_2Ph), 4.69 (dd, *J* = 5.7, 5.3 Hz, 0.5H, *H*-2’), 4.64 (dd, *J* = 5.4, 5.4 Hz, 0.5H, *H*-2’), 4.51 – 4.45 (m, 1H, *H*-3’), 4.35 – 4.19 (m, 3H, *H*-4’, *H*-5’), 4.21 – 4.08 (m, 1H, C*H*CH_3), 1.45 (dd, *J* = 7.2, 0.9 Hz, 1.5H, CHCH_3), 1.33 (dd, *J* = 7.1, 0.9 Hz, 1.5H, CHCH_3). ^{31}P NMR (202 MHz, CD_3OD) δ -7.52 (d, *J* = 18.2 Hz, 0.5P), -8.17 (d, *J* = 19.1 Hz, 0.5P), -11.74 (d, *J* = 20.7 Hz, 0.5P), -11.77 (d, *J* = 20.0 Hz, 0.5P), -23.81 – (-25.02) (m, 1P). ^{13}C NMR (126 MHz, CD_3OD) δ 175.0 (d, J_{CP} = 5.5 Hz, *C*=O), 174.9 (d, J_{CP} = 6.0 Hz, *C*=O), 156.84 (*C*-2), 156.82 (*C*-2), 153.22 (*C*-6), 153.18 (*C*-6), 152.25 (d, J_{CP} = 7.1 Hz, *C*-Ar *ipso* OP), 152.18 (d, J_{CP} = 7.1 Hz, *C*-Ar *ipso* OP), 150.69 (*C*-4), 150.62 (*C*-4), 141.3 (*C*-8), 137.31 (*C*-Ar), 137.28 (*C*-Ar), 130.52 (*C*H-Ar), 130.49 (*C*H-Ar), 129.51 (*C*H-Ar), 129.50 (*C*H-Ar), 129.19 (*C*H-Ar), 129.18 (*C*H-Ar), 125.84 (*C*H-Ar), 125.84 (*C*H-Ar), 125.80 (*C*H-Ar), 125.79 (*C*H-Ar), 121.84 (d, J_{CP} = 4.9 Hz, *C*H-Ar), 121.75 (d, J_{CP} = 4.9 Hz, *C*H-Ar), 120.08 (*C*-5), 120.06 (*C*-5), 89.2 (*C*-1’), 89.1 (*C*-1’), 85.5 (d, J_{CP} = 9.6 Hz, *C*-4’), 85.4 (d, J_{CP} = 9.5 Hz, *C*-4’), 76.41 (*C*-2’), 76.39 (*C*-2’), 71.9 (*C*-3’), 71.7 (*C*-3’), 67.84 (O*C*H_2Ph), 67.78 (O*C*H_2Ph), 66.5 (d, J_{CP} = 5.9 Hz, *C*-5’), 66.4 (d, J_{CP} = 6.0 Hz, *C*-5’), 51.8 (d, J_{CP} = 2.5 Hz, *C*HCH_3), 51.6 (d, J_{CP} = 1.5 Hz, *C*HCH_3), 20.74 (d, J_{CP} = 4.8

Hz, CH*C*H$_3$), 20.69 (d, J_{CP} = 4.4 Hz, CH*C*H$_3$). HRMS-ESI (*m/z*): calcd for $C_{26}H_{32}N_6O_{14}P_3$ [M-NH$_3$)$_2$+H]$^+$ 745.1189, found 745.1197.

Synthesis of intermediates 9, 10 and Clofarabine prodrug 11a

(5-(6-amino-2-chloro-9*H*-purin-9-yl)-4-fluoro-3-hydroxytetrahydrofuran-2-yl)methyl 4-methyl benzenesulfonate (9).

To a solution of Clofarabine **7** (1 eq, 0.66 mmol, 200 mg) in pyridine (4 mL) under nitrogen atmosphere at 0°C was added tosyl chloride (1.5 eq, 0.99 mmol, 189 mg). After 10 min, the mixture was heated at 30°C for 3 hours. Then, few pieces of ice and deionised water were added at 0°C. The product was extracted with dichloromethane, washed with saturated bicarbonate aqueous solution and dried over $MgSO_4$. After concentration under reduced pressure, the crude was purified on Biotage Isolera silica (25 g cartridge, gradient of methanol in dichloromethane from 0% to 10%) to afford 157 mg of desired compound **9** as a white solid (52%). ^{1}H NMR (400 MHz, acetone-*d*6) *δ* 7.96 (d, *J* = 2.3 Hz, 1H, *H*-8), 7.83 – 7.75 (m, 2H, *H*-Ar), 7.44 – 7.34 (m, 2H, *H*-Ar), 7.07 (bs, 2H, NH$_2$), 6.40 (dd, *J* = 15.7, 4.3 Hz, 1H, *H*-1'), 5.36 (d, *J* = 4.9 Hz, 1H, OH), 5.34 (dd, *J* = 4.3, 3.4 Hz, 0.5H, *H*-2'), 5.21 (dd, *J* = 4.2, 3.5 Hz, 0.5H, *H*-2'), 4.75 – 4.63 (m, 1H, *H*-3'), 4.41 (d, *J* = 4.8 Hz, 1H, *H*-5'), 4.25 – 4.16 (m, 1H, *H*-4'), 2.41 (s, 3H, C*H*$_3$). ^{13}C NMR (101 MHz, acetone-*d*6) *δ* 157.9 (*C*-6), 154.8 (*C*-2), 151.5 (*C*-4), 146.1 (*C*-Ar), 141.1 (d, *J* = 4.8 Hz, *C*-8), 133.8 (*C*-Ar), 130.7 (*C*H-Ar), 128.8 (*C*H-Ar), 118.9 (*C*-5), 95.9 (d, *J* = 192.5 Hz, *C*-2'), 83.5 (d, *J* = 16.8 Hz, *C*-1'), 81.7 (d, *J* = 4.9 Hz, *C*-4'), 74.9 (d, *J* = 24.7 Hz, *C*-3'), 70.1 (d, *J* = 2.2 Hz, *C*-5'), 21.5 (*C*H$_3$). ^{19}F NMR (376 MHz, acetone-*d*6) *δ* -199.40. HRMS-ESI (*m/z*): calcd for $C_{17}H_{18}N_5O_5SClF$ [M+H]$^+$ 458.0701, found 458.0701.

(((2*R*,3*S*,4*R*,5*R*)-5-(6-amino-2-chloro-9*H*-purin-9-yl)-4-fluoro-3-hydroxytetrahydrofuran-2-yl)methyl diphosphate) di-triethylammonium salt (10)[1]

To a suspension of (5-(6-amino-2-chloro-9*H*-purin-9-yl)-4-fluoro-3-hydroxytetrahydrofuran-2-yl)methyl 4-methylbenzene sulfonate **9** (1 eq, 0.11 mmol, 50 mg) in acetonitrile under nitrogen atmosphere was added tris(tetrabutylammonium) hydrogen pyrophosphate (HPP) (2 eq, 0.22 mmol, 200 mg). The resulting mixture was stirred at room temperature for 3 days. After concentration under reduced pressure, the crude was solubilised in deionised water (1 mL) and the cloud solution was passed through a Dowex 50W-X8 (Et$_3$NH$^+$) column (10 x 80 mm). The mixture was evaporated to afford **10** as a colourless oil. The compound was used without any further purification in the following step. ^{31}P NMR (202 MHz, CD$_3$OD) *δ* -11.11 (d, *J* = 18.3 Hz, 1P), -12.19 (d, *J* = 18.3 Hz, 1P).

Benzyl ((*S*)-((((((2*R*,3*R*,4*S*,5*R*)-5-(6-amino-2-chloro-9*H*-purin-9-yl)-4-fluoro-3-hydroxytetrahydrofuran-2-

yl)methoxy)(hydroxy)phosphoryl)oxy)(hydroxy)phosphoryl)oxy)(phenoxy)phosphoryl)-*L*-alaninate tri-triethylammonium salt (11a).

Prepared according to general procedure **3** starting from (((2*R*,3*S*,4*R*,5*R*)-5-(6-amino-2-chloro-9*H*-purin-9-yl)-4-fluoro-3-hydroxytetrahydrofuran-2-yl)methyl diphosphate) di-triethylammonium salt **10** (1 eq, 0.11 mmol), pentafluorophenyl phosphorylating reagent **2a** (2 eq, 0.22 mmol, 110 mg) and diisopropylamine (2 eq, 0.22 mmol, 31 µL) in *N*,*N*-dimethylformamide (1.4 mL) to obtain after three RP chromatography purifications and two washing with diethyl ether 28 mg of desired compound **11a** as light yellow oil (23%).^{1}H NMR (500 MHz, CD_3OD) *δ* 8.22 (s, 0.5H, *H*-8), 8.21 (s, 0.5H, *H*-8), 7.22 – 7.16 (m, 9H, *H*-Ar),7.04-6.99 (m,1H, *H*-Ar) 6.40 (dd, *J* = 4.2 Hz, J_{HF} = 15.5 Hz, 0.5H, H-1'), 6.40 (dd, *J* = 4.3 Hz, J_{HF} = 15.3 Hz, 0.5H, H-1'), 5.10 – 5.08 (m, 0.5H, H-2') 5.00 – 4.97 (m, 2.5H, H-2' and OCH_2Ph), 4.52 (ddd, *J* = 3.5, 4.8 Hz, J_{HF} = 20.0 Hz, 1H, *H*-3'), 4.26 – 4.26 (m, 2H, *H*-5'), 4.08 – 4.02 (m, 2H, *H*-4', C*H*CH_3), 3.02 (q, *J* = 7.3 Hz, 18H, $CH_3CH_2NH^+$), 1.35 (d, *J* = 7.1 Hz, 1.5H, $CHCH_3$), 1.24 (d, *J* = 7.1 Hz, 1H, $CHCH_3$), 1.17 (t, *J* = 7.3 Hz, 27H, $CH_3CH_2NH^+$). ^{31}P NMR (202 MHz, CD_3OD) *δ* -7.55 (d, *J* = 18.3 Hz, 0.5P), -8.15 (d, *J* = 18.9 Hz, 0.5P), -11.65 (d, *J* = 20.2, 5.4 Hz, 1P), -24.47 (d, *J* = 20.2 Hz, 1P). ^{19}F NMR (471 MHz, CD_3OD) *δ* -199.75 (0.5F), -199.78 (0.5F). ^{13}C NMR (126 MHz, CD_3OD) *δ* 173.53 (d, J_{CP} = 6.3 Hz, *C*=O), 173.41 (d, J_{CP} = 7.1 Hz, *C*=O), 156.68 (*C*-6), 154.10 (*C*-2), 150.99 (d, J_{CP} = 7.0 Hz, *C*-Ar *ipso* OP), 150.94 (d, J_{CP} = 6.9 Hz, *C*-Ar *ipso* OP), 150.24 (*C*-4), 140.58 (*C*H-8) 135.99 (*C*-Ar), 129.16 (*CH*-Ar), 129.11 (*CH*-Ar), 128.12 (*CH*-Ar), 127.77 (*CH*-Ar), 124.46 (*CH*-Ar), 124.38 (*CH*-Ar), 120.55 (d, J_{CP} = 4.9 Hz, *CH*-Ar), 120.53 (d, J_{CP} = 5.0 Hz, *CH*-Ar), 117.11 (*C*-5), 94.9 (d, J_{CF} = 316 Hz, *C*-2'), 82.66 (d, J_{CP} = 5.0 Hz, *C*-4'), 82.62 (d, J_{CP} = 5.0 Hz, *C*-4'), 82.50 (d, J_{CF} = 16.3 Hz, *C*-1'), 74.7 (d, J_{CF} = 24.4 Hz, *C*-3'), 74.5 (d, J_{CF} = 24.3 Hz, *C*-3'), 66.36 (OCH_2Ph), 66.31 (OCH_2Ph), 64.4 (d, J_{CP} = 4.5 Hz, *C*-5'), 50.5 (d, J_{CP} = 2.6 Hz, C*H*CH_3), 50.21 (d, J_{CP} = 1.8 Hz, C*H*CH_3), 47.5 ($CH_3CH_2NH^+$), 20.79 (d, J_{CP} = 5.5 Hz, $CHCH_3$), 20.77 (d, J_{CP} = 6.4 Hz, $CHCH_3$), 7.8 ($CH_3CH_2NH^+$). HPLC Reversed-phase eluting with TEAB buffer/CH_3CN from 10/90 to 100/0 in 30 minutes, F = 1 mL/min, λ = 264 nm, t_R 14.17 min, purity 92%. HRMS-ESI (*m/z*): calcd for $C_{26}H_{30}N_6O_{13}FP_3Cl$ $[M-(Et_3N)_3+H]^+$ 781.0756, found 781.0776.

Synthesis of 5'-tosylate intermediate 15 (Scheme S1) and Gemcitabine prodrug 17a

Scheme S2. Synthesis of 5'- tosylate intermediate **15.**[a]

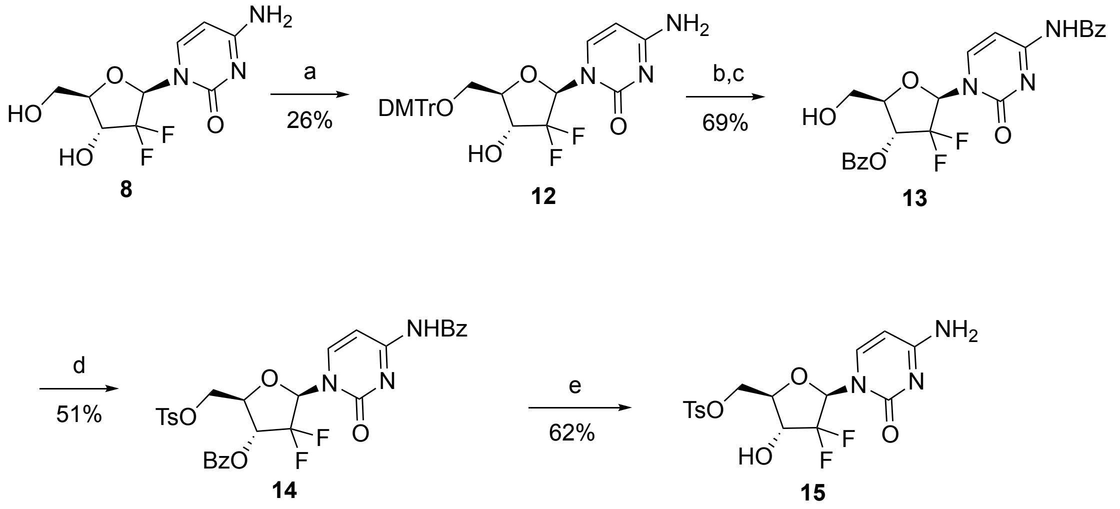

Reaction conditions: a) DMT-Cl (1.2 equiv), pyridine, rt, 16 h; b) Bz-Cl (3 equiv), pyridine, rt, 1 h 30; c) *p*TsOH.H_2O (1.5 equiv), DCM, rt, 3 h; d) Ts-Cl (32 equiv), Et_3N (16 equiv), pyridine, rt, 2 h; e) 7N NH_3 in MeOH, rt, 1 h.

4-amino-1-((2*S*,4*R*,5*R*)-5-((bis(4-methoxyphenyl)(phenyl)methoxy)methyl)-3,3-difluoro-4-hydroxytetrahydrofuran-2-yl)pyrimidin-2(1*H*)-one (12)[2]

To a solution of Gemcitabine **8** (1 eq, 2.28 mmol, 600 mg) in dry pyridine (25 mL) under nitrogen atmosphere at 0°C was added DMT-Cl (1.2 eq, 2.74 mmol, 928 mg). The resulting yellow solution was let to warm at room temperature overnight and stirred overnight. Then, the reaction was quenched with methanol (30 mL). After concentration under reduced pressure, the crude was purified on silica gel column (120 mL of silica, 20 x 3 cm column size) with a gradient of methanol in dichloromethane from 2% to 8% to afford 334 mg of desired compound as a white solid (26% yield). ^{1}H NMR (500 MHz, $CDCl_3$) δ 7.71 – 7.64 (m, 1H, *H*-6), 7.43 – 7.37 (m, 2H, *H*-Ar), 7.33 – 7.21 (m, 6H, *H*-Ar), 7.21 – 7.12 (m, 1H, *H*-Ar), 6.84 – 6.77 (m, 4H, *H*-Ar), 6.42 – 6.34 (m, 1H, *H*-1'), 5.52 (d, *J* = 7.5 Hz, 1H, *H*-5), 4.46 – 4.33 (m, 1H, *H*-3'), 4.11 – 4.01 (m, 1H, *H*-4'), 3.73 (s, 6H, $(OCH_3)_2$), 3.56 – 3.48 (m, 1H, *H*-5'), 3.47 – 3.36 (m, 1H, *H*-5'). ^{19}F NMR (471 MHz, $CDCl_3$) δ -117.23 (d, *J* = 241.0 Hz), -119.58 – (-121.08) (brs). MS *m/z* [M+H$^+$] 566.

(2*R*,3*R*,5*S*)-5-(4-benzamido-2-oxopyrimidin-1(2*H*)-yl)-4,4-difluoro-2-(hydroxymethyl)tetrahydro furan-3-yl benzoate (13).[20]

To a solution of 4-amino-1-((2*S*,4*R*,5*R*)-5-((bis(4-methoxyphenyl)(phenyl)methoxy)methyl)-3,3-difluoro-4-hydroxytetrahydrofuran-2-yl)pyrimidin-2(1*H*)-one **12** (1 eq, 1.01 mmol, 570 mg) in dry pyridine (5 mL) under nitrogen atmosphere at 0°C was added benzoyl chloride dropwise (3 eq, 3.03 mmol, 352 µL). The resulting solution was let to warm at room temperature and stirred for 1h30. The crude mixture was concentrated under reduced pressure to 1/3 volume. Then, Et_2O (20 mL) was added and the white precipitate was filtered off. The filtrate was washed with 5% $NaHCO_3$ aqueous solution (2 x 40 mL), dried over $MgSO_4$ and concentrated under reduced pressure to obtain a white solid.[20]

This crude solid was then solubilised in dry dichloromethane (10 mL) under nitrogen atmosphere before the addition of *p*TsOH.H_2O (1.5 eq, 1.51 mmol, 287 mg). The resulting orange solution was stirred at room temperature for 3 h. After concentration under reduced pressure, the crude was purified on silica gel column (120 mL of silica, 20 x 3 cm column size) with a gradient of methanol in dichloromethane from 0% to 4% to afford 327 mg of compound **13** as a white solid (69% yield). ^1H NMR (400 MHz, DMSO-*d6*) δ 11.45 (s, 1H, NH), 8.32 (d, *J* = 7.7 Hz, 1H, *H*-6), 8.13 – 7.96 (m, 4H, *H*-Ar), 7.79 – 7.71 (m, 1H, *H*-Ar), 7.67 – 7.49 (m, 5H, *H*-Ar), 7.47 (d, *J* = 7.6 Hz, 1H, *H*-5), 6.44 (dd, *J* = 8.4 Hz, 1H, *H*-1’), 5.70 – 5.59 (m, 1H, *H*-3’), 5.38 (dd, *J* = 5.8 Hz, 1H, OH), 4.50 (dt, *J* = 7.0, 3.4 Hz, 1H, *H*-4’), 3.92 – 3.82 (m, 1H, *H*-5’), 3.81 – 3.69 (m, 1H, *H*-5’). ^{19}F NMR (471 MHz, DMSO-*d6*) δ -112.87 (d, *J* = 239.3 Hz), -114.06 – (-116.71) (brs). MS *m/z* [M+H$^+$] 472.

(2*R*,3*R*,5*R*)-5-(4-benzamido-2-oxopyrimidin-1(2*H*)-yl)-4,4-difluoro-2-((tosyloxy)methyl)tetrahydro furan -3-yl benzoate (14)[3]

To a solution of (2*R*,3*R*,5*S*)-5-(4-benzamido-2-oxopyrimidin-1(2*H*)-yl)-4,4-difluoro-2-(hydroxymethyl)tetrahydrofuran-3-yl benzoate **13** (1 eq, 0.11 mmol, 50 mg) in dry pyridine (2 mL) under nitrogen atmosphere at 0°C was added tosyl chloride (32 eq, 3.52 mmol, 671 mg) and triethylamine (16 eq, 1.76 mmol, 245 µL) successively. The resulting solution was stirred for 2h at room temperature. After concentration under reduced pressure, the crude was purified on silica gel column (20 mL of silica, 10 x 2 cm column size) with a gradient of acetone in dichloromethane from 0% to 25% to afford 35 mg of compound **14** as a yellow solid (51% yield). ^1H NMR (500 MHz, $CDCl_3$) δ 8.07 – 8.02 (m, 2H, *H*-Ar), 7.90 (d, *J* = 7.7 Hz, 1H, *H*-6), 7.87 – 7.80 (m, 2H, *H*-Ar), 7.77 (d, *J* = 7.6 Hz, 1H, *H*-5), 7.68 – 7.57 (m, 3H, *H*-Ar), 7.57 – 7.45 (m, 4H, *H*-Ar), 7.42 – 7.36 (m, 2H, *H*-Ar), 6.61 – 6.45 (m, 1H, *H*-1’), 5.53 – 5.37 (m, 1H, *H*-3’), 4.63 – 4.53 (m, 1H, *H*-4’), 4.50 – 4.41 (m, 2H, *H*-5’), 2.46 (s, 3H, CH_3). ^{19}F NMR (471 MHz, $CDCl_3$) δ -115.25 (d, *J* = 246.6 Hz), -118.37 – (-121.39) (brs). MS *m/z* [M+H$^+$] 626.

((2*R*,3*R*,5*R*)-5-(4-amino-2-oxopyrimidin-1(2*H*)-yl)-4,4-difluoro-3-hydroxytetrahydrofuran-2-yl)methyl 4-methylbenzenesulfonate (15).[3]

(2*R*,3*R*,5*R*)-5-(4-benzamido-2-oxopyrimidin-1(2*H*)-yl)-4,4-difluoro-2-((tosyloxy)methyl)tetrahydro furan-3-yl benzoate **14** (1 eq, 0.17 mmol, 110 mg) was solubilised and stirred in a solution of 7N ammonia in MeOH (5 mL) under nitrogen atmosphere for 1 h at room temperature. After concentration under reduced pressure, the crude was purified on silica gel column (20 mL of silica, 11 x 2 cm column size) with a gradient of methanol in dichloromethane from 2% to 8% to afford 44 mg of compound **15** as a yellow solid (62% yield). ^{1}H NMR (400 MHz, CD_3OD) δ 7.86 – 7.77 (m, 2H, *H*-Ar), 7.48 – 7.39 (m, 3H, *H*-Ar, *H*-6), 6.15 (t, *J* = 8.3 Hz, 1H, *H*-1'), 5.88 (d, *J* = 7.6 Hz, 1H, *H*-5), 4.43 (dd, *J* = 11.7, 2.5 Hz, 1H, *H*-5'), 4.34 (dd, *J* = 11.7, 4.5 Hz, 1H, *H*-5'), 4.23 – 4.09 (m, 1H, *H*-3'), 4.08 – 3.99 (m, 1H, *H*-4'), 2.45 (s, 3H, CH_3). ^{13}C NMR (101 MHz, CD_3OD) δ 167.6 (*C*-4), 157.6 (*C*-2), 146.9 (*C*-Ar), 142.3 (*C*-6), 134.0 (*C*-Ar), 131.2 (*C*-Ar), 129.1 (*C*-Ar), 123.3 (t, *J* = 258.9 Hz, *C*-2'), 96.6 (*C*-5), 86.3 (*C*-1'), 79.3 (d, *J* = 4.9 Hz, *C*-4'), 72.5 – 69.8 (m, *C*-3'), 69.0 (*C*-5'), 21.6 (*C*H_3). ^{19}F NMR (376 MHz, CD_3OD) δ -118.50 (d, *J* = 238.6 Hz), -119.05 – (-120.60) (brs). MS *m/z* [M+H^{+}] 418.

((2*R*,3*R*,5*R*)-5-(4-amino-2-oxopyrimidin-1(2*H*)-yl)-4,4-difluoro-3-hydroxytetrahydrofuran-2-yl)methyl trihydrogen diphosphate di-triethylammonium salt (16).[4]

To a suspension of ((2*R*,3*R*,5*R*)-5-(4-amino-2-oxopyrimidin-1(2*H*)-yl)-4,4-difluoro-3-hydroxytetrahydrofuran-2-yl)methyl 4-methylbenzenesulfonate **15** (1 eq, 0.10 mmol, 42 mg) in acetonitrile under nitrogen atmosphere was added tris(tetrabutylammonium) hydrogen pyrophosphate (HPP) (2 eq, 0.20 mmol, 180 mg). The resulting mixture was stirred at room temperature for 2 days. After concentration under reduced pressure, the crude was solubilised in deionised water (1 mL) and the cloudy solution was passed through a Dowex 50W-X8 (Et_3NH^+) column (10 x 80 mm). The mixture was evaporated to afford **16** as a colourless oil. The compound was used without any further purification in the following step. ^{31}P NMR (202 MHz, MeOD) δ -10.82 (d, *J* = 18.5 Hz, 1P), -12.15 (d, *J* = 18.5 Hz, 1P).

Benzyl ((((((((2*R*,3*R*,5*R*)-5-(4-amino-2-oxopyrimidin-1(2*H*)-yl)-4,4-difluoro-3-hydroxytetrahydrofuran-2-yl)methoxy)(hydroxy)phosphoryl)oxy)(hydroxy)phosphoryl)oxy)(phenoxy)phosphoryl)-L-alaninate tetra-triethylammonium salt (17a).

Prepared according to general procedure **3** starting from ((2*R*,3*R*,5*R*)-5-(4-amino-2-oxopyrimidin-1(2*H*)-yl)-4,4-difluoro-3-hydroxytetrahydrofuran-2-yl)methyl trihydrogen diphosphate di-triethylammonium salt di-triethylammonium salt **16** (1 eq, 0.20 mmol), (*S*)-2-[-(*S*)-(2,3,4,5,6-Pentafluoro-phenoxy)-phenoxy-phosphorylamino] Propionic Acid Benzyl Ester **2a** (2 eq, 0.40 mmol, 200 mg) and diisopropylamine (2 eq, 0.40 mmol, 56 µL) in *N*,*N*-dimethylformamide (2.4 mL) to obtain after several purifications and washing with diethyl ether 29 mg of desired compound **17a** as glassy

yellow oil (13 %).^{1}H NMR (500 MHz, CD_3OD) δ 7.92 (d, *J* = 2.1 Hz, 0.5H, *H*-6), 7.90 (d, *J* = 2.1 Hz, 0.5H, *H*-6), 7.36 – 7.25 (m, 10H, *H*-Ar), 6.27 – 6.18 (m, 1H, *H*-1'), 6.00 (d, *J* = 7.6 Hz, 0.5H, *H*-5), 5.99 (d, *J* = 7.6 Hz, 0.5H, *H*-5), 5.13 – 5.06 (m, 2H, OCH_2Ph), 4.49 – 4.25 (m, 3H, *H*-5', *H*-3'), 4.20 – 4.08 (m, 1H, C*H*CH_3), 4.04 – 3.91 (m, 1H, *H*-4'), 3.17 (q, *J* = 7.2 Hz, 24H, $CH_3CH_2NH^+$), 1.44 (dd, *J* = 7.1, 1.0 Hz, 1.5H, $CHCH_3$), 1.34 (dd, *J* = 7.1, 0.9 Hz, 1.5H, $CHCH_3$), 1.29 (t, *J* = 7.1 Hz, 36H, $CH_3CH_2NH^+$). ^{31}P NMR (202 MHz, CD_3OD) δ -7.51 (d, *J* = 18.4 Hz, 0.5P), -8.13 (d, *J* = 18.9 Hz, 0.5P), -11.93 (d, *J* = 20.5 Hz, 1P), -24.32 – (-25.01) (m, 1P).^{13}C NMR (125 MHz, CD_3OD) δ 173.58 (*C*=O), 173.52 (*C*=O), 166.35 (*C*-4), 157.63 (*C*-2), 150.93 (d, J_{CP} = 9.8 Hz, *C*-Ar *ipso* OP), 150.85 (d, J_{CP} =9.8 Hz, *C*-Ar *ipso* OP), 141.03 (*C*-6), 135.95 (*C*-Ar), 135.92 (*C*-Ar), 129.61, 129.11, 128.13, 128.11, 127.82, 127.81, 127.79 (*CH*-Ar),123.72 (d, J_{CF} = 275 Hz, *C*-2'), 124.48, 120.41 (d, J_{CP}= 4.5 Hz CH-Ar), 124.33 (CH-Ar), 95.4 (*C*-5), 84.69 (broad signal *C*-1'), 79.42 (*C*-4'), 68.53, 68.37 (t, J_{CF} = 24.6 Hz, *C*-3'), 66.35 (CH_2Ph), 62.65 (d, J_{CP} = 5.4 Hz, *C*-5'), 62.58 (d, J_{CP} = 5.4 Hz, *C*-5'), 66.46 (CH_2Ph), 50.22 (*C*HCH_3) 50.19 (*C*HCH_3), 46.22 (NCH_2CH_3) 19.31 (d, J_{CP} = 5.7 Hz, $CHCH_3$), 19.28 (d, J_{CP} = 5.7 Hz, $CHCH_3$), 10.16 (NCH_2CH_3).^{19}F NMR (471 MHz, CD_3OD) δ -119.30 (d, *J* = 12.9 Hz), -119.81 (d, *J* = 12.4 Hz), -120.02 (d, *J* = 12.3 Hz), -120.54 (d, *J* = 13.3 Hz). HRMS-ESI (*m/z*): calcd for $C_{25}H_{30}N_4O_{14}P_3F_2$ $[M-(Et_3N)_4+H]^+$ 741.0939, found 741.0942. HPLC Reversed-phase eluting with TEAB buffer/CH_3CN from 10/90 to 100/0 in 30 minutes, F = 1 mL/min, λ = 264 nm, t_R 12.067 min (fast eluting isomer). t_R 12.161 min (slow eluting isomer), purity 89%.

Stability Studies and Biological Evaluation Procedures

Chemical stability in phosphate buffer at pH 6.5 and 7.4

Prodrugs **3a**, **5a**, **11a** and **17a** were incubated in phosphate buffer (100 mM, pH 6.5 and pH 7.4) at 37°C in a water bath. The incubation mixture was prepared by dissolving 500 µL of stock solution of prodrugs (1 mg/mL in phosphate buffer) in 500 µL of pre-heated phosphate buffer (V_{final} = 1 mL). Aliquots of 100 µL were withdrawn at appropriate intervals and analysed by HPLC.

HPLC analysis of prodrugs was performed on Agilent Technologies 1260 Infinity apparatus with Agilent Technologies Zorbax pursuit 3 C18, 150x4.6 mm column with a 10-100% MeCN gradient in triethylammonium bicarbonate (TEAB) buffer (0.1 M, pH 7.4) over 30 min at a flow rate of 1.0 mL/min. The UV detector was operated at either 264 or 270 nm.

Chemical stability in rat serum

Prodrugs **3a**, **11a** and **17a** were incubated in rat serum at 37°C in a water bath. The incubation mixture was prepared by dissolving 40 µL of stock solution of prodrugs (1 mg/mL in pre-heated 100 mM phosphate buffer at pH 7.4) in 260 µL of pre-heated 100 mM phosphate buffer at pH 7.4 and 120 µL of rat serum. Aliquots of 25 µL were withdrawn at appropriate intervals, mixed with methanol (25 µL), and centrifuged (5 min at 6,000 rpm). Aliquots of 25 µL of the supernatant liquid were analysed by HPLC.

HPLC analysis of prodrugs was performed on Agilent Technologies 1260 Infinity apparatus with Agilent Technologies Zorbax pursuit 3 C18, 150x4.6 mm column with a 10-100% MeCN gradient in triethylammonium bicarbonate (TEAB) buffer (0.1 M, pH 7.4) over 30 min at a flow rate of 1.0 mL/min. The auto-sampler was set at 5°C. The UV detector was operated at 254 nm.

MTS Cell Viability Assay

The assay was contracted and carried-out by WuXi AppTec (Shanghai) Co., Ltd. The tumor cell lines CCRF-CEM, HL-60, KG-1, MOLT-4, K562, RPMI-8226, RL, HepG2, MCF-7, HT29, OVCAR3, were seeded at cell densities of 0.5 to 100 Å~ 103 cells/well in a 96-well plate the day before drug incubation. Then the plates were incubated for 72 h with the different concentrations of **11a**. After the incubation period, 50 µL of MTS was added and the tumor cells were incubated for 4 h at 37 °C. The data were read and collected by a Spectra Max 340 absorbance microplate reader. The compounds were tested in duplicate with 9 serial concentrations (3.16-fold titrations with 198 µM as the highest concentration), and the data were analysed by XLfit software.

^{1}H- and ^{31}P-NMR spectra of reagents 2a-d

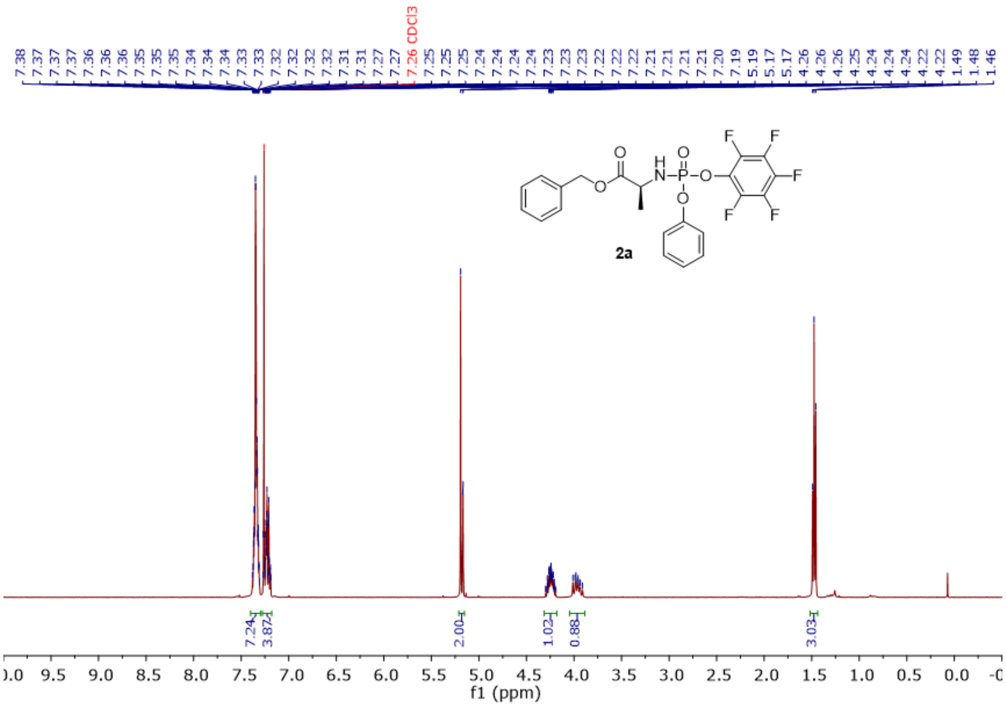

^{1}H-NMR (500 MHz, $CDCl_3$)

2a

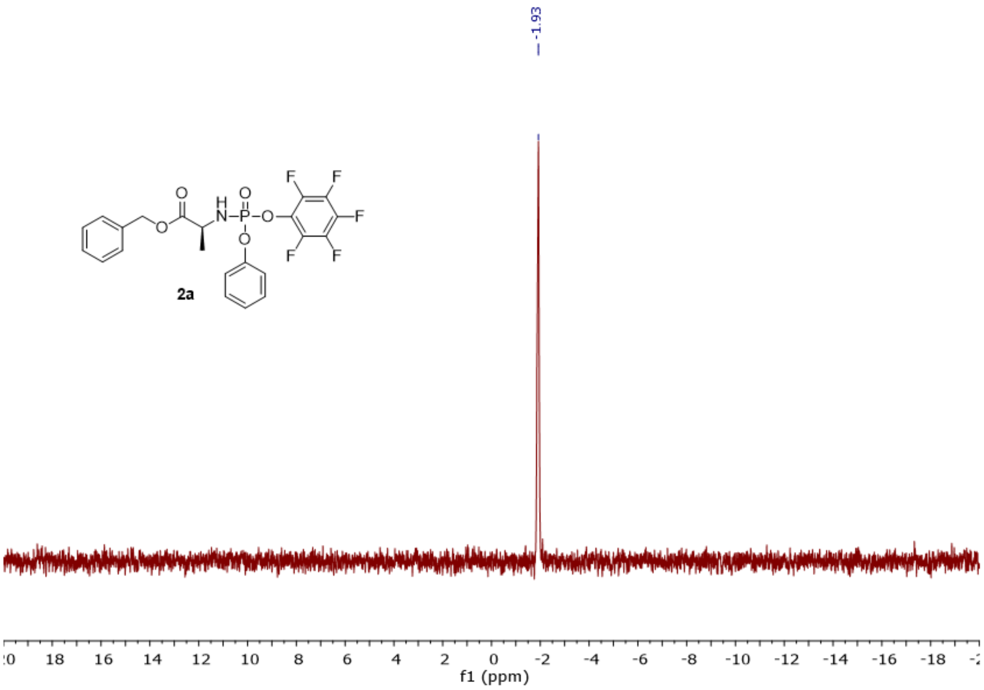

^{31}P-NMR (202 MHz, $CDCl_3$)

2a

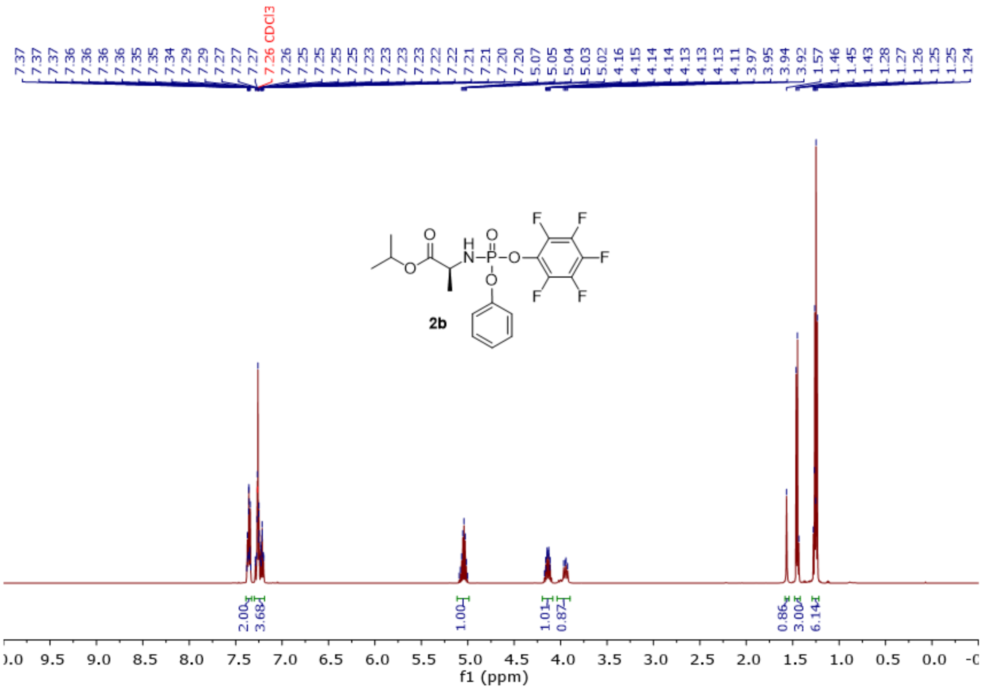

^{1}H-NMR (500 MHz, $CDCl_3$)

2b

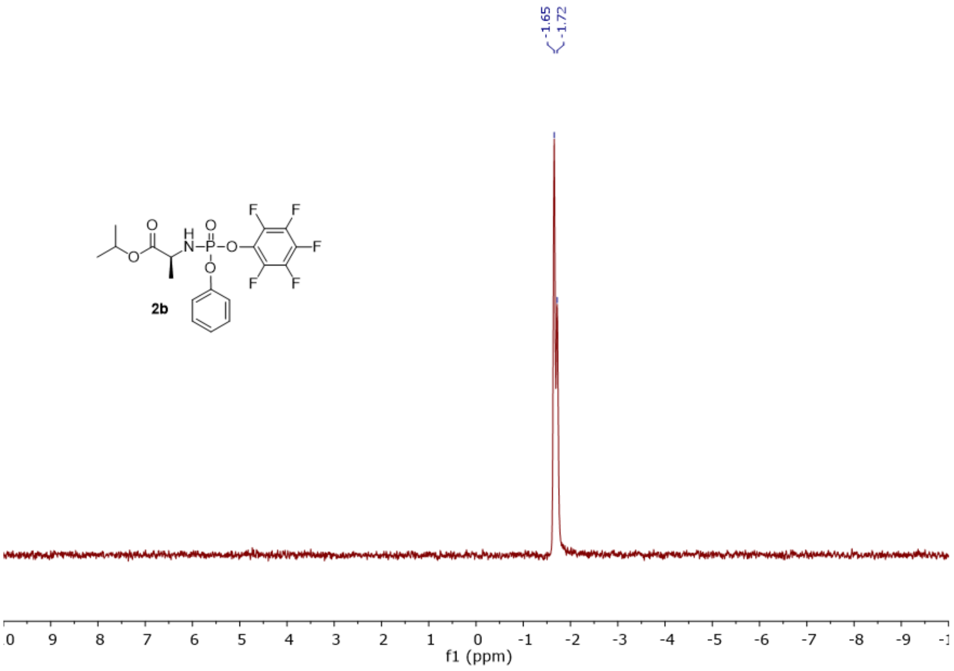

^{31}P-NMR (202 MHz, $CDCl_3$)

2b

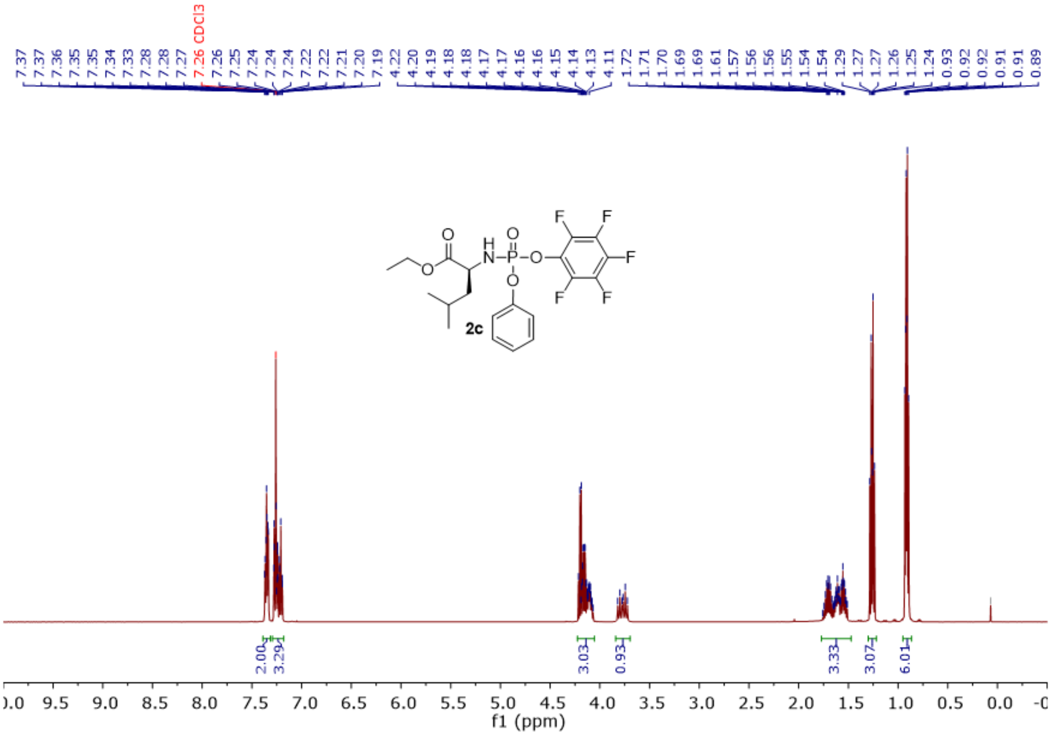

^{1}H-NMR (500 MHz, $CDCl_3$)

2c

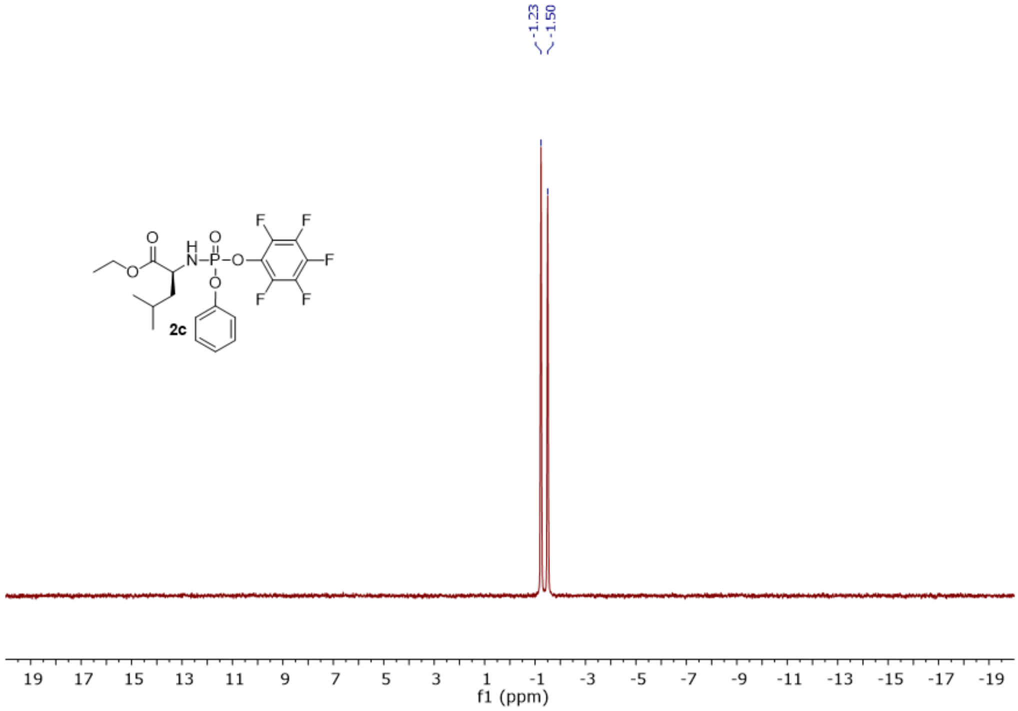

^{31}P-NMR (202 MHz, $CDCl_3$)

2c

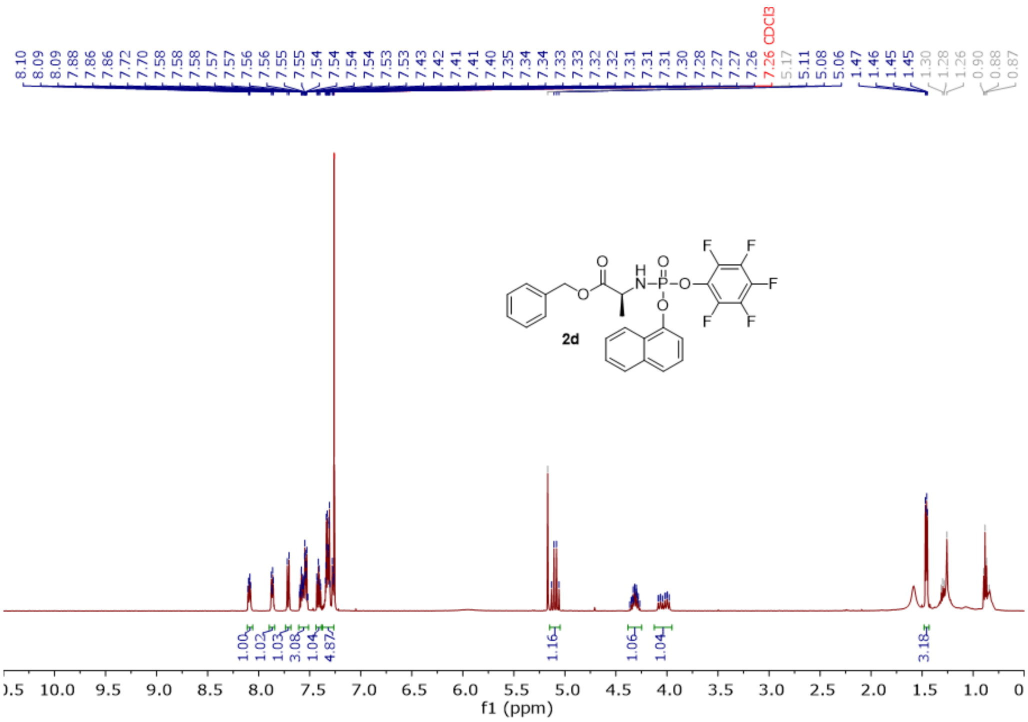

^{1}H-NMR (500 MHz, CDCl3)

2d

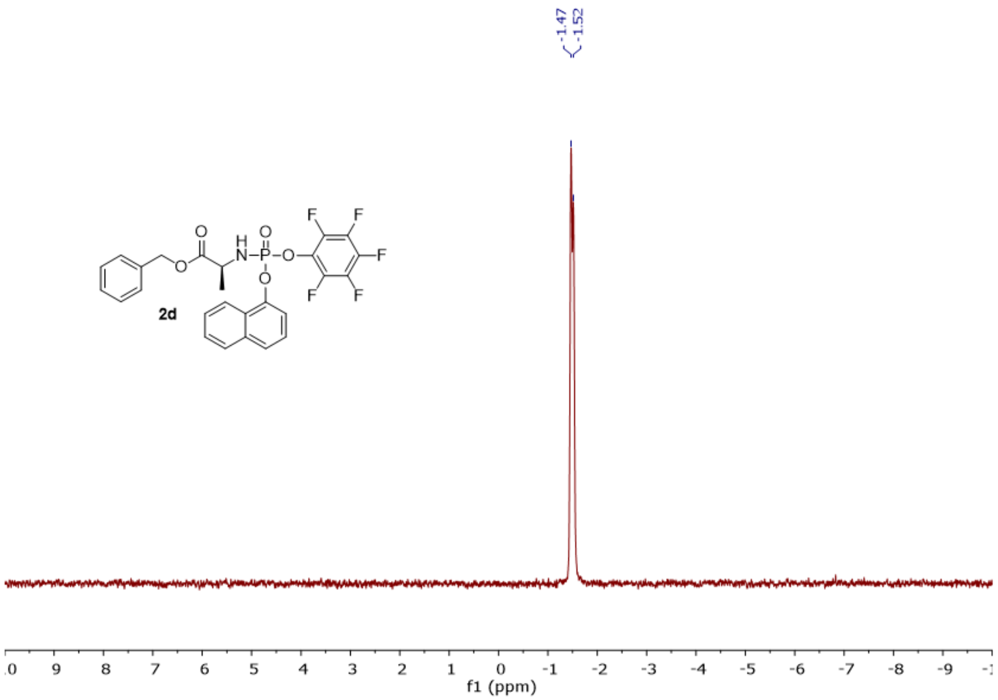

^{31}P-NMR (202 MHz, $CDCl_3$)

2d

^{1}H-, ^{31}P-, ^{13}C-NMR spectra, HRMS spectra and HPLC chromatograms of prodrugs 3a-d, 5a, and 3b (S_p/R_p 9:1)

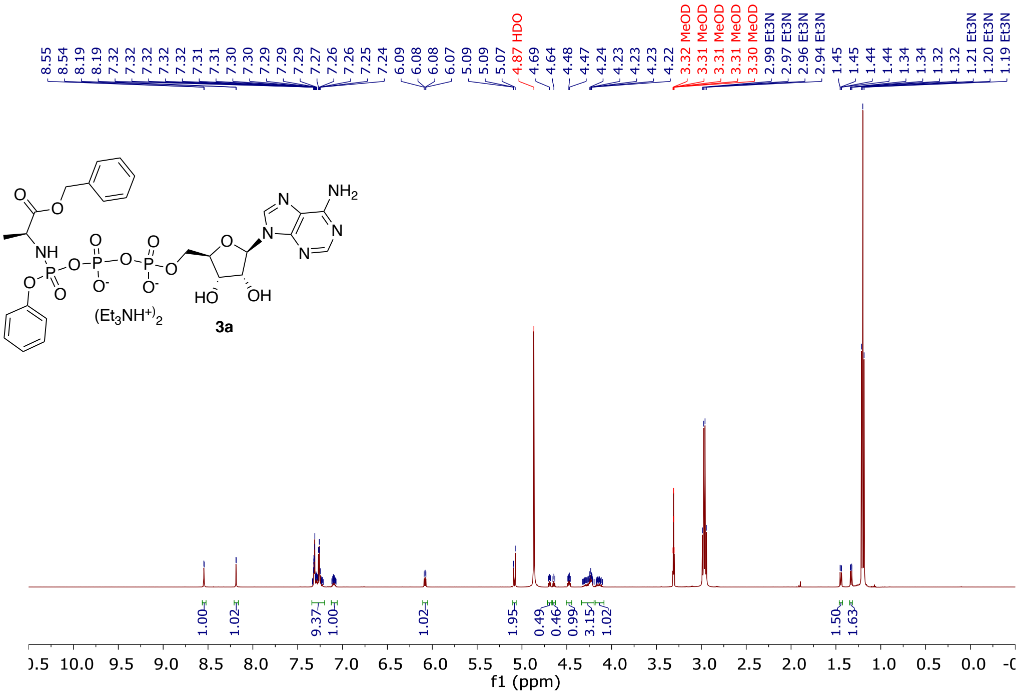

^{1}H-NMR (500 MHz, CD_3OD)

Prodrug **3a**

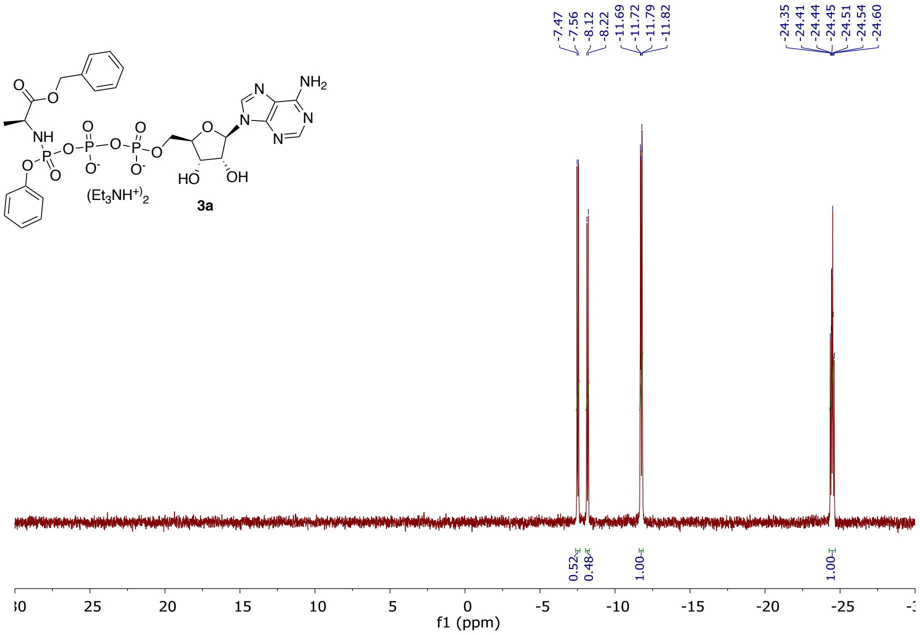

^{31}P-NMR (202 MHz, CD_3OD)

Prodrug **3a**

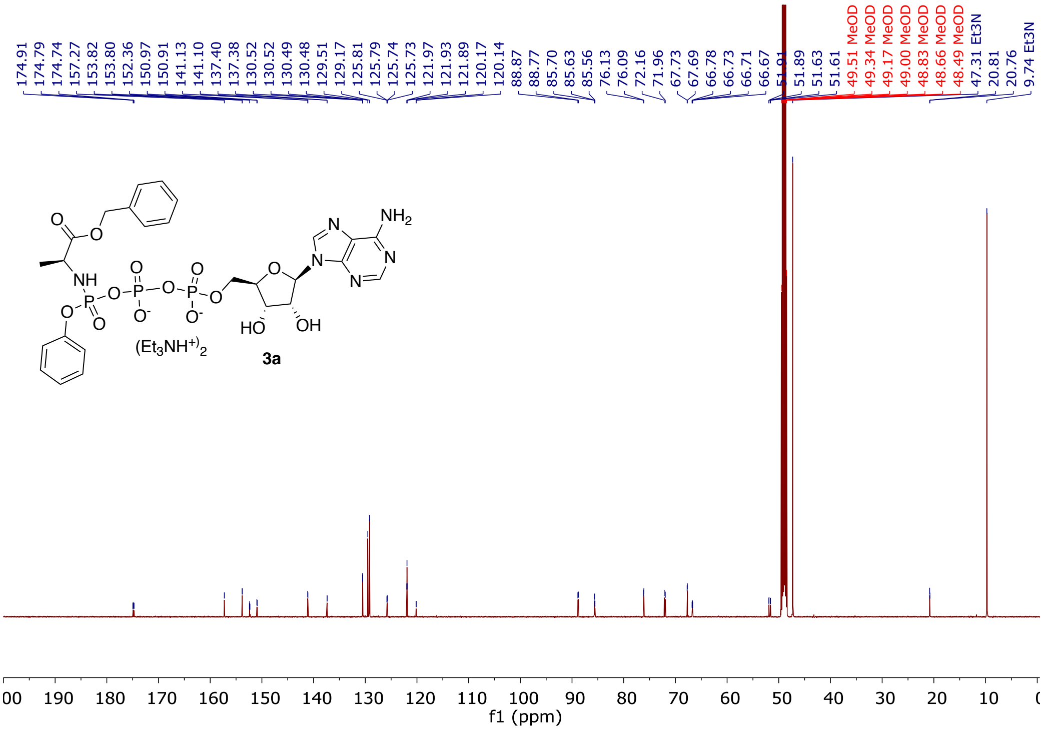

^{13}C-NMR (126 MHz, CD_3OD)

Prodrug **3a**

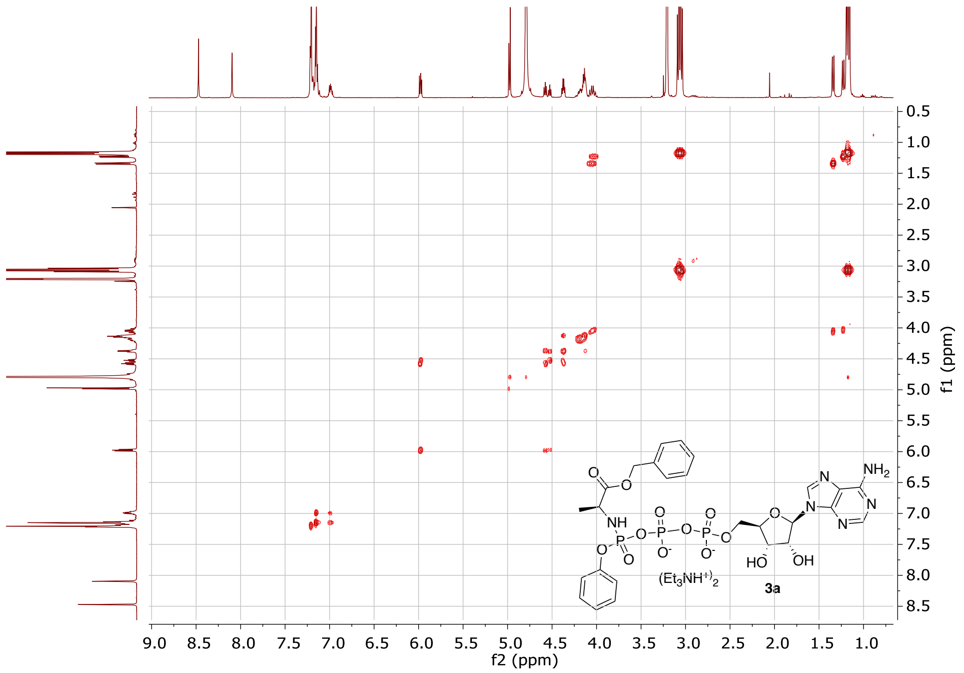

COSY (500 MHz, CD_3OD)

Prodrug **3a**

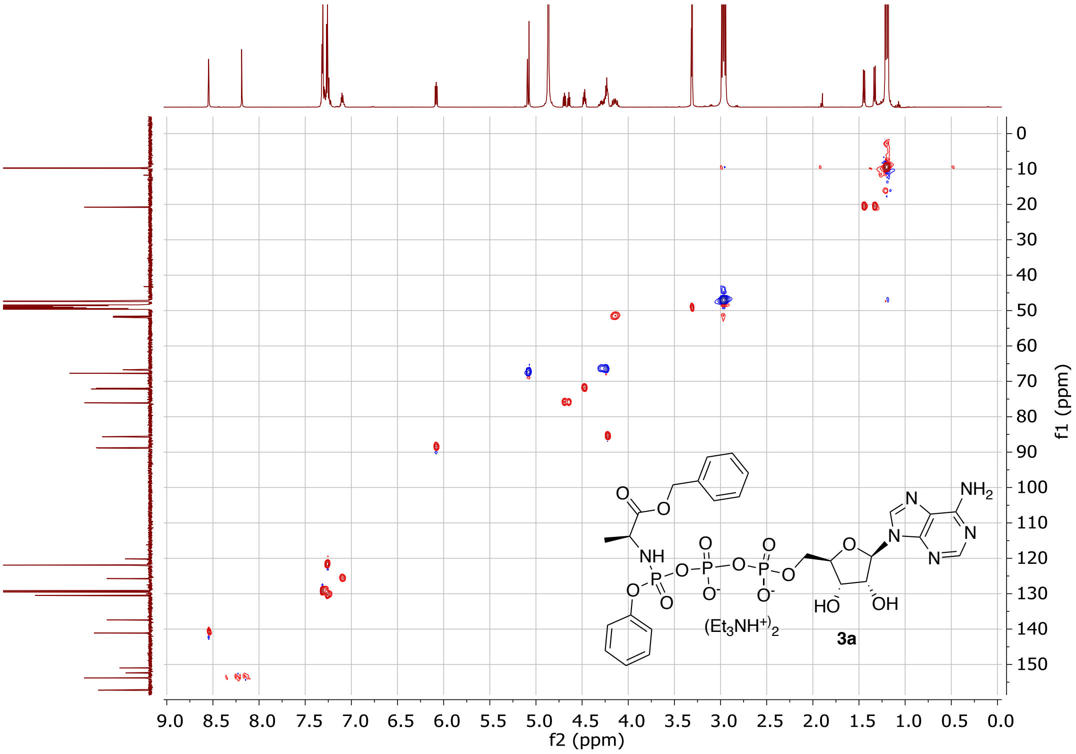

HSQC (500 MHz, CD$_3$OD)

Prodrug **3a**

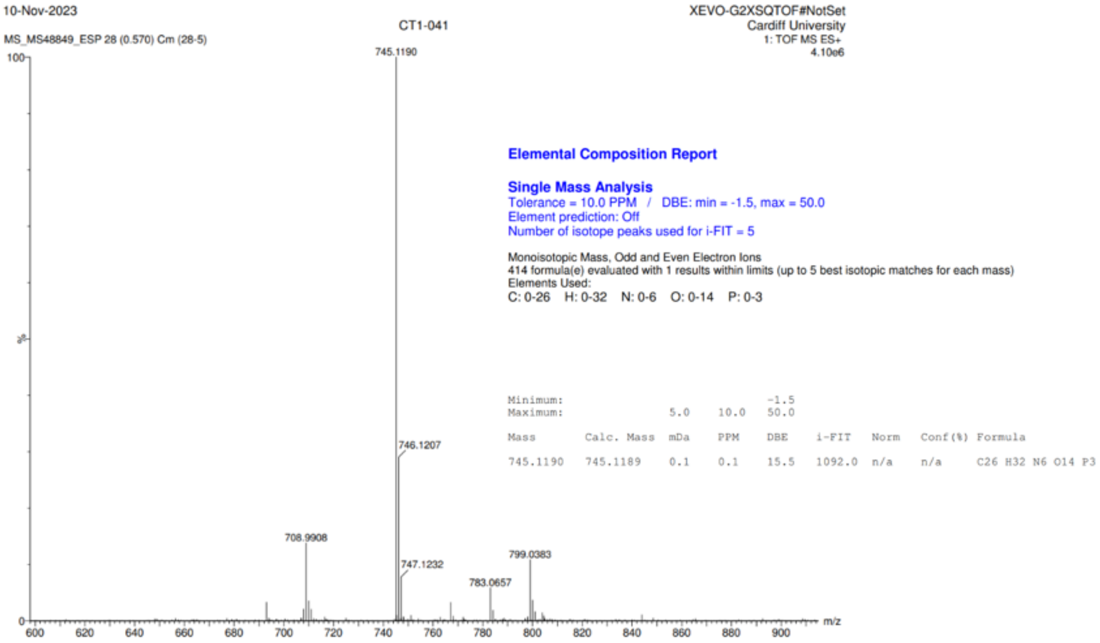

HRMS

Prodrug **3a**

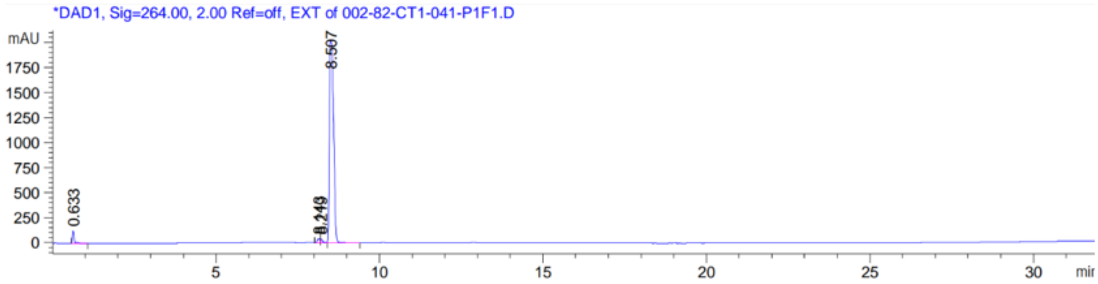

Peak #	RetTime [min]	Type	Width [min]	Area [mAU*s]	Height [mAU]	Area %
1	0.633	BV R	0.0526	448.64160	122.63389	2.3849
2	8.143	BV E	0.0813	208.28516	39.62725	1.1072
3	8.219	VV E	0.0741	184.33331	35.78508	0.9799
4	8.507	VV R	0.1451	1.79708e4	2025.98596	95.5281
Totals :				1.88121e4	2224.03218	

HPLC chromatogram

Prodrug **3a**

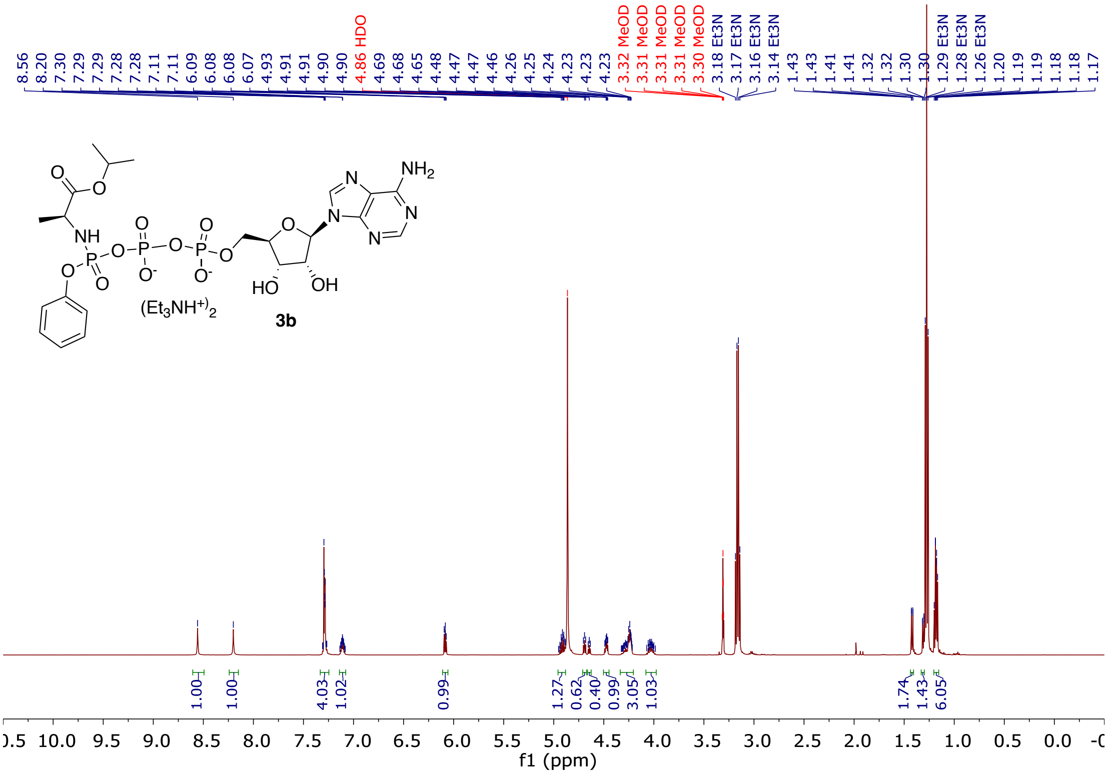

^{1}H-NMR (500 MHz, CD_3OD)

Prodrug **3b**

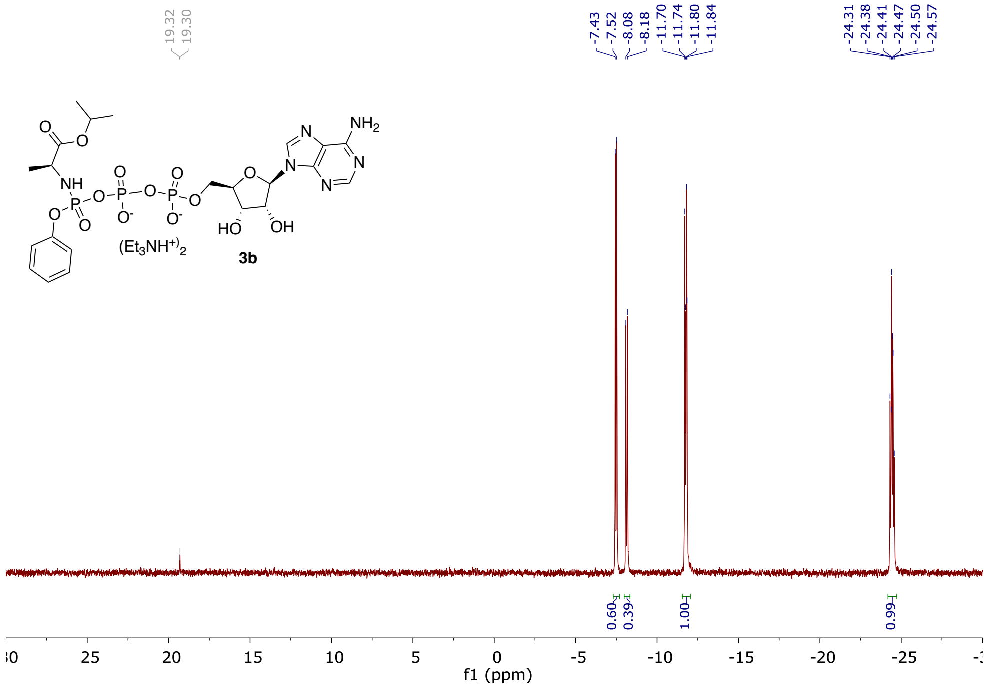

^{31}P-NMR (202 MHz, CD_3OD)

Prodrug **3b**

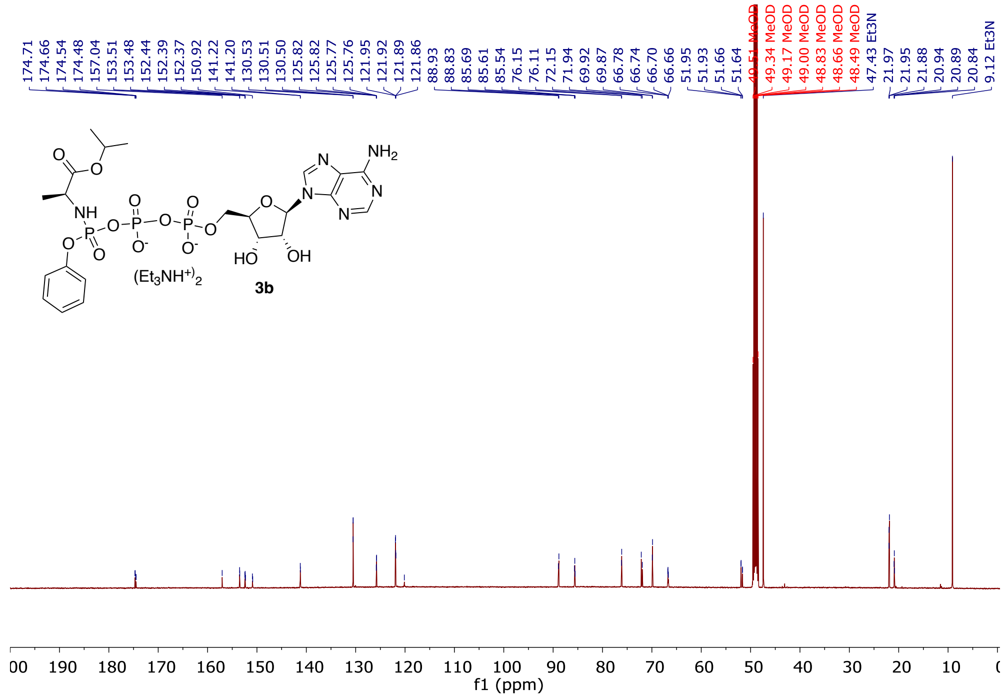

^{13}C-NMR (126 MHz, CD_3OD)

Prodrug **3b**

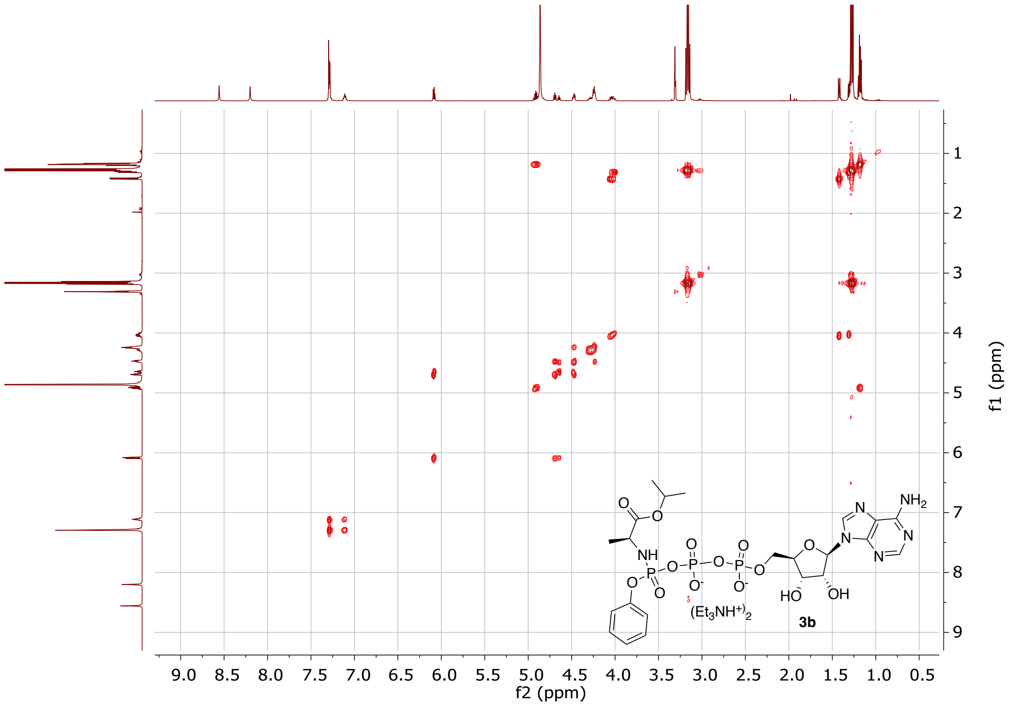

COSY (500MHz, CD_3OD)

Prodrug **3b**

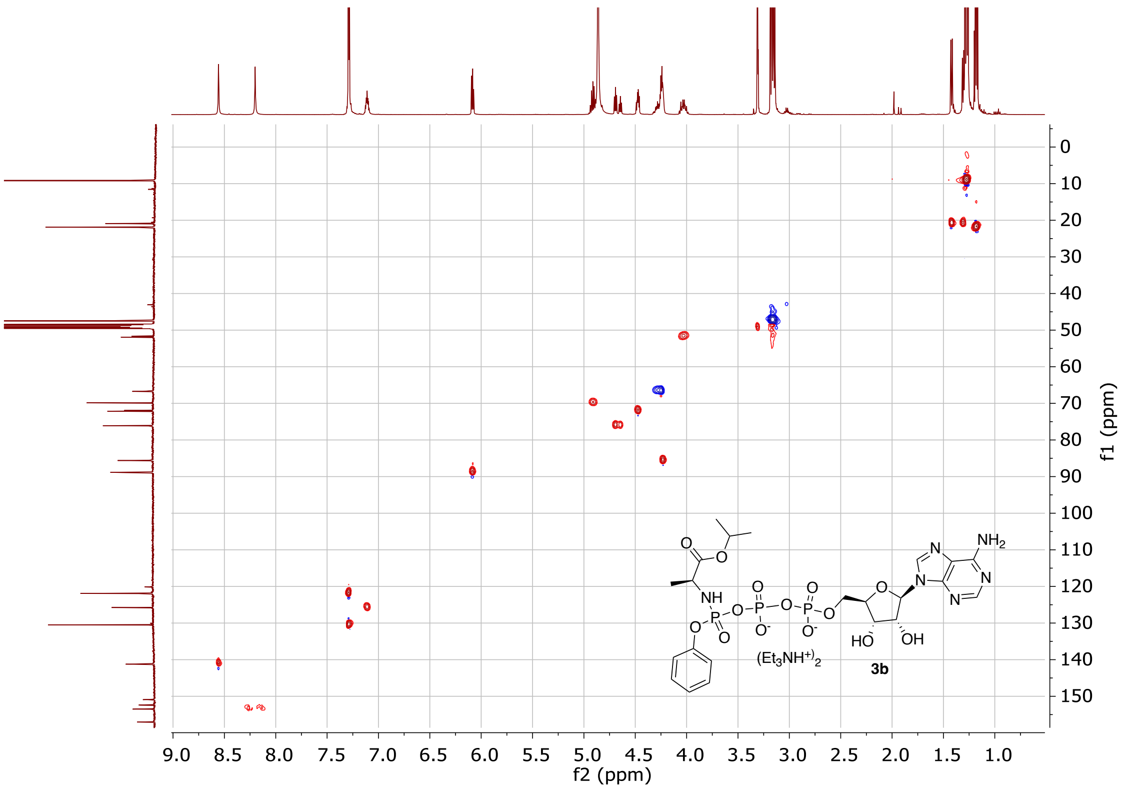

HSQC (500 MHz, CD_3OD)

Prodrug **3b**

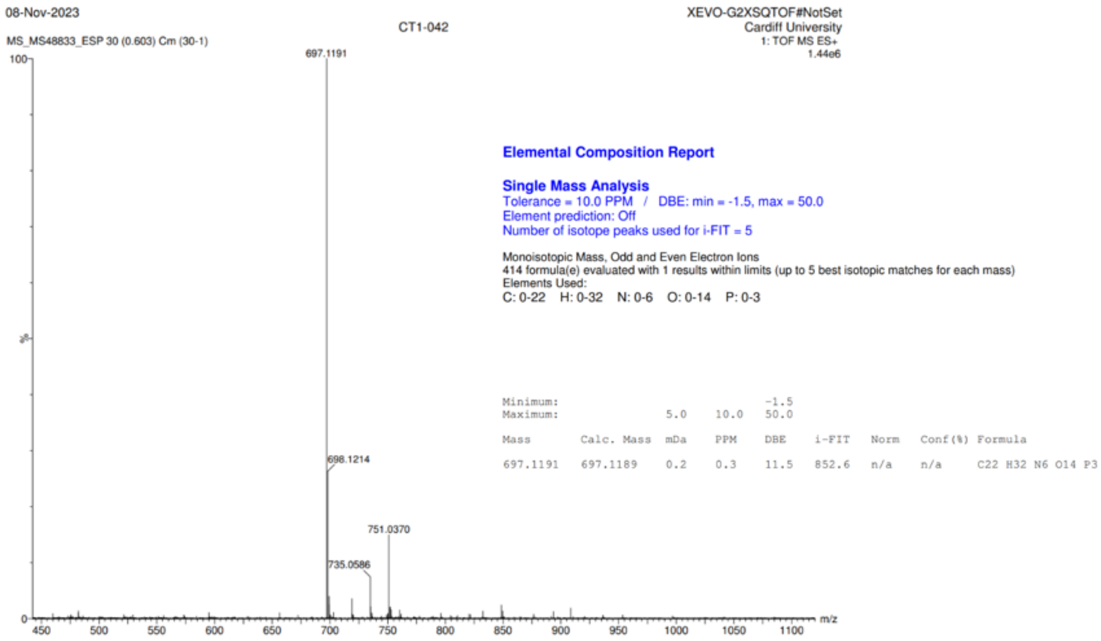

HRMS

Prodrug **3b**

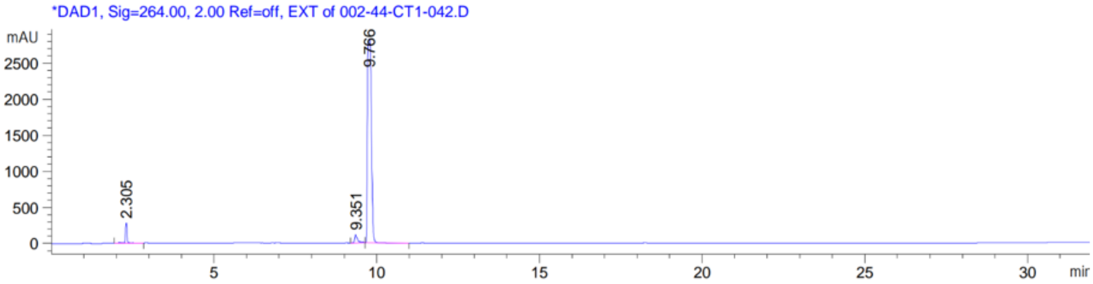

Peak #	RetTime [min]	Type	Width [min]	Area [mAU*s]	Height [mAU]	Area %
1	2.305	VV R	0.0493	1004.92804	279.85989	3.9499
2	9.351	BV R	0.0817	766.00610	115.35534	3.0108
3	9.766	BV R	0.1356	2.36708e4	2827.08496	93.0393
Totals :				2.54417e4	3222.30019	

HPLC chromatogram

Prodrug **3b**

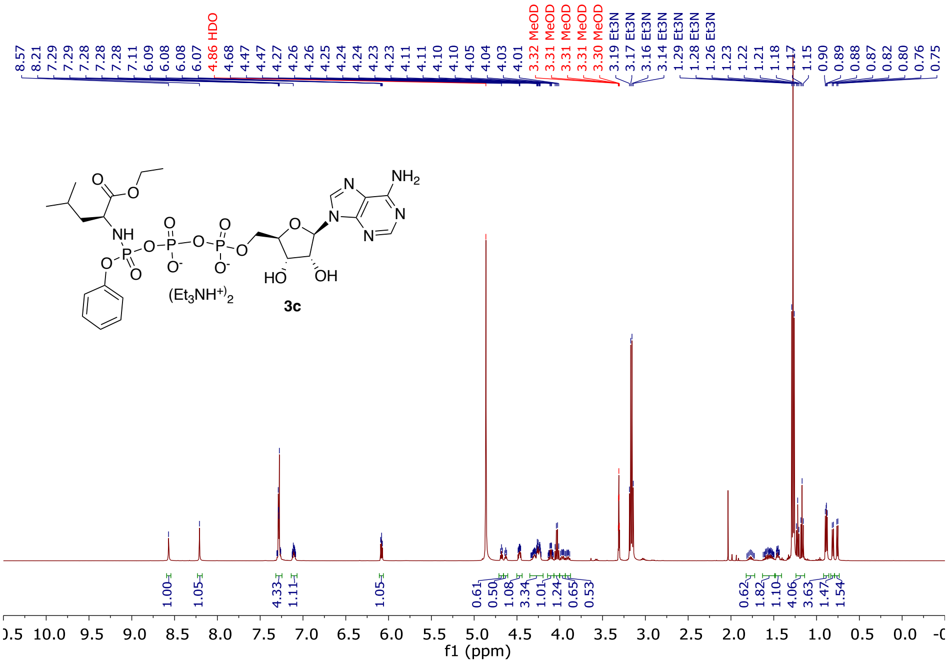

^{1}H-NMR (500 MHz, CD_3OD)

Prodrug **3c**

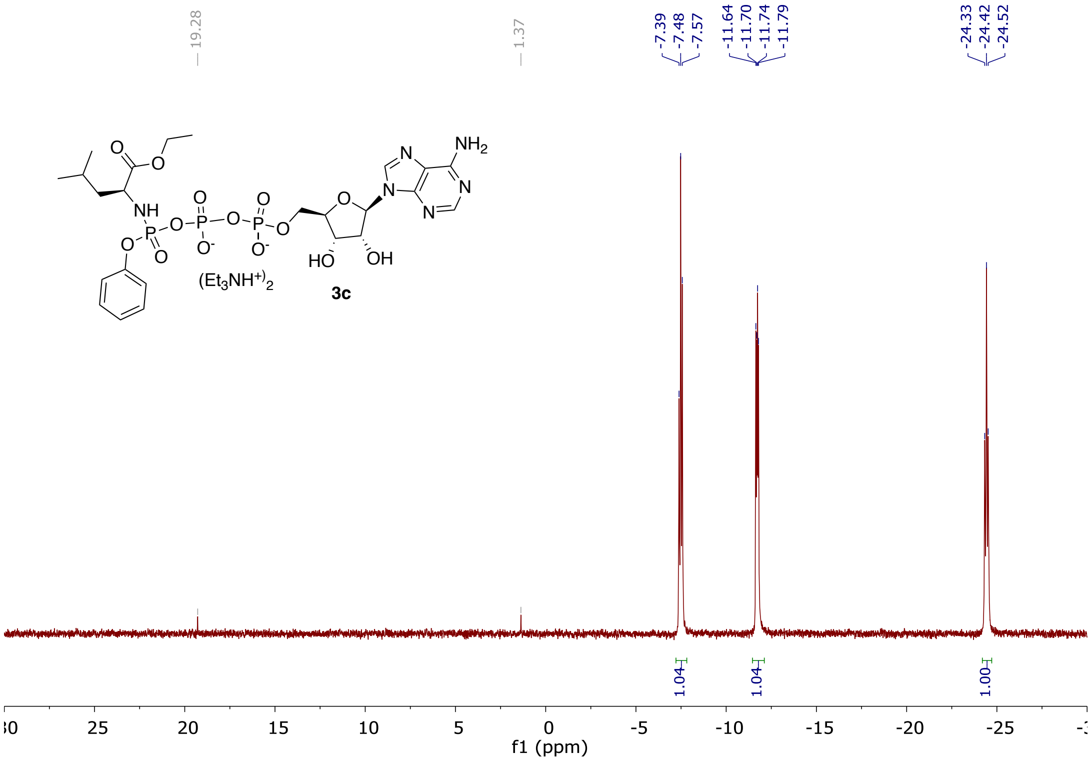

^{31}P-NMR (202 MHz, CD_3OD)

Prodrug **3c**

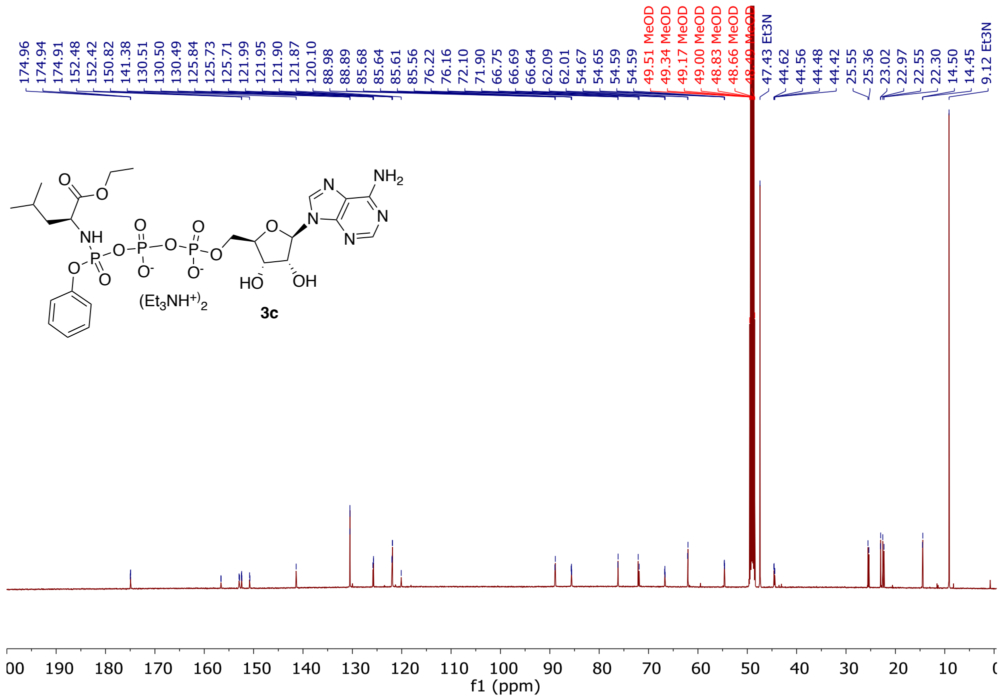

^{13}C-NMR (126 MHz, CD_3OD)

Prodrug **3c**

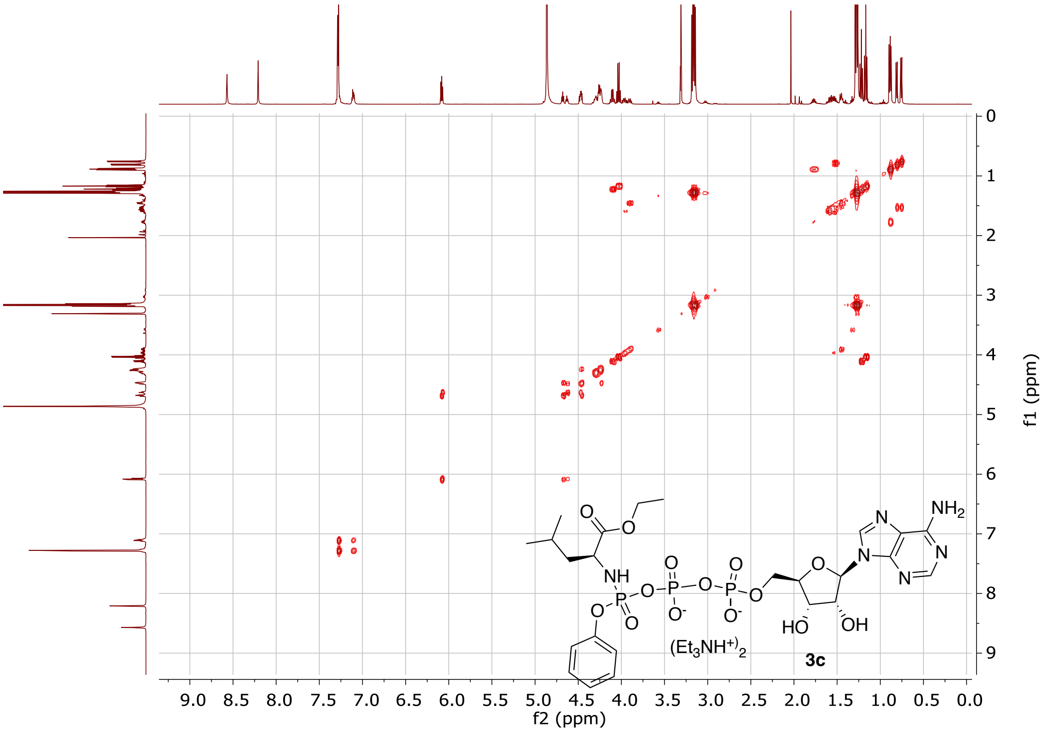

COSY NMR (500 MHz, CD_3OD)

Prodrug **3c**

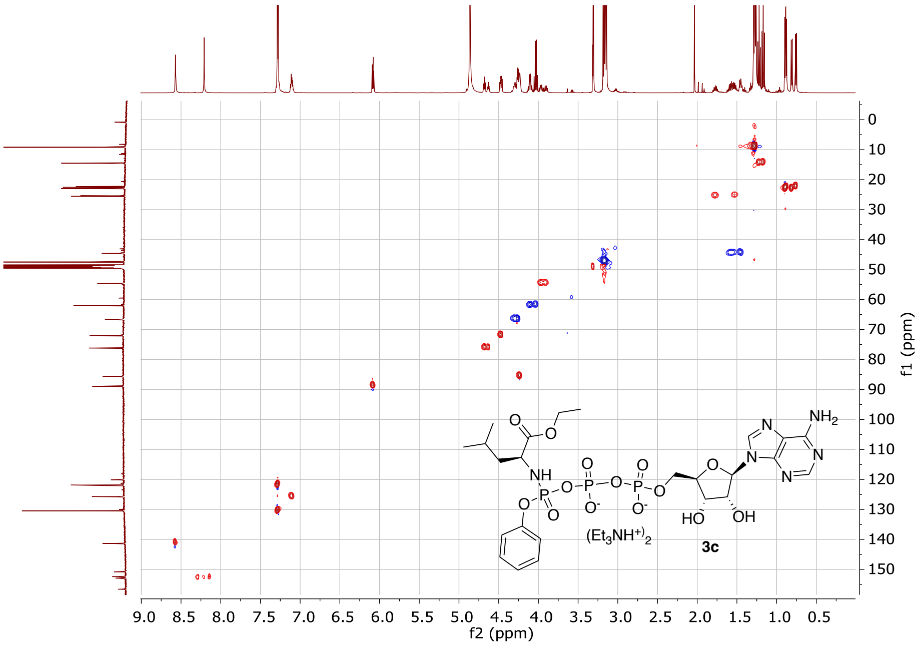

HSQC NMR (500 MHz, CD_3OD)

Prodrug **3c**

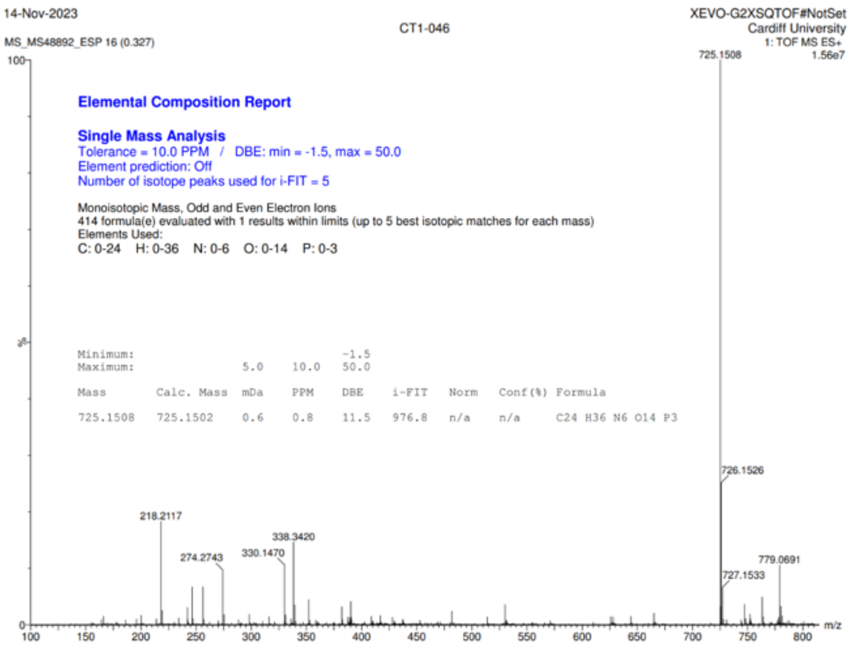

HRMS

Prodrug **3c**

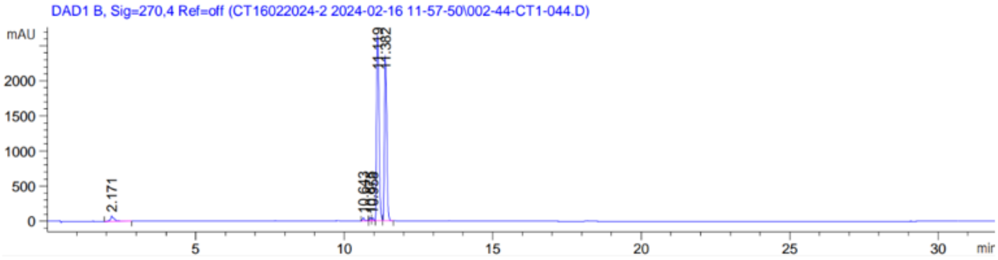

Peak #	RetTime [min]	Type	Width [min]	Area [mAU*s]	Height [mAU]	Area %
1	2.171	BB	0.1285	743.30493	75.71107	2.5091
2	10.643	BV R	0.0752	227.63297	45.15889	0.7684
3	10.875	BV E	0.0520	152.64040	44.48583	0.5152
4	10.958	VV E	0.0616	173.62875	40.80389	0.5861
5	11.119	VV R	0.0950	1.56058e4	2639.68286	52.6785
6	11.382	VB	0.0873	1.27216e4	2343.93506	42.9427
Totals :				2.96246e4	5189.77760	

HPLC chromatogram

Prodrug **3c**

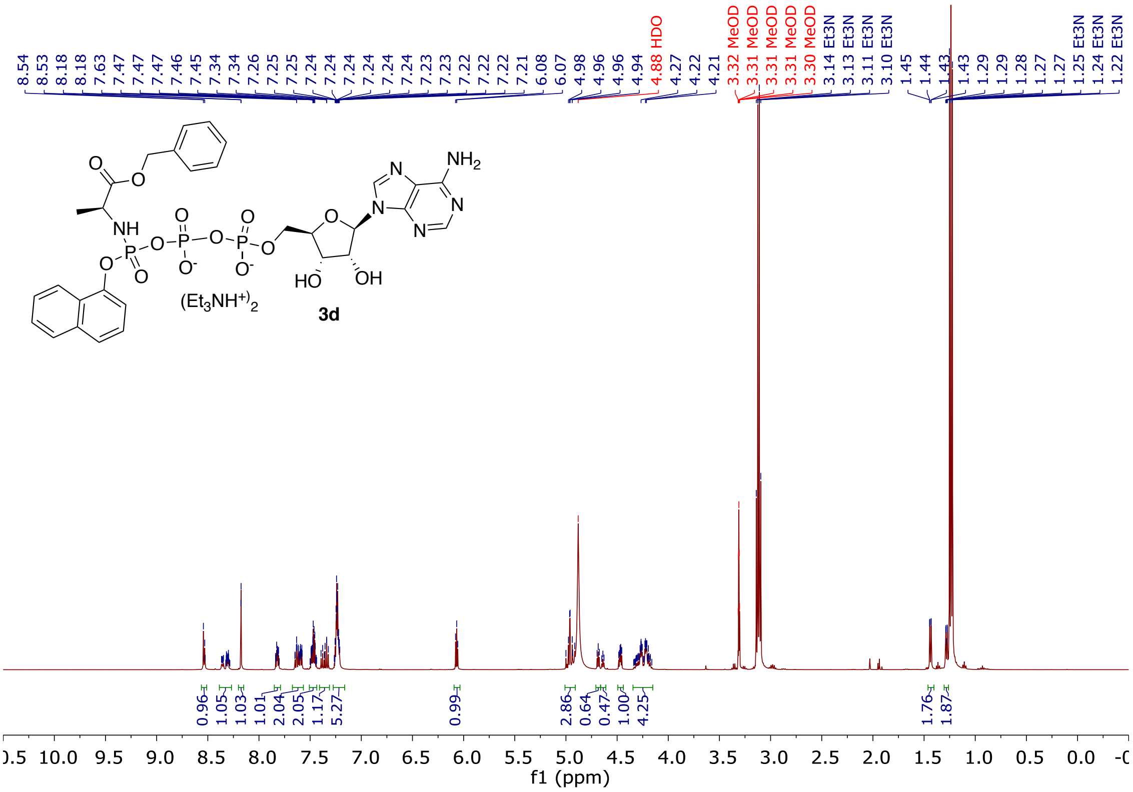

^{1}H-NMR (500 MHz, CD_3OD)

Prodrug **3d**

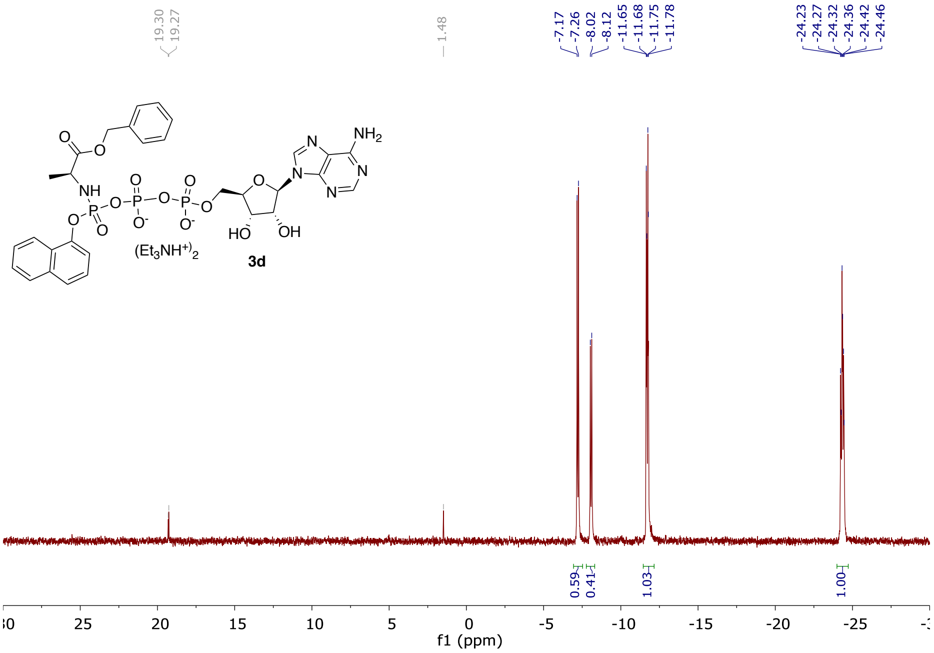

^{31}P-NMR (202 MHz, CD_3OD)

Prodrug **3d**

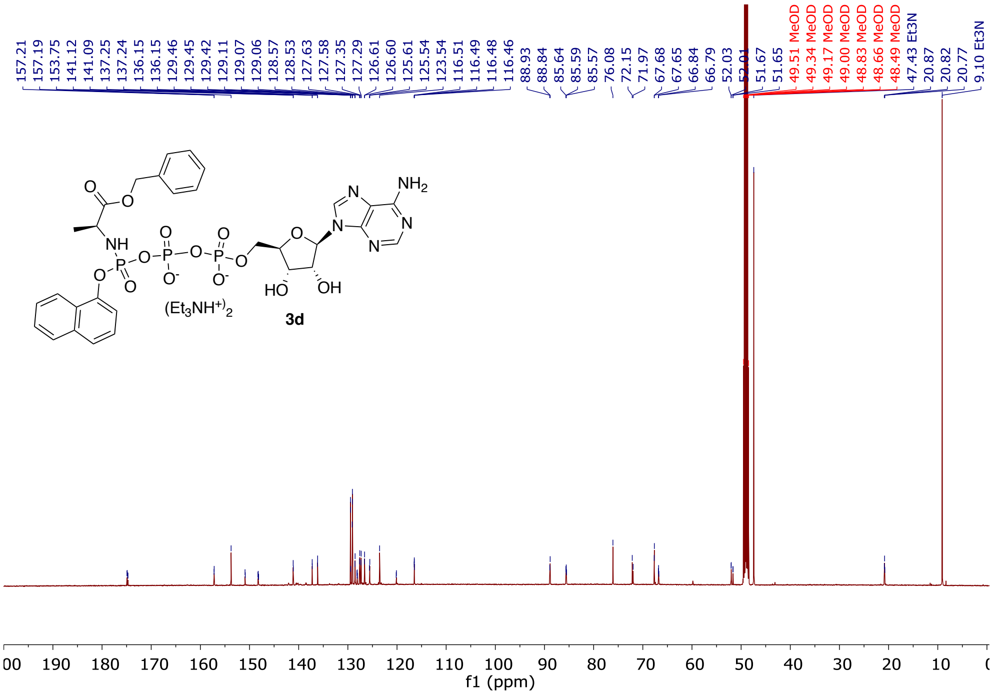

^{13}C-NMR (126 MHz, CD_3OD)

Prodrug **3d**

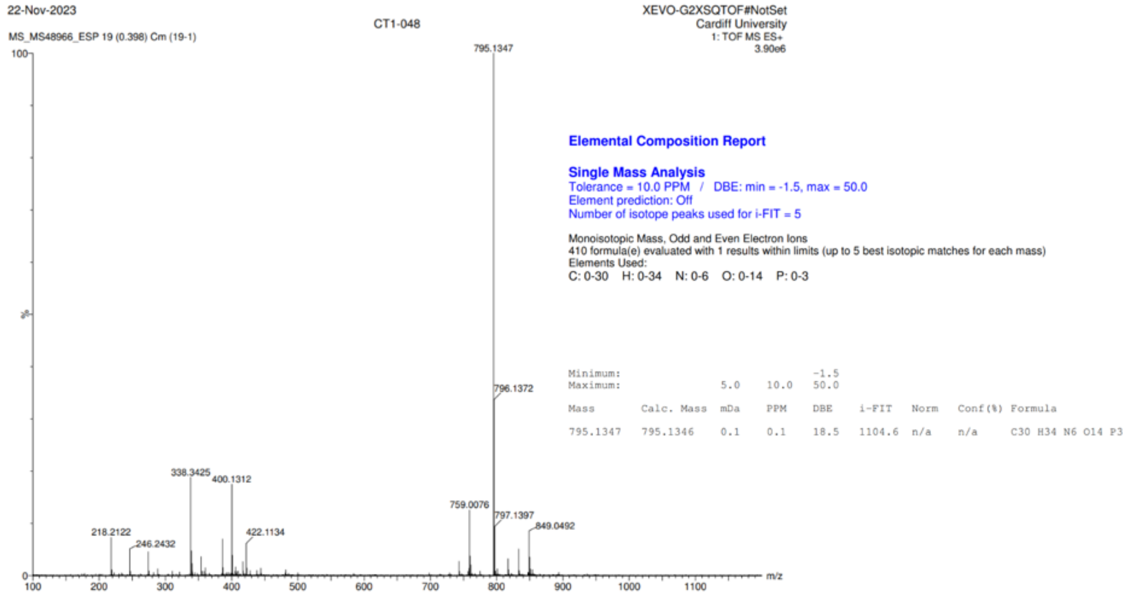

HRMS

Prodrug **3d**

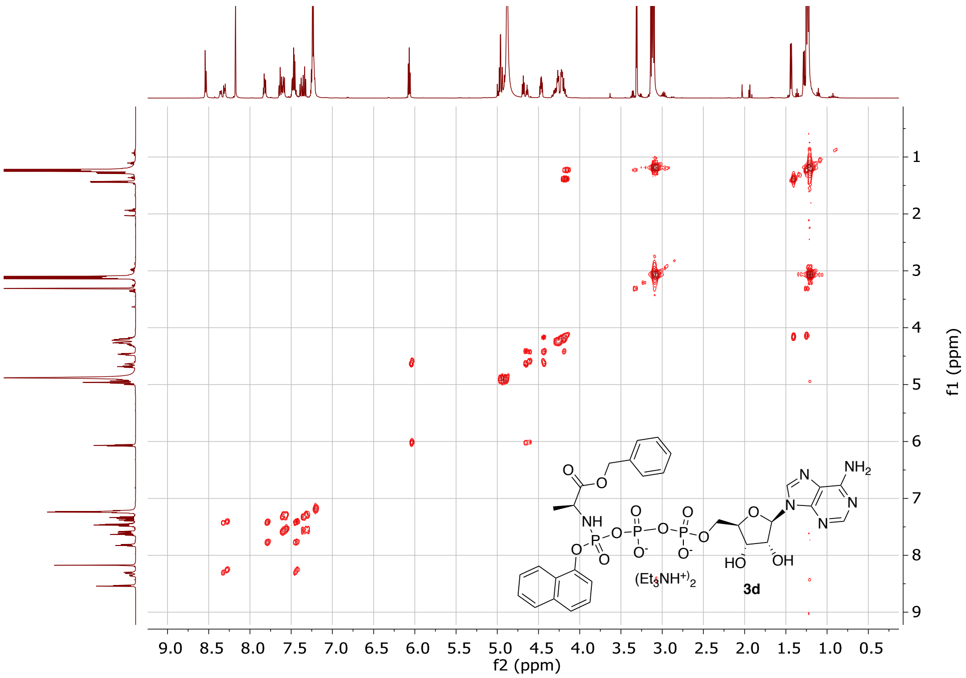

COsy NMR (500 MHz, CD_3OD)

Prodrug **3d**

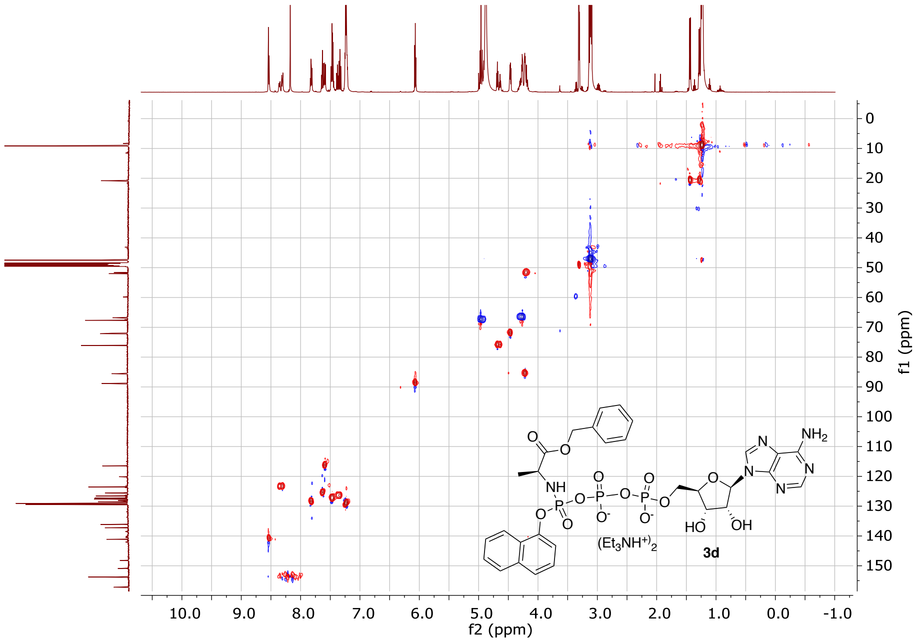

HSQC NMR (500 MHz, CD_3OD)

Prodrug **3d**

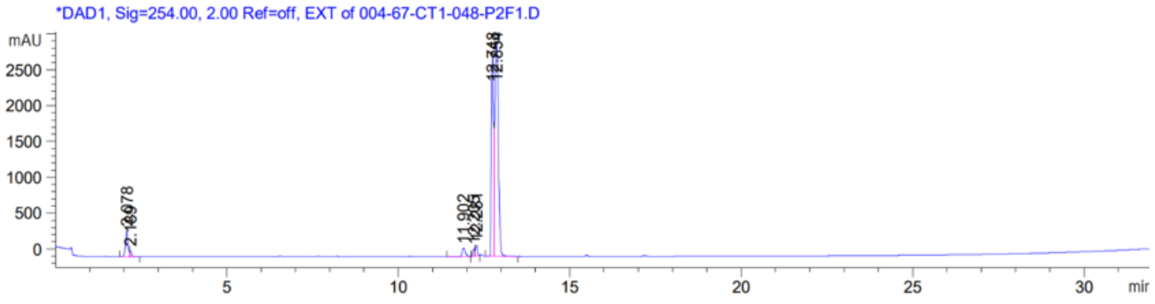

Peak #	RetTime [min]	Type	Width [min]	Area [mAU*s]	Height [mAU]	Area %
1	2.078	BV	0.0703	1738.82288	360.47397	4.5215
2	2.189	VB	0.0519	272.25641	79.52419	0.7079
3	11.902	VB R	0.1016	750.42828	115.82564	1.9513
4	12.205	BV	0.0520	342.53690	99.94566	0.8907
5	12.281	VB	0.0628	615.63574	147.24599	1.6008
6	12.748	BV	0.0754	1.32497e4	2792.08154	34.4534
7	12.854	VV R	0.1138	2.14876e4	2988.59521	55.8743
Totals :				3.84570e4	6583.69220	

HPLC chromatogram

Prodrug **3d**

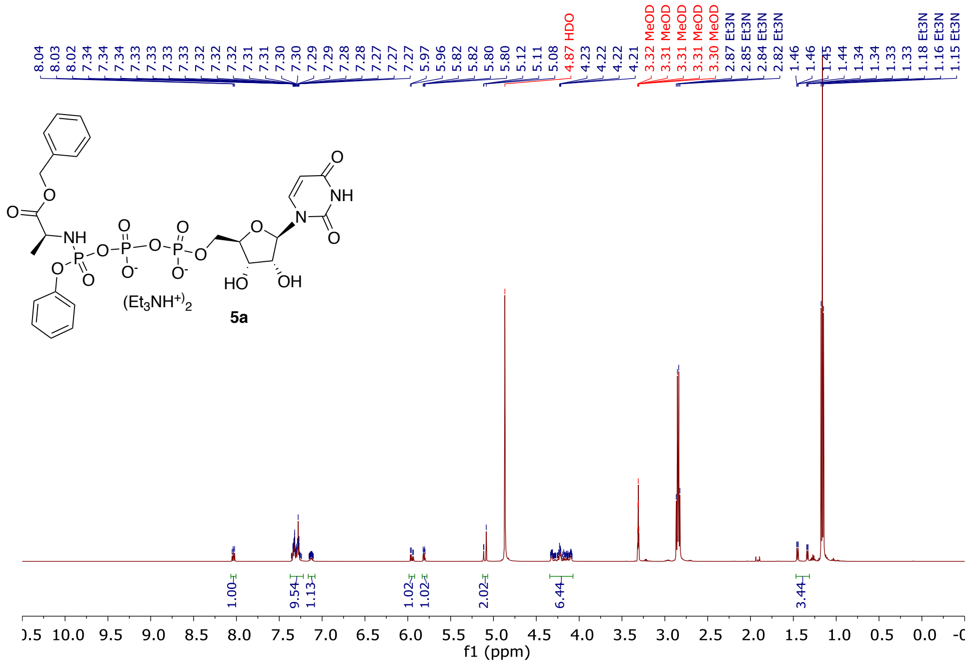

^{1}H-NMR (500 MHz, CD_3OD)

Prodrug **5a**

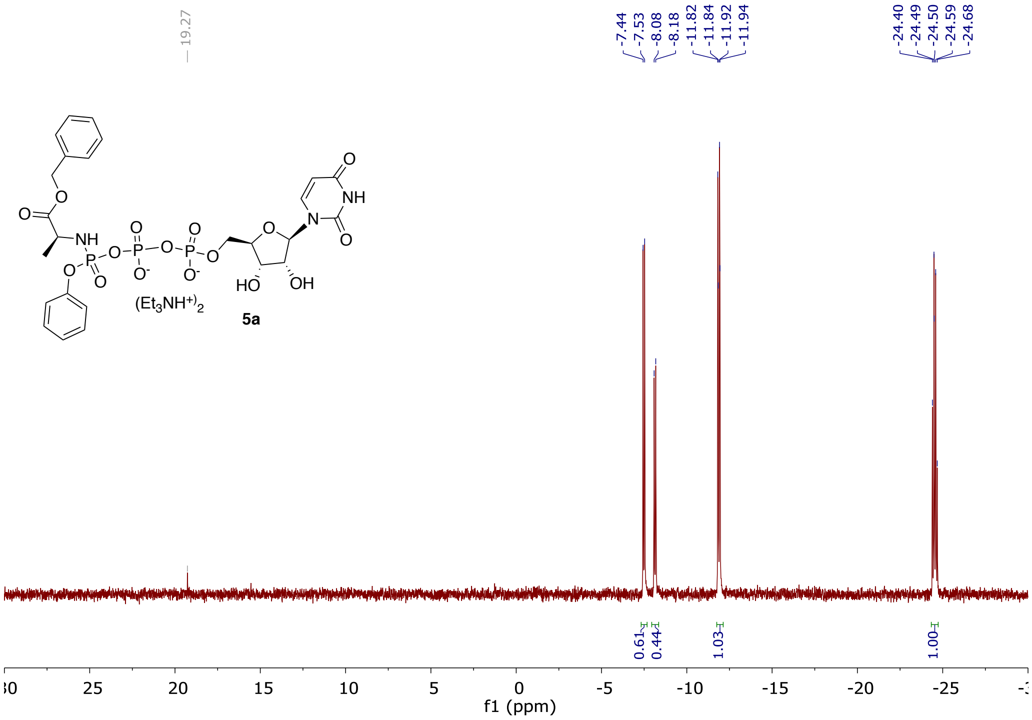

^{31}P-NMR (202 MHz, CD_3OD)

Prodrug **5a**

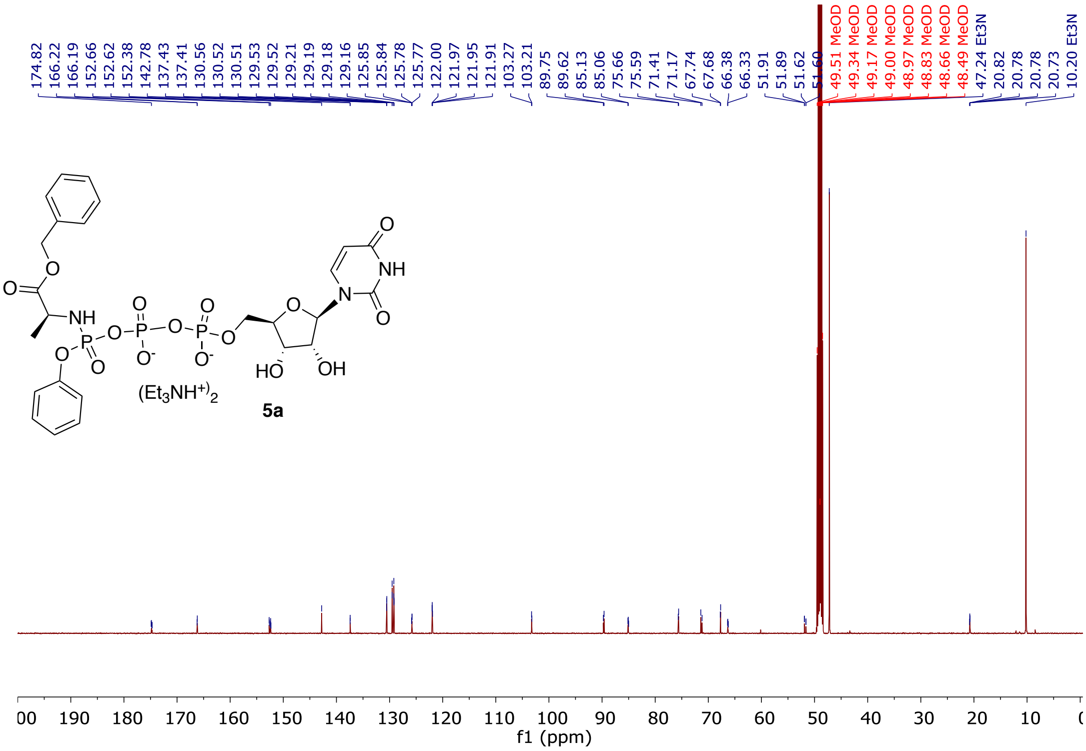

^{13}C-NMR (126 MHz, CD_3OD)

Prodrug **5a**

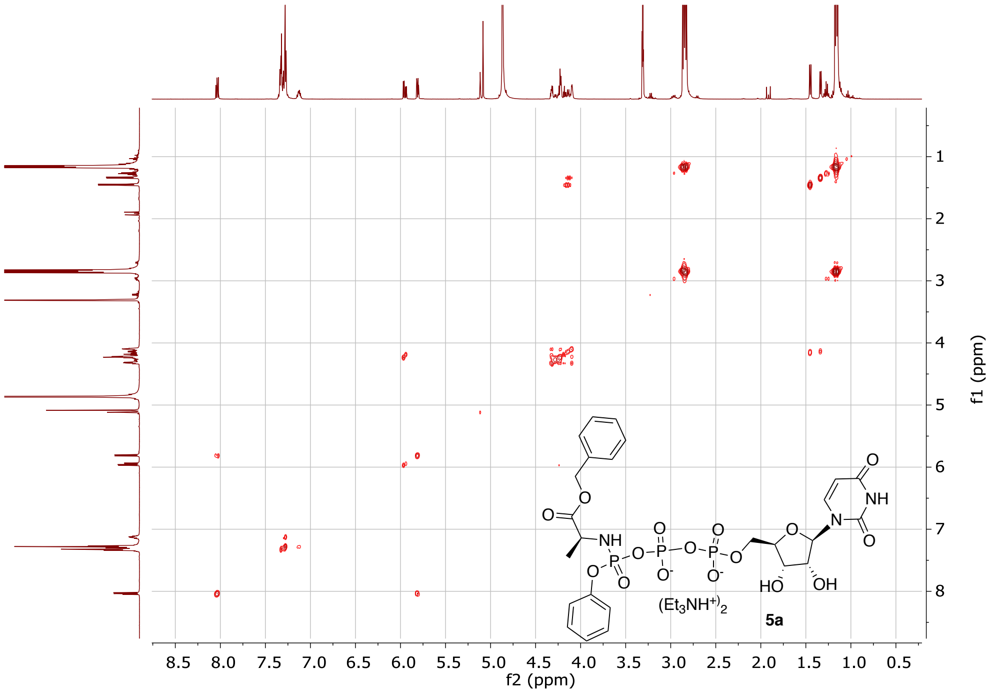

COSY (500MHz, CD_3OD)

Prodrug **5a**

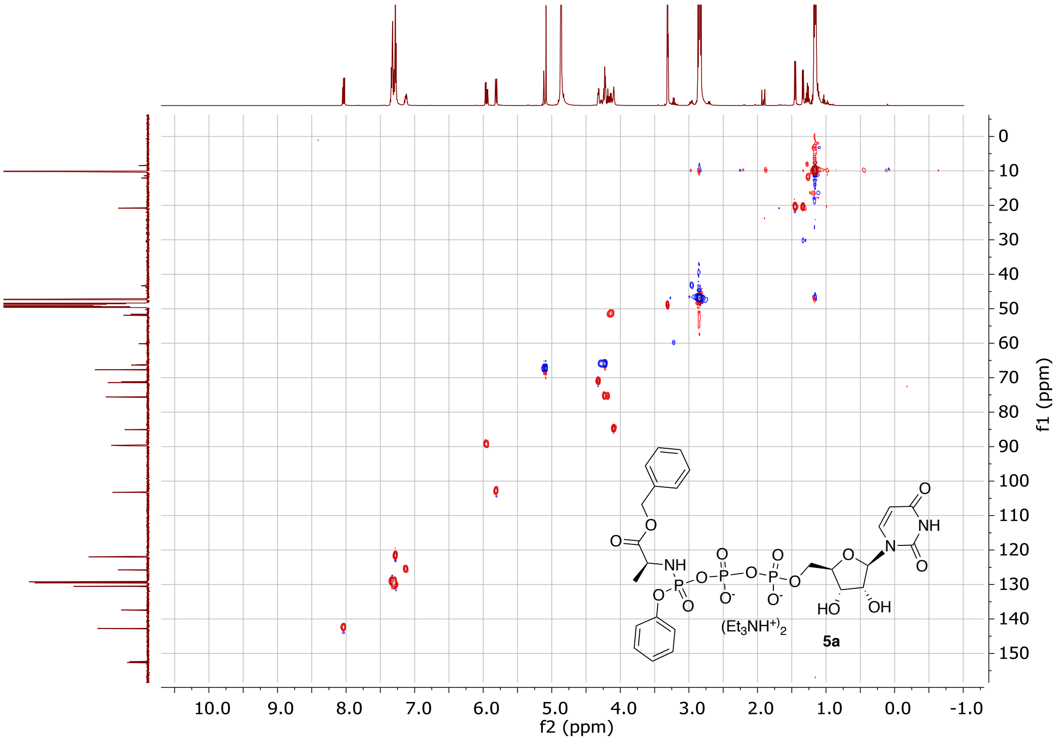

HSQC (500MHz, CD_3OD)

Prodrug **5a**

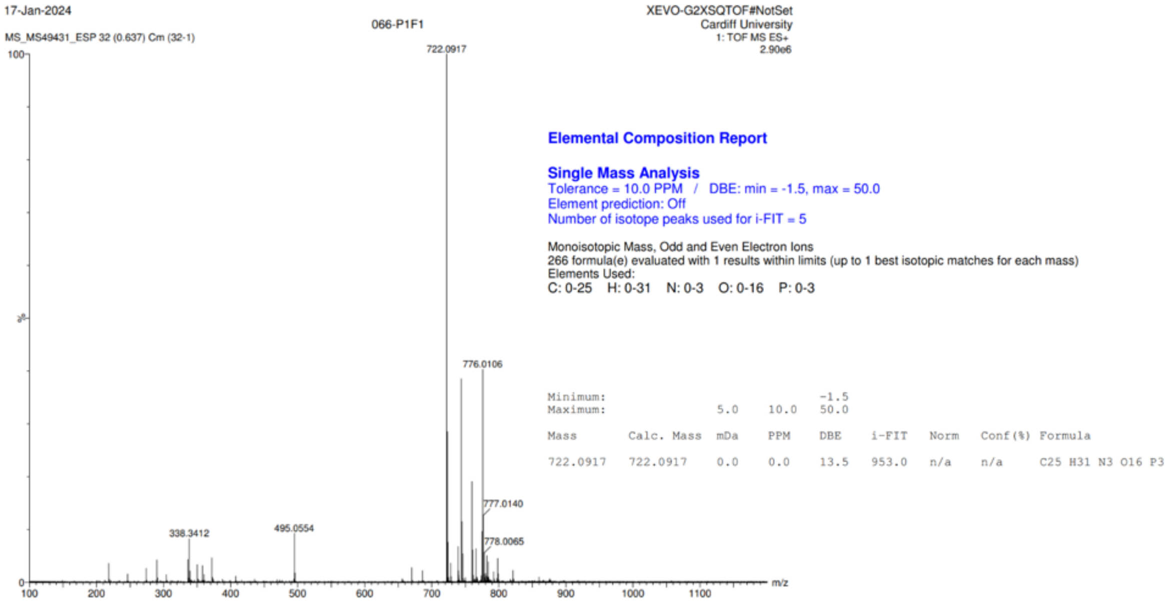

HRMS

Prodrug **5a**

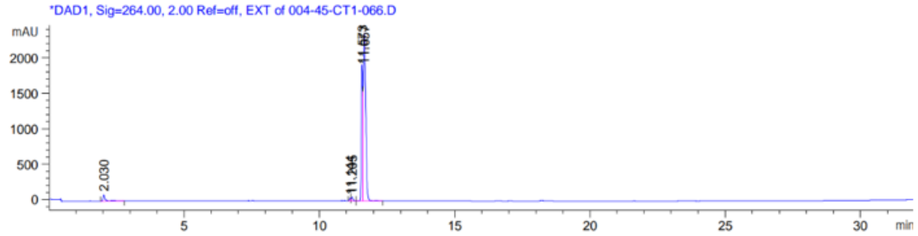

Peak #	RetTime [min]	Type	Width [min]	Area [mAU*s]	Height [mAU]	Area %
1	2.030	BV R	0.0795	626.40967	86.18652	2.6843
2	11.144	BV	0.0505	122.15932	37.04227	0.5235
3	11.205	VB	0.0659	214.14363	48.18206	0.9176
4	11.573	BV	0.0585	7313.42334	1919.48071	31.3392
5	11.657	VV R	0.0982	1.50602e4	2360.91602	64.5355
Totals :				2.33364e4	4451.80758	

HPLC chromatogram

Prodrug **5a**

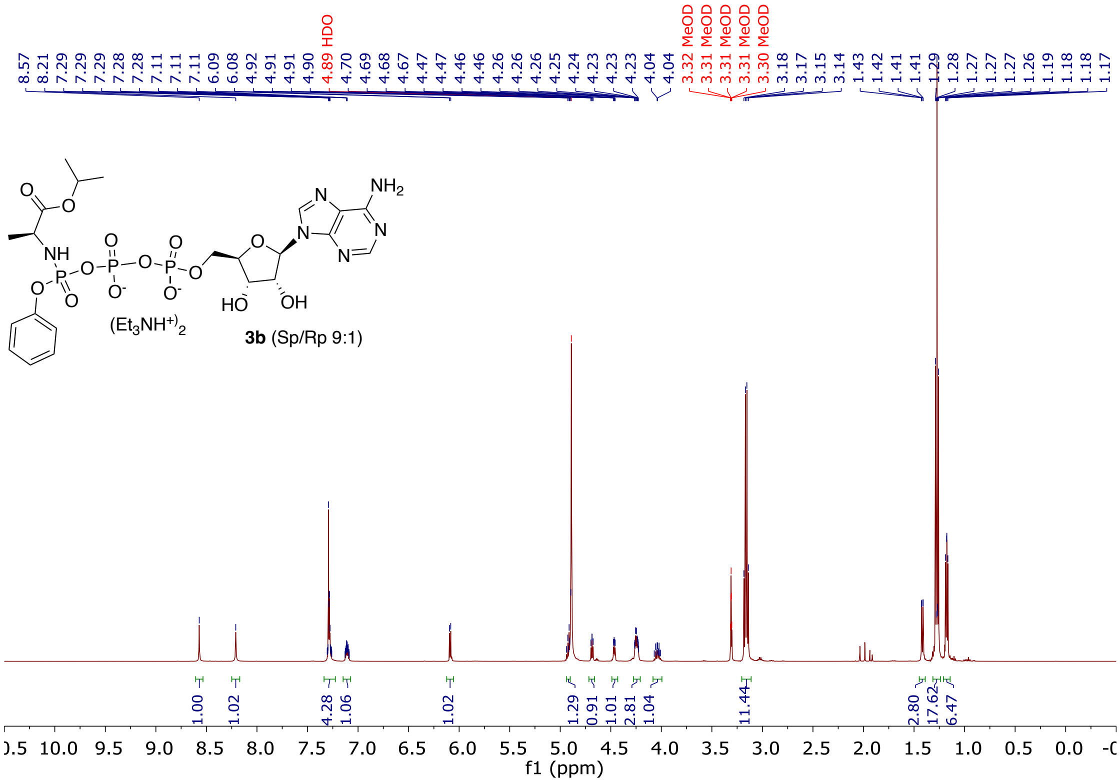

^{1}H-NMR (500 MHz, CD_3OD)

Prodrug **3b** (S_p/R_p 9:1)

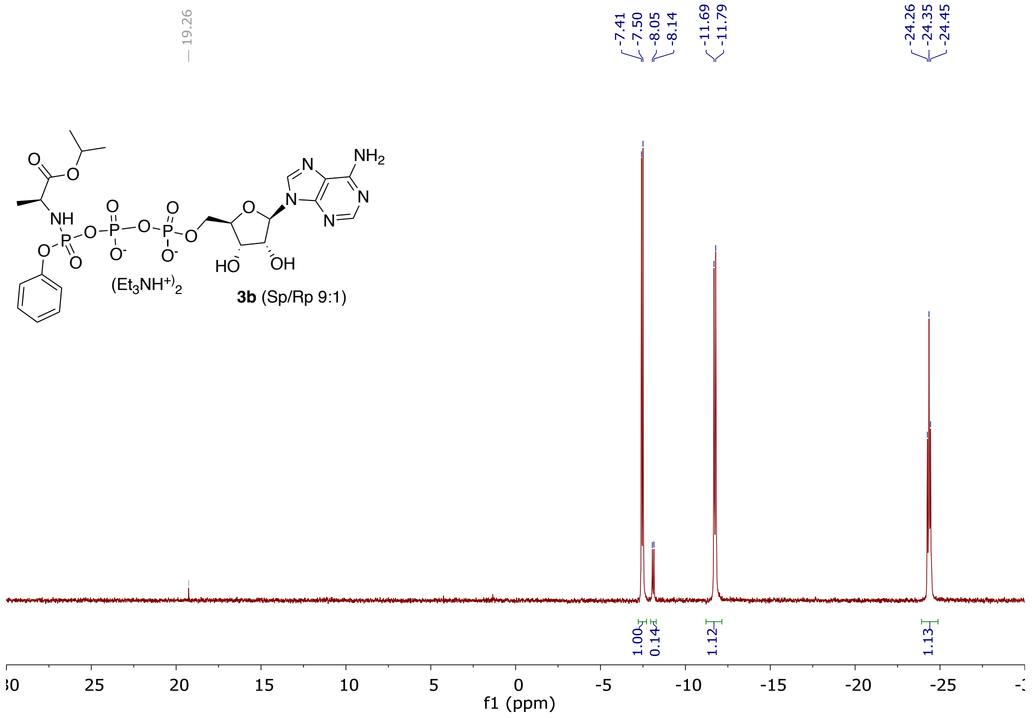

^{31}P-NMR (202 MHz, CD_3OD)

Prodrug **3b** (S_p/R_p 9:1)

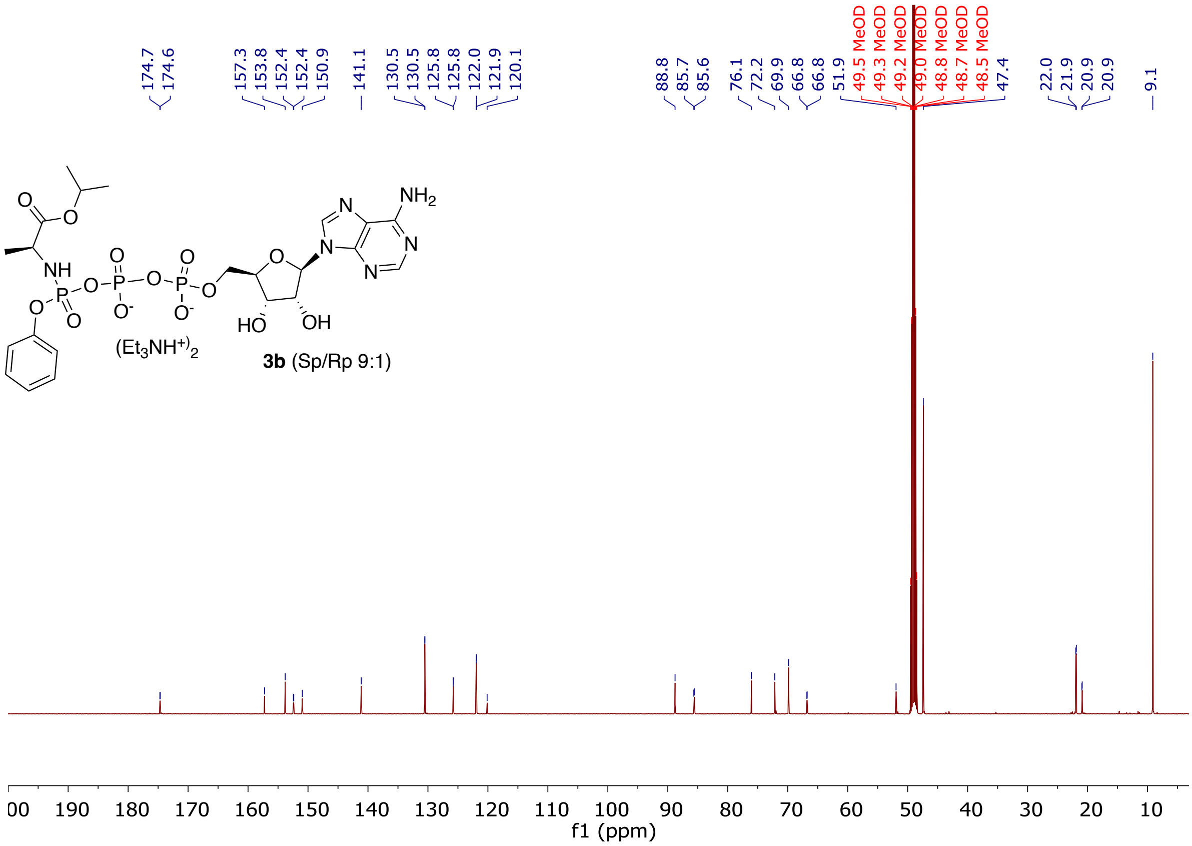

^{13}C-NMR (126 MHz, CD_3OD)

Prodrug **3b** (S_p/R_p 9:1)

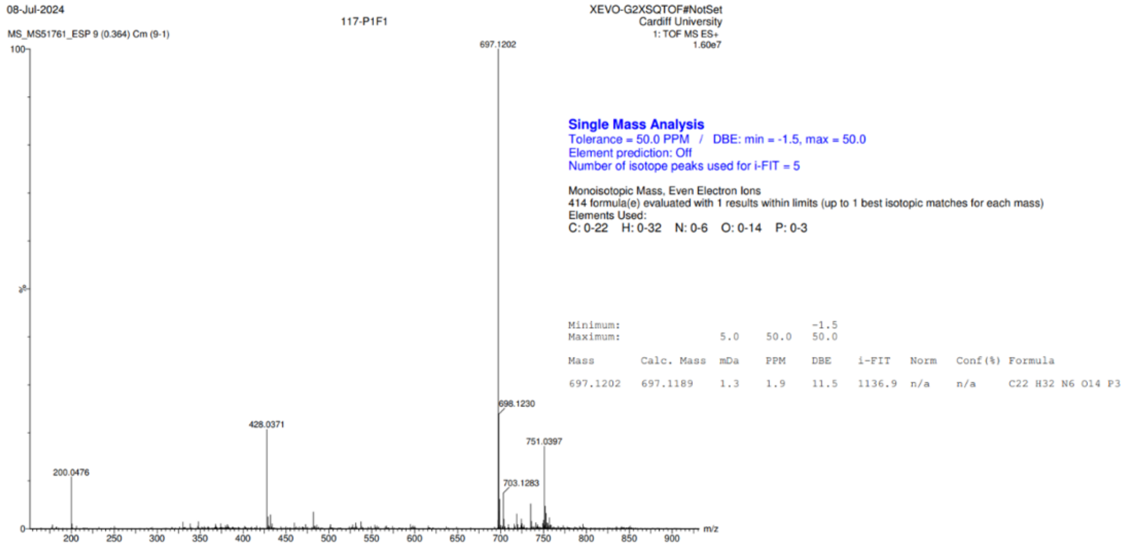

HRMS

Prodrug **3b** (S_p/R_p 9:1)

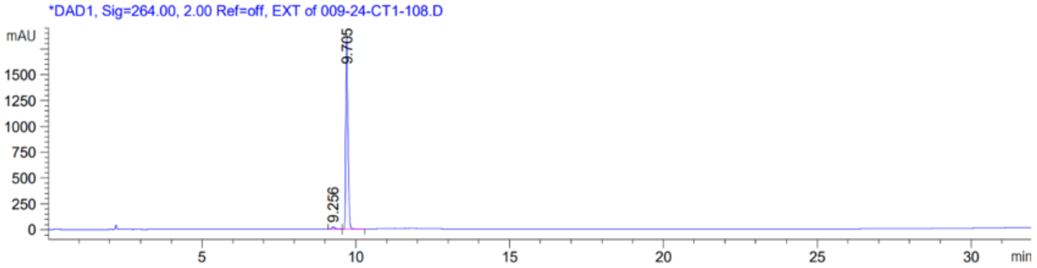

```
Signal 3: DAD1, Sig=264.00, 2.00 Ref=off, EXT of 009-24-CT1-108.D
 Signal has been modified after loading from rawdata file!
```

Peak #	RetTime [min]	Type	Width [min]	Area [mAU*s]	Height [mAU]	Area %
1	9.256	VV R	0.0963	164.10301	21.18310	1.7631
2	9.705	BV R	0.0774	9143.67285	1858.03723	98.2369
Totals :				9307.77586	1879.22033	

HPLC chromatogram

Prodrug **3b** (S_p/R_p 9:1)

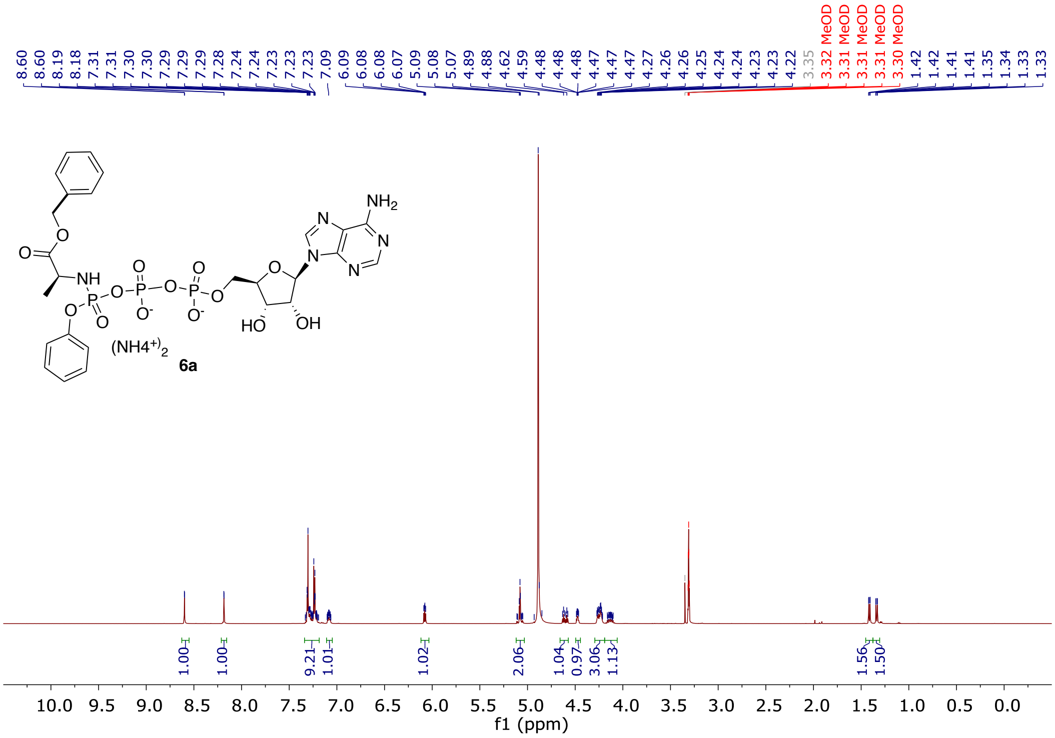

^{1}H-NMR (500 MHz, CD_3OD)

Prodrug **6a**

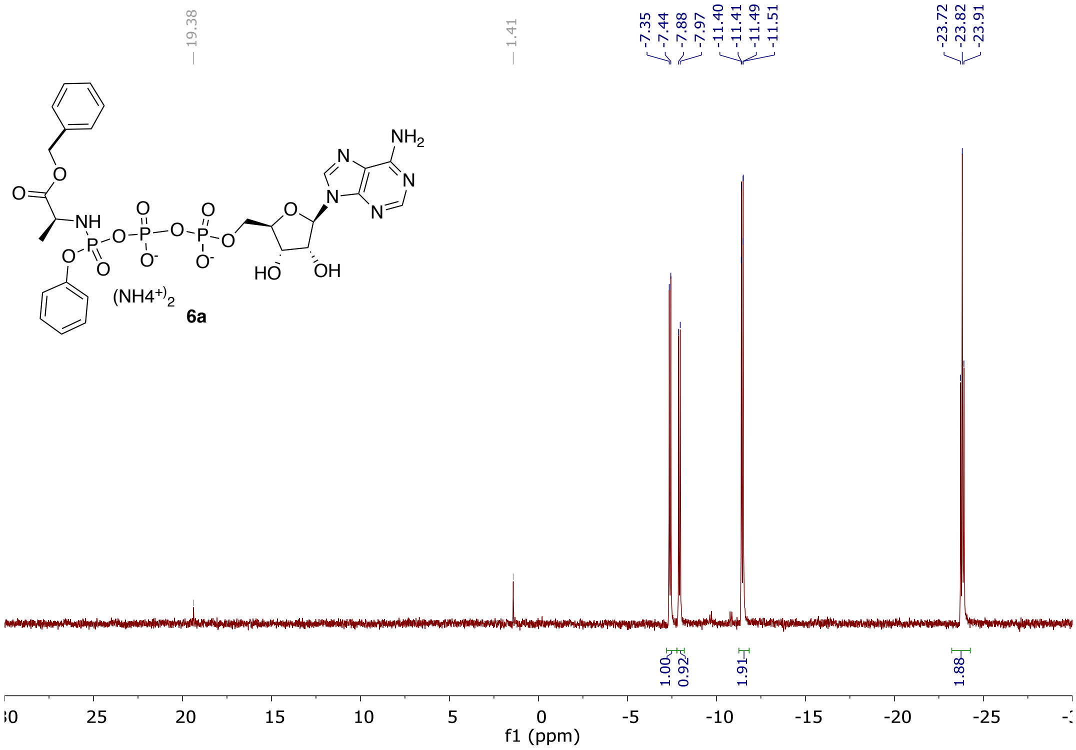

^{31}P-NMR (202 MHz, CD_3OD)

Prodrug **6a**

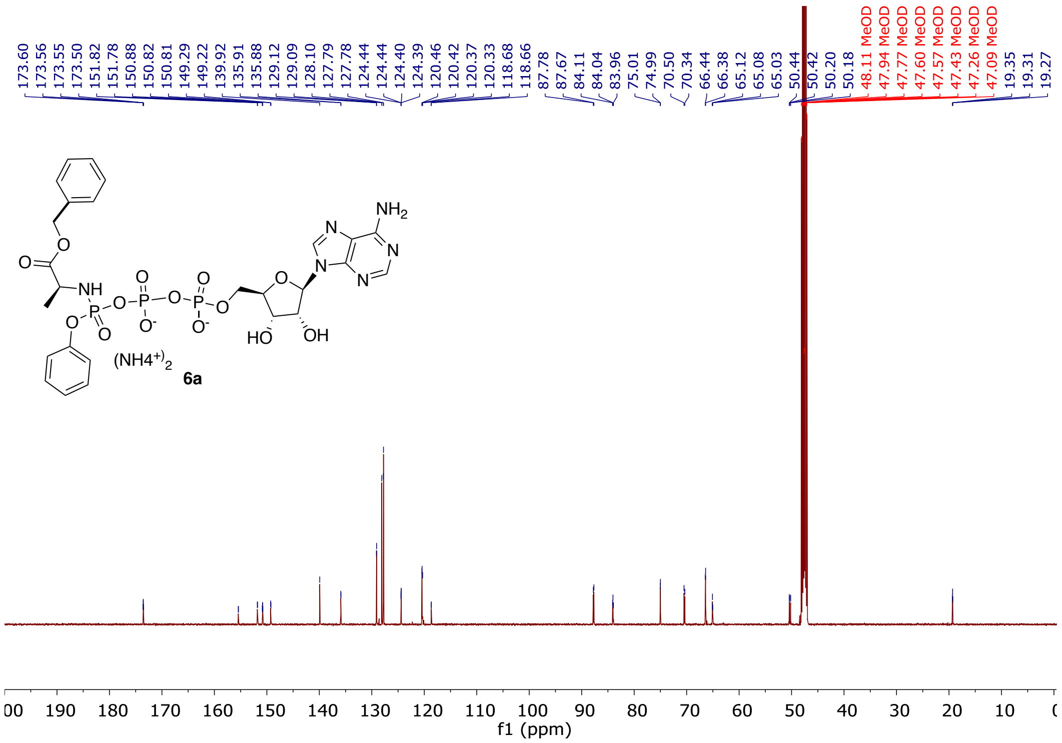

^{13}C-NMR (126 MHz, CD_3OD)

Prodrug **6a**

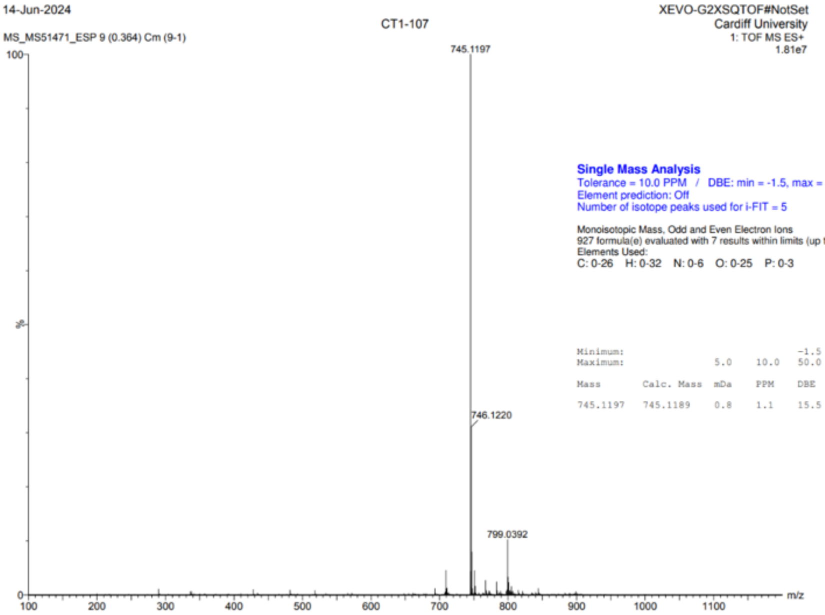

Single Mass Analysis
Tolerance = 10.0 PPM / DBE: min = -1.5, max = 50.0
Element prediction: Off
Number of isotope peaks used for i-FIT = 5

Monoisotopic Mass, Odd and Even Electron Ions
927 formula(e) evaluated with 7 results within limits (up to 1 best isotopic matches for each mass)
Elements Used:
C: 0-26 H: 0-32 N: 0-6 O: 0-25 P: 0-3

Minimum:				-1.5				
Maximum:		5.0	10.0	50.0				
Mass	Calc. Mass	mDa	PPM	DBE	i-FIT	Norm	Conf(%)	Formula
745.1197	745.1189	0.8	1.1	15.5	1110.8	n/a	n/a	C26 H32 N6 O14 P3

HRMS

Prodrug **6a**

^{1}H-, ^{19}F- and ^{13}C-NMR spectra and MS spectra of intermediates 9-10.

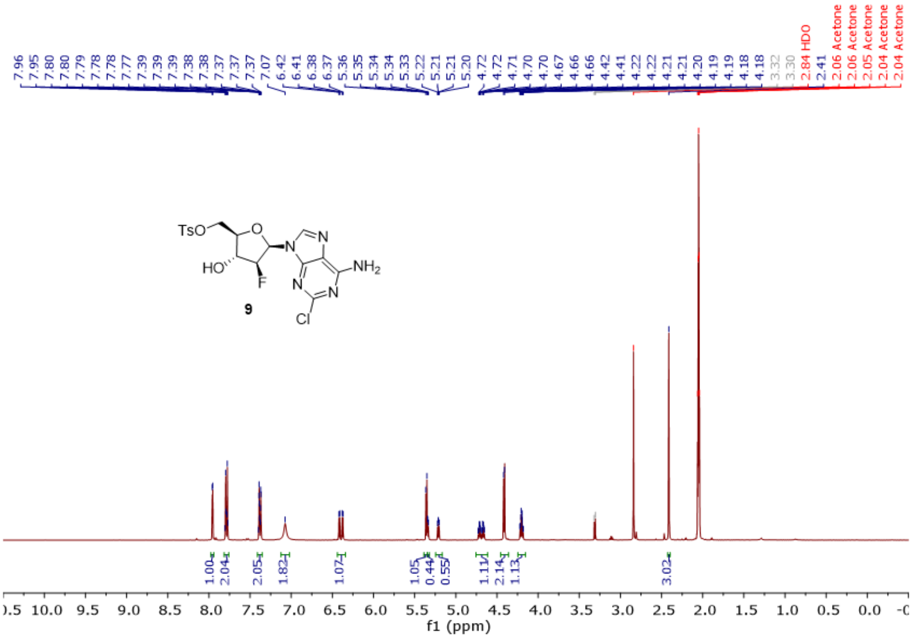

^{1}H-NMR (400 MHz, acetone-*d*6)

Compound **9**

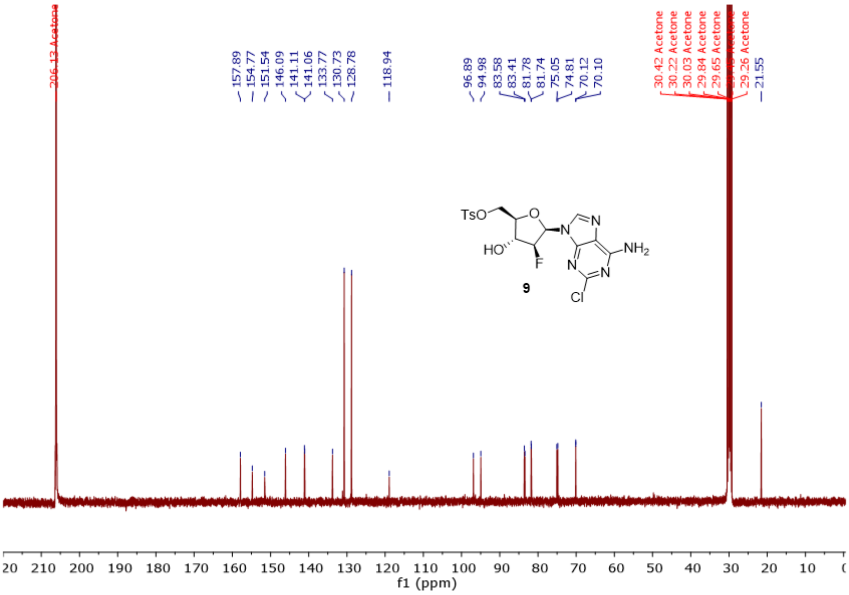

^{13}C-NMR (101 MHz, acetone-*d*6)

Compound **9**

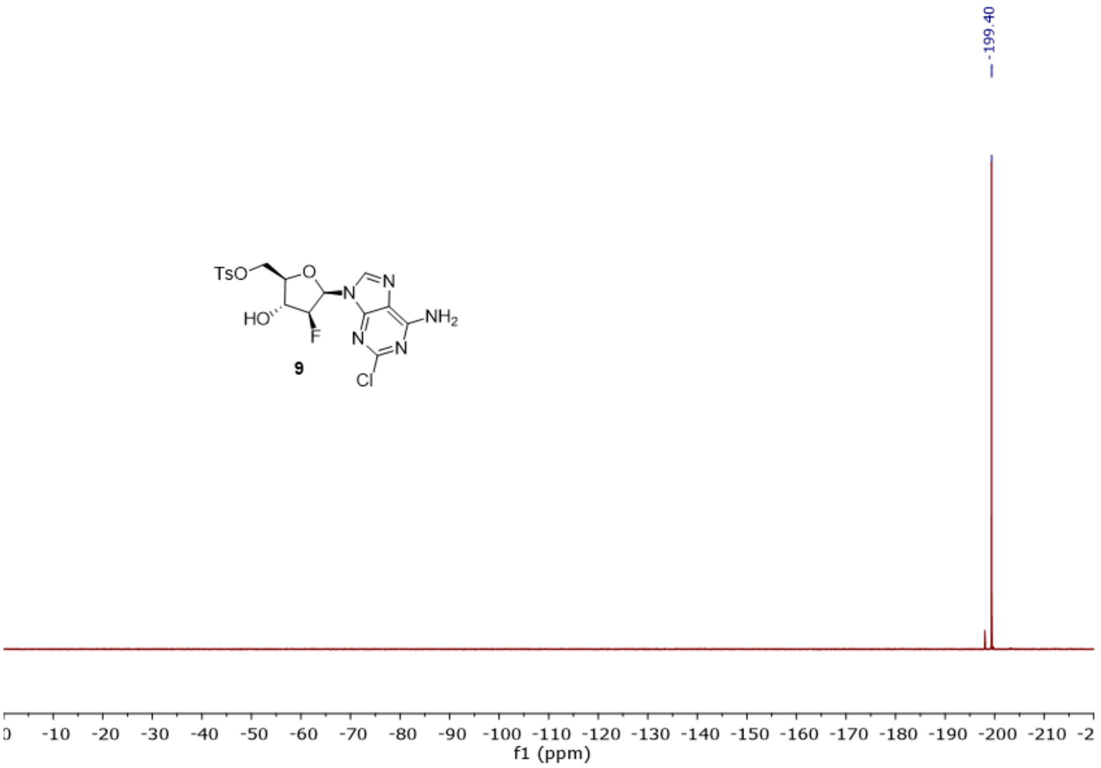

^{19}F-NMR (376 MHz, acetone-*d*6)

Compound **9**

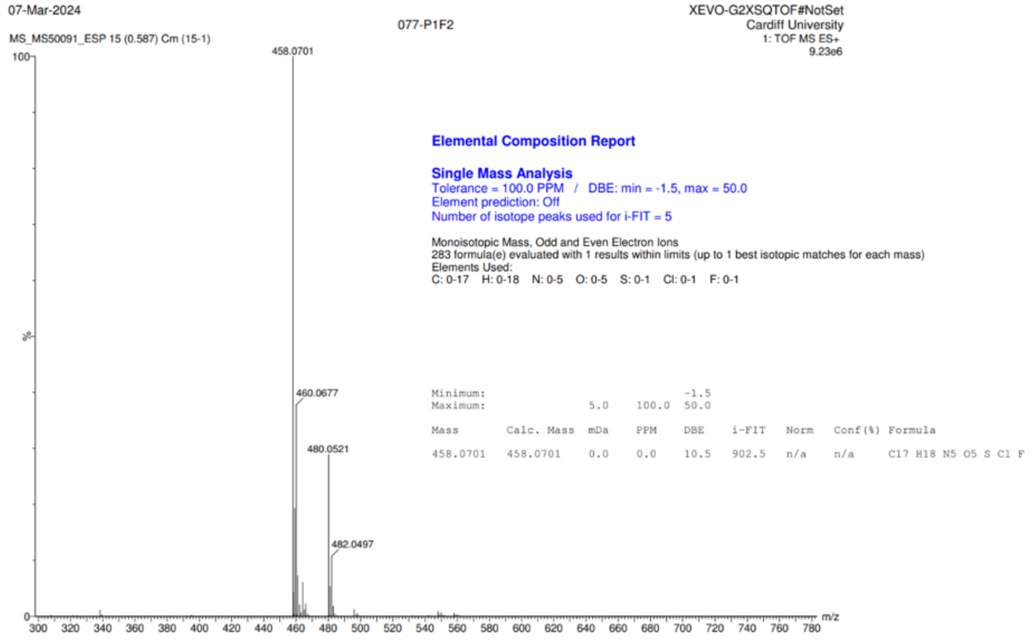

HRMS

Compound **9**

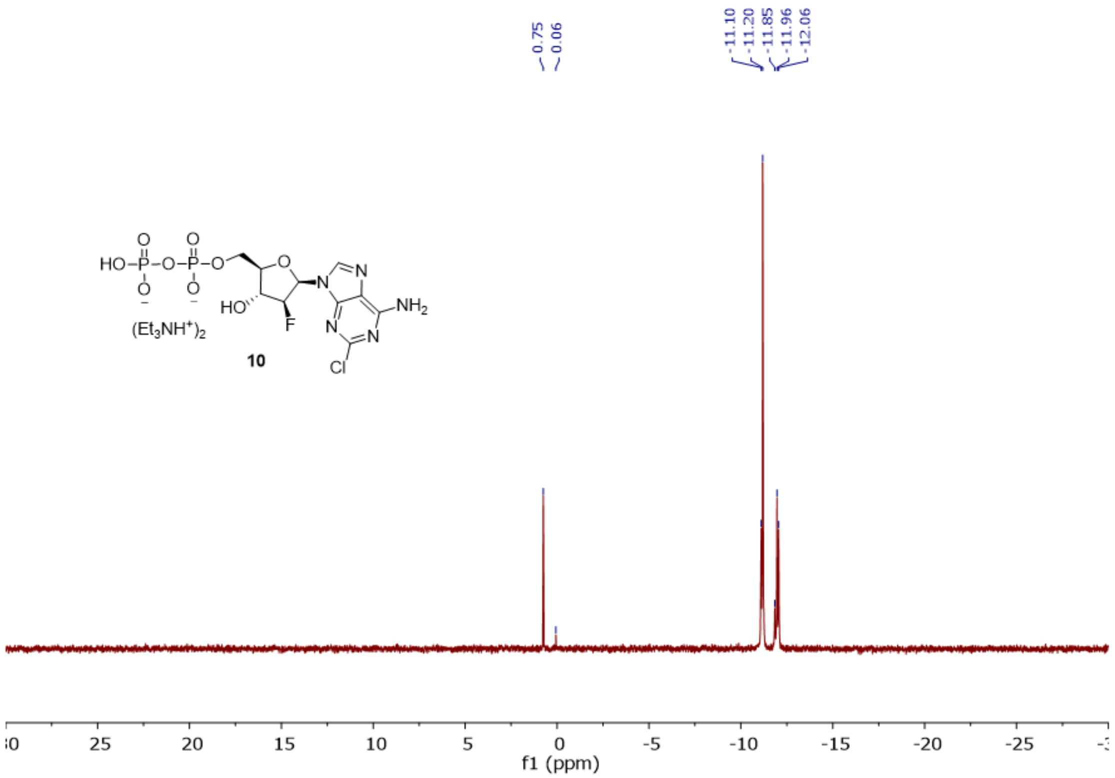

^{31}P-NMR (126 MHz, CD_3OD)

Compound **10**

^{1}H-, ^{31}P-, ^{19}F-, ^{13}C-NMR spectra, HRMS spectra and HPLC chromatograms of prodrugs 11a

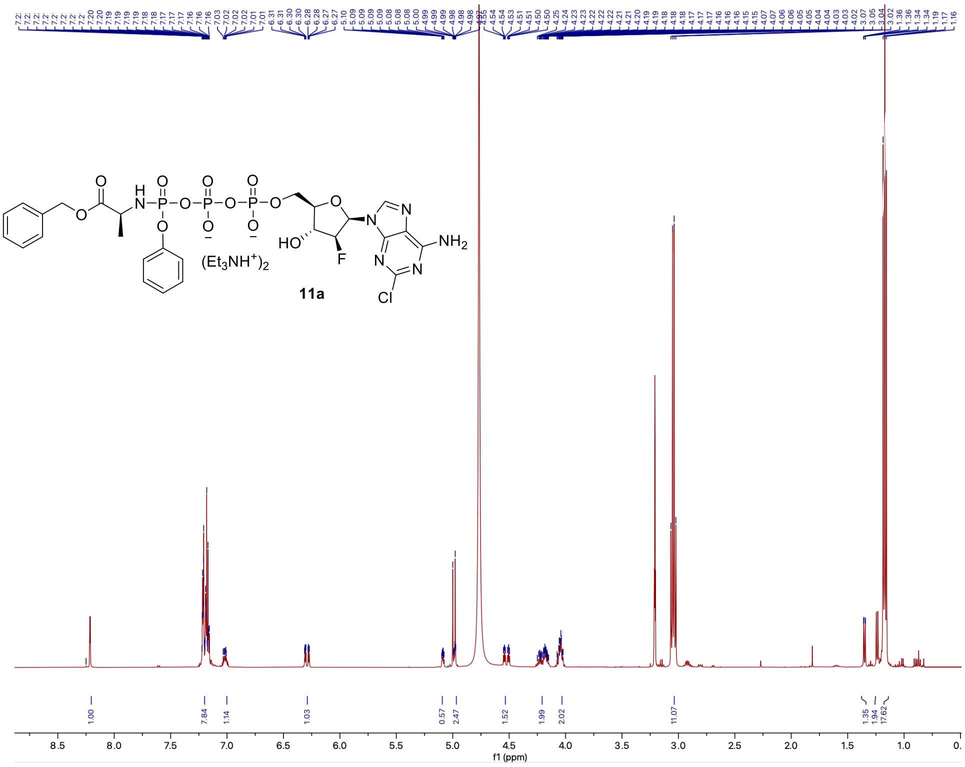

^{1}H-NMR (500 MHz, CD_3OD)
Prodrug **11a**

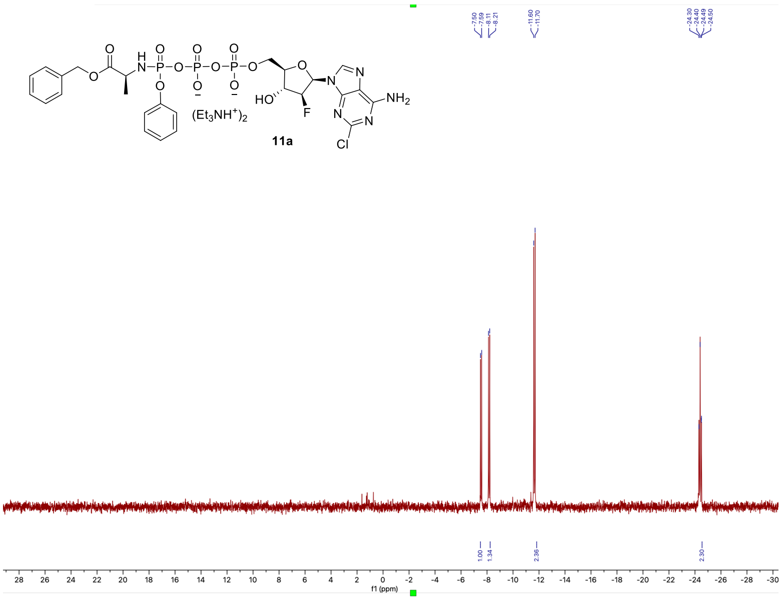

^{31}P-NMR (202 MHz, CD_3OD)

Prodrug **11a**

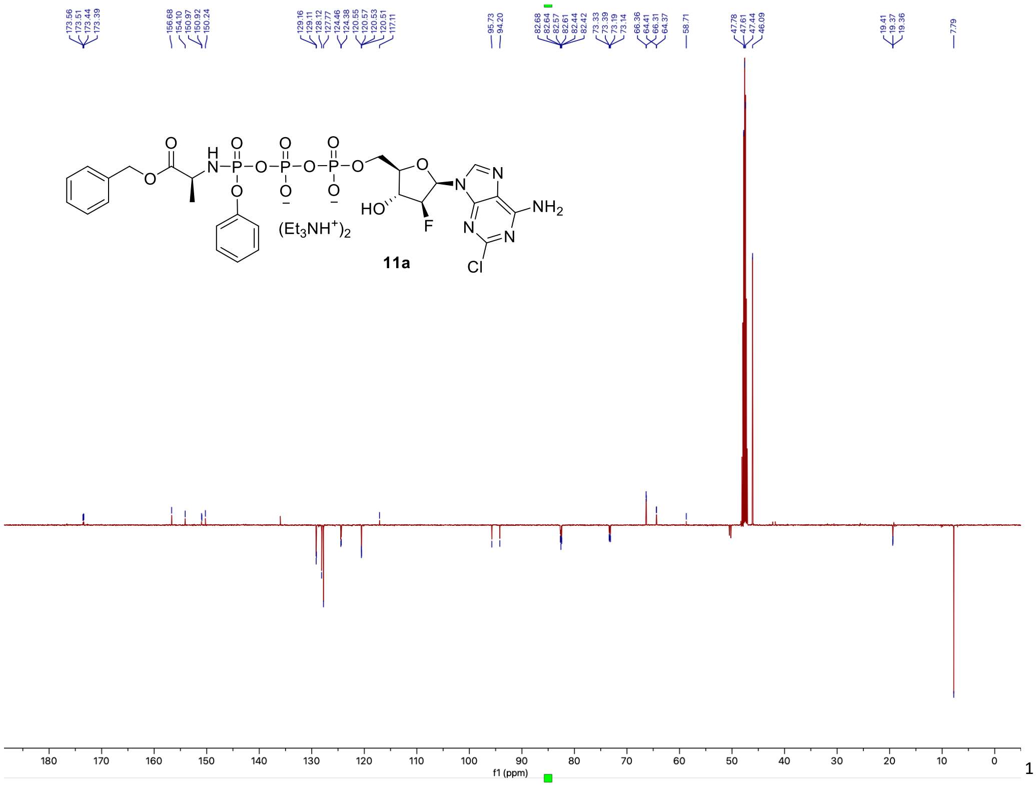

^{13}C-NMR (PENDANT) (126 MHz, CD_3OD)

Prodrug **11a**

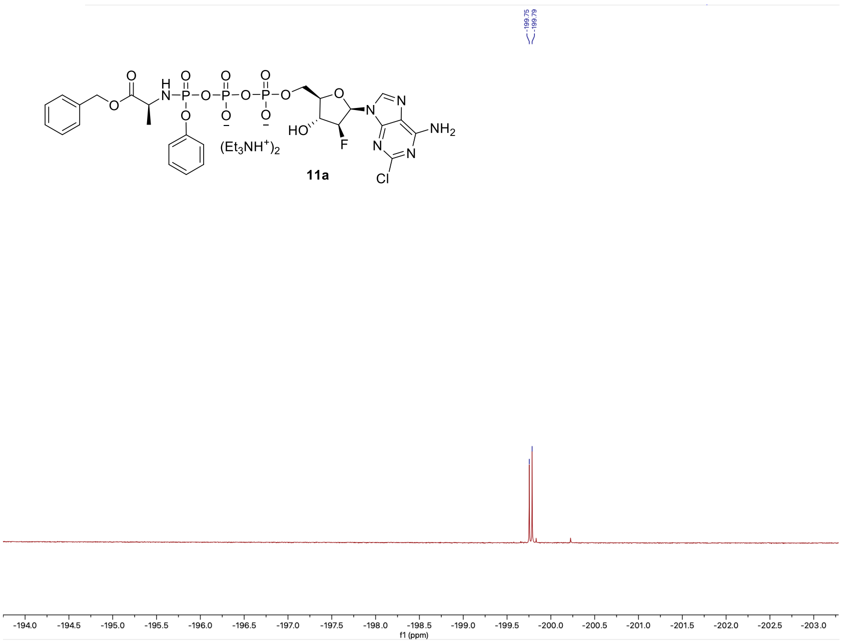

^{19}F-NMR (471 MHz, CD_3OD)

Prodrug **11a**

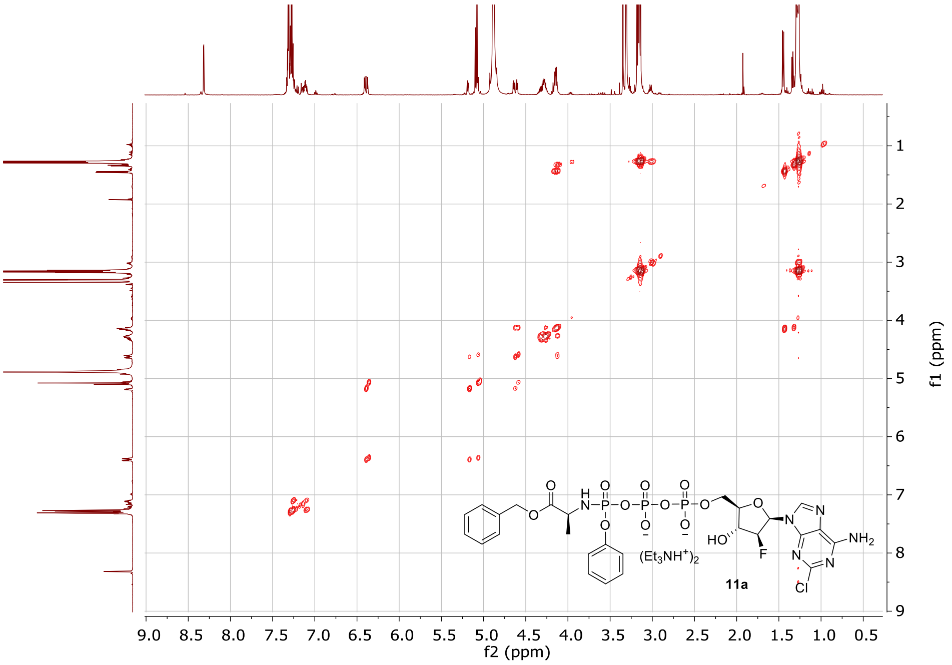

COSY NMR (500 MHz, CD_3OD)

Prodrug **11a**

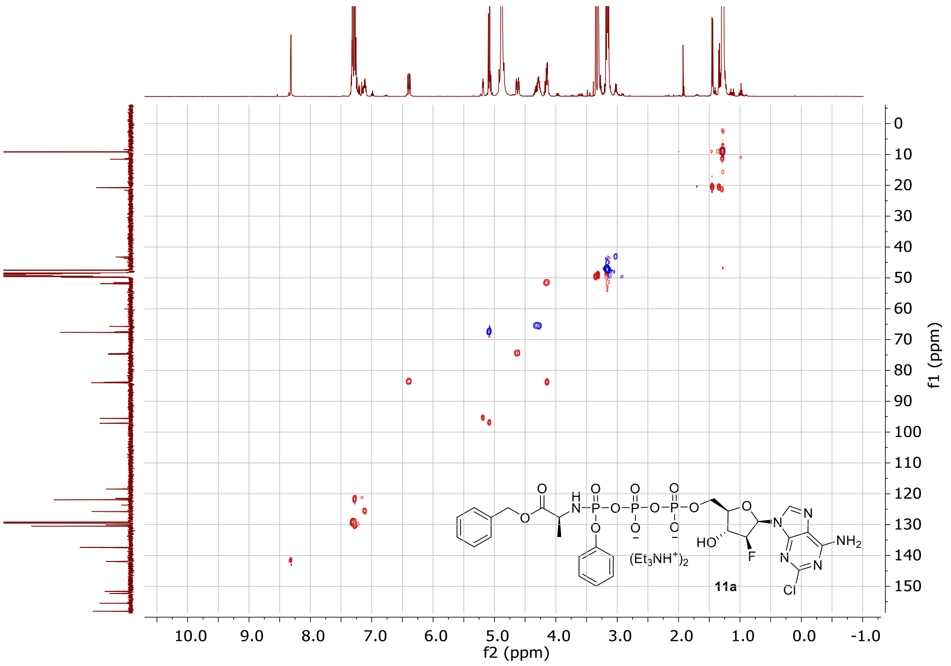

HSQC NMR (500 MHz, CD_3OD)

Prodrug **11a**

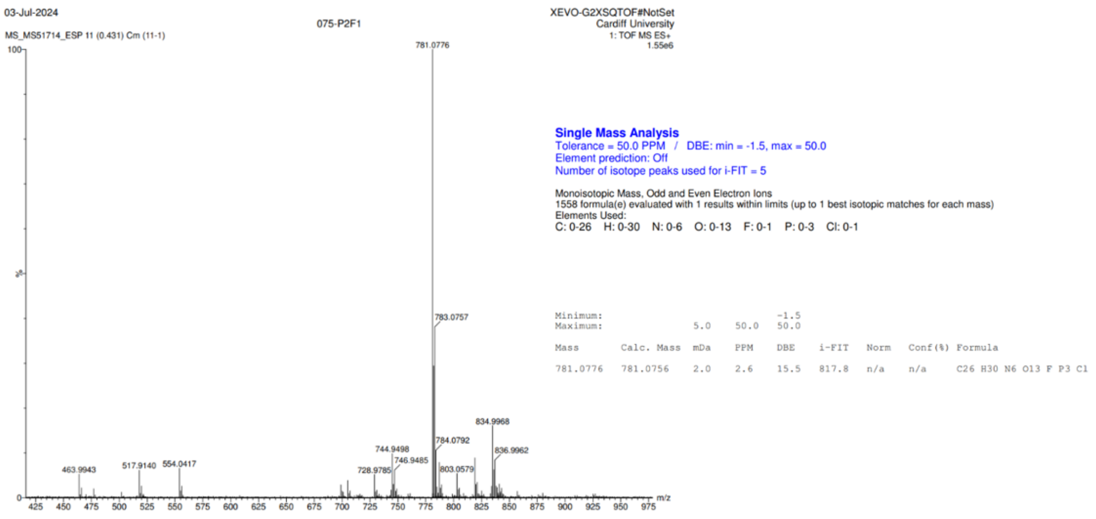

HRMS
Prodrug **11a**

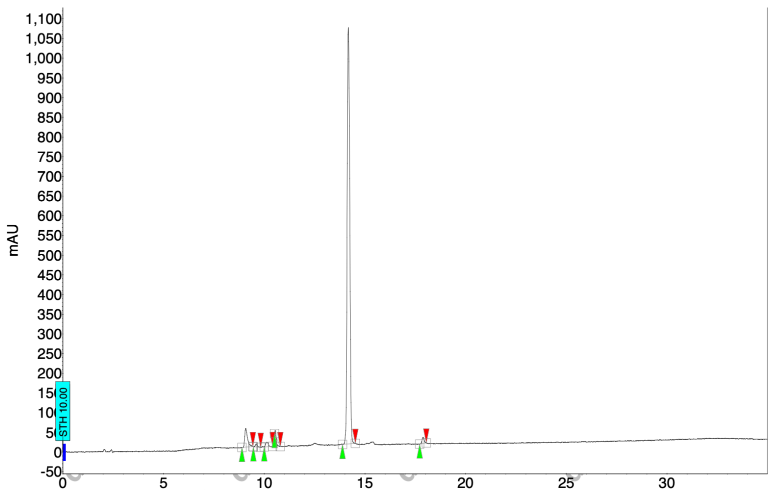

Peak results :

Index	Name	Time [Min]	Quantity [% Area]	Height [mAU]	Area [mAU.Min]	Area % [%]
2	UNKNOWN	9.08	4.44	47.6	7.6	4.439
3	UNKNOWN	9.60	0.54	8.0	0.9	0.536
4	UNKNOWN	10.12	0.97	11.3	1.7	0.966
6	UNKNOWN	10.64	0.60	13.3	1.0	0.601
1	UNKNOWN	14.17	92.35	1057.2	159.0	92.354
5	UNKNOWN	17.88	1.10	15.2	1.9	1.105
Total			100.00	1152.7	172.2	100.000

HPLC
Prodrug **11a**

^{1}H-, ^{19}F- and ^{13}C-NMR spectra and MS spectra of intermediates 12-16.

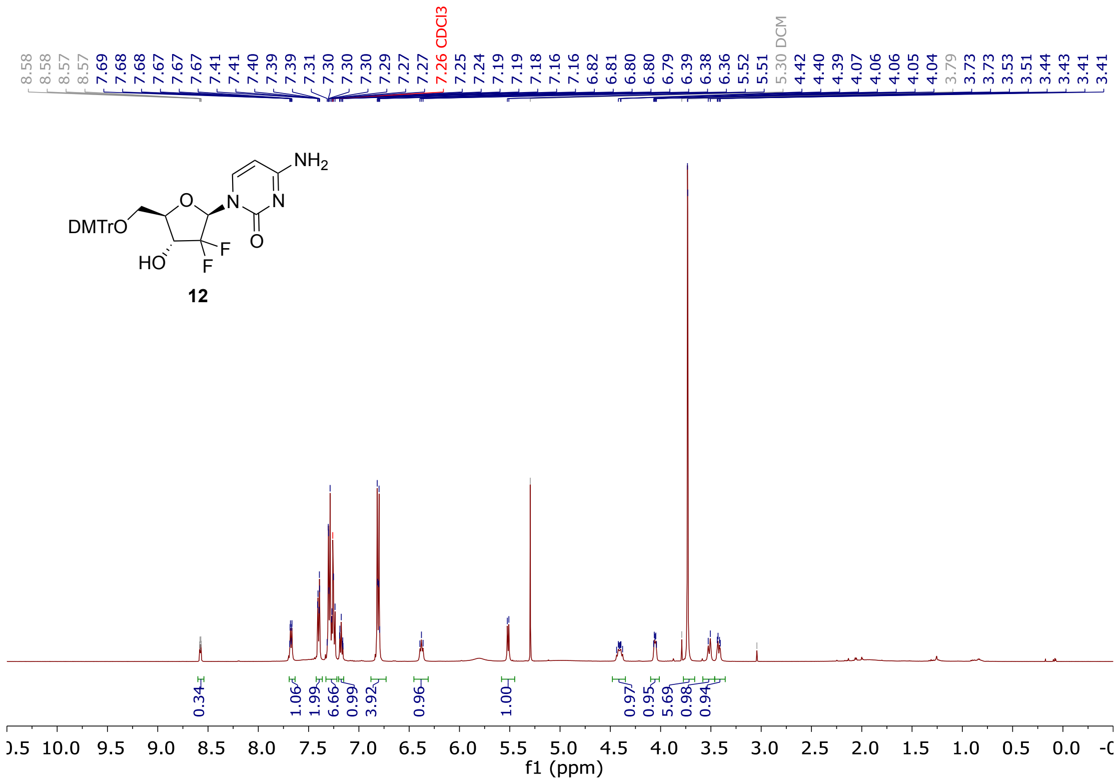

^{1}H-NMR (500 MHz, $CDCl_3$)

Compound **12**

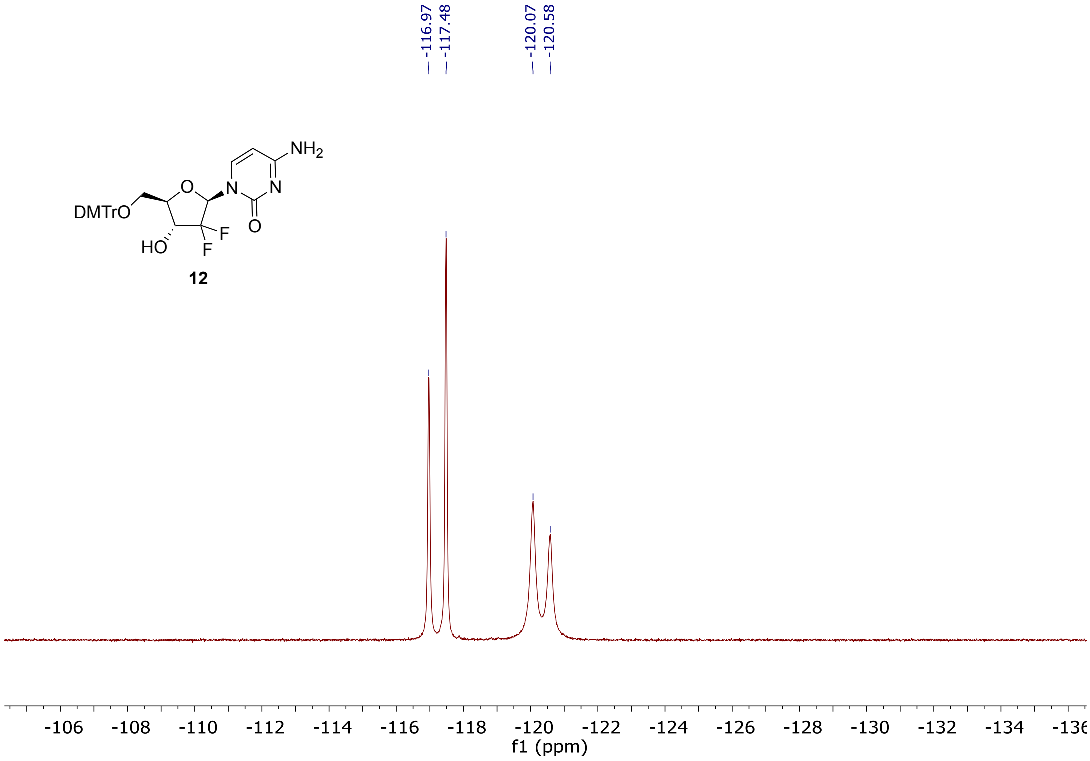

^{19}F-NMR (471 MHz, $CDCl_3$)

Compound **12**

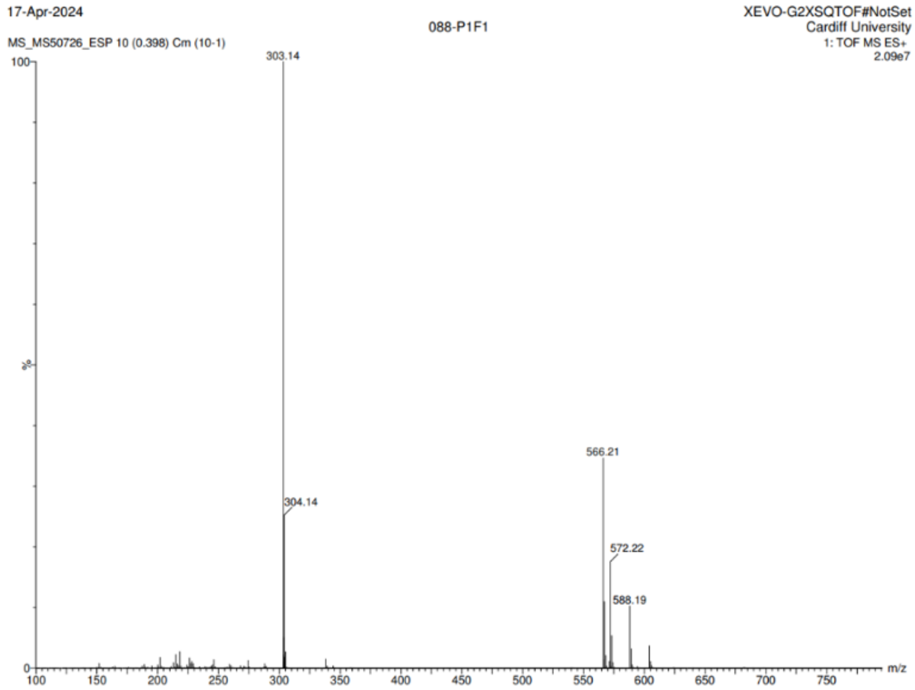

LRMS

Compound **12**

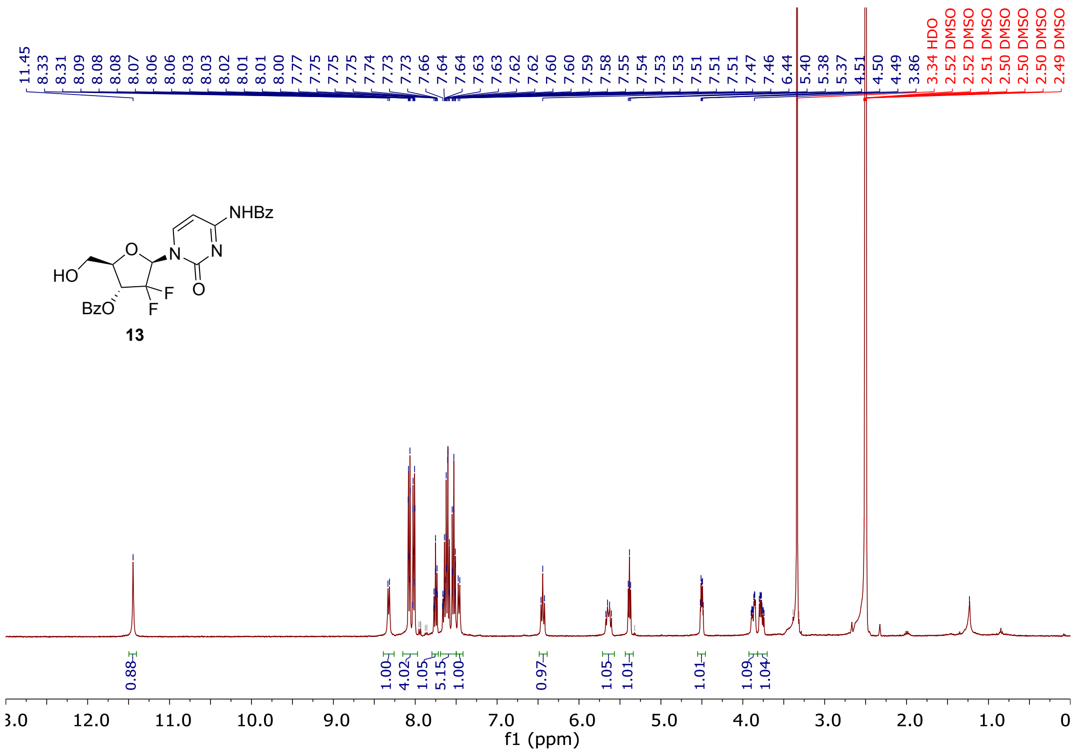

^{1}H-NMR (400 MHz, DMSO-*d6*)

Compound **13**

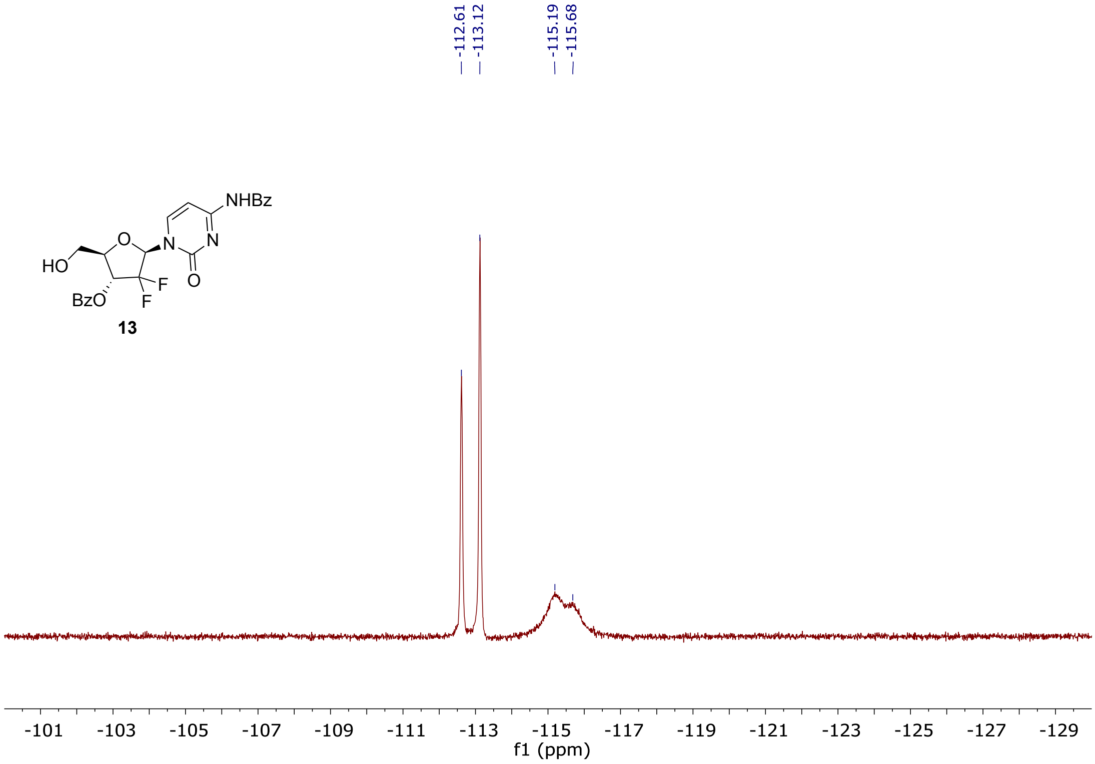

^{19}F-NMR (471 MHz, DMSO-*d6*)
Compound **13**

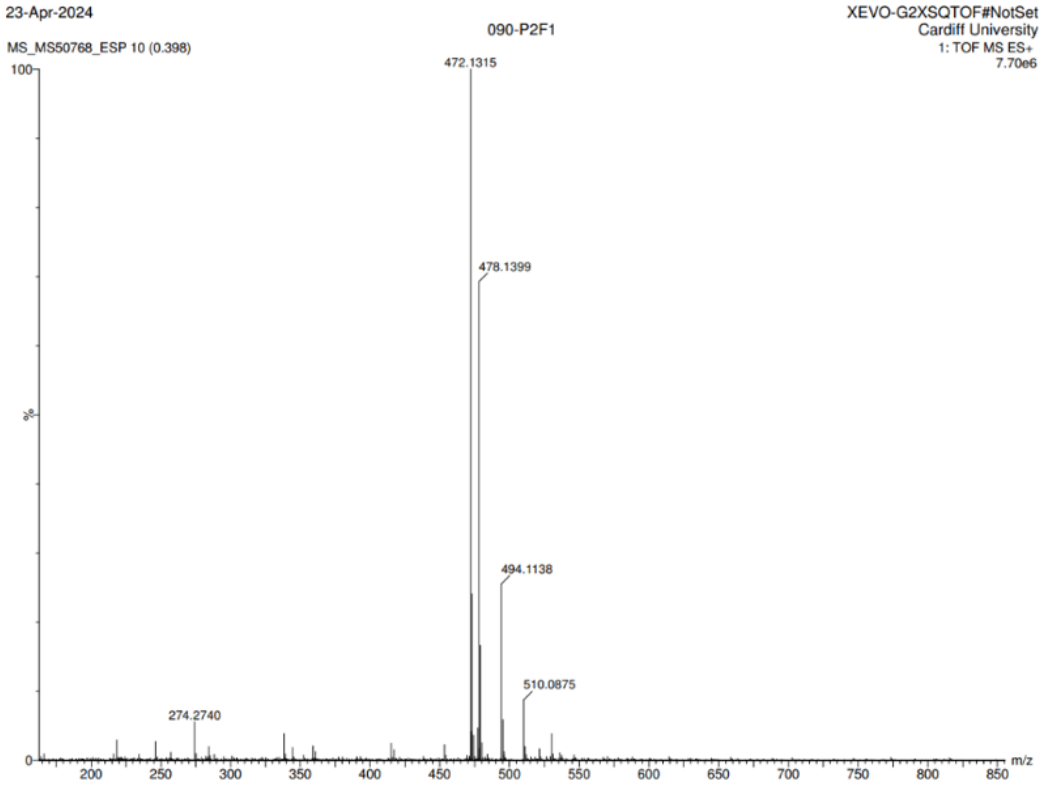

LRMS

Compound **13**

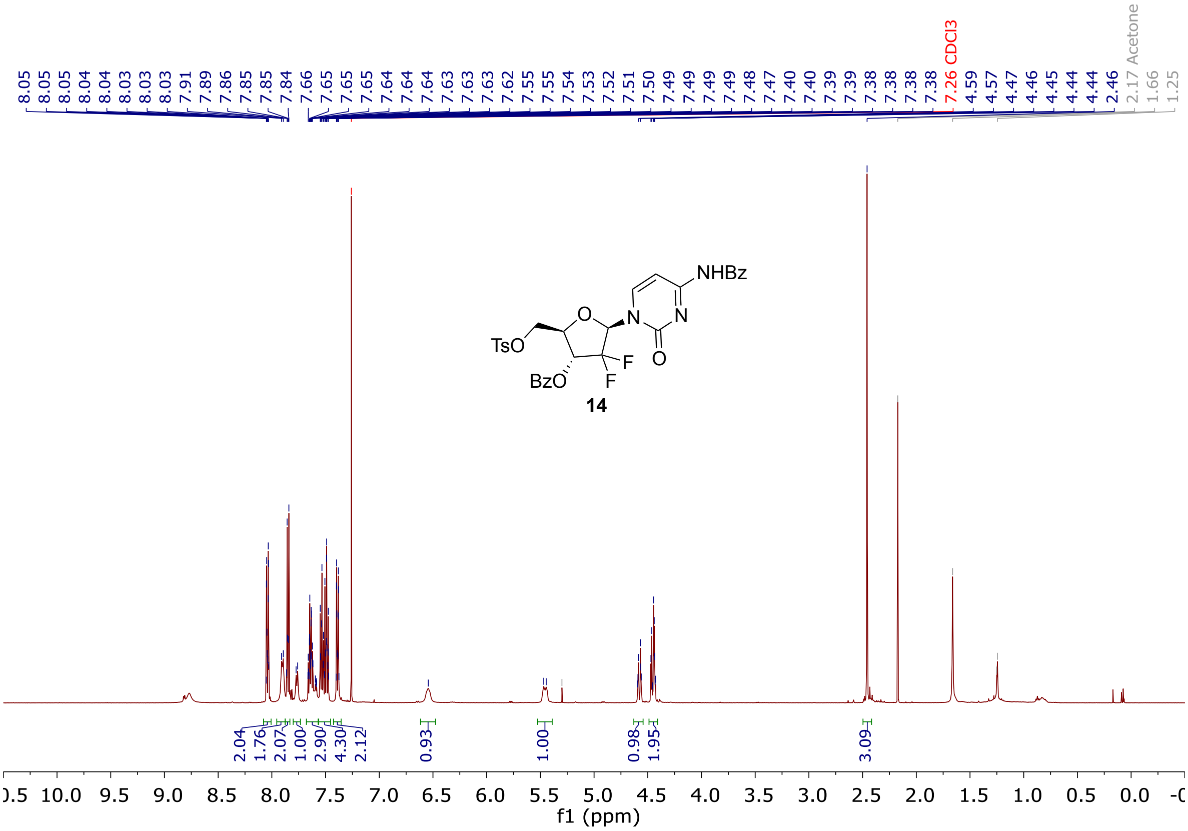

^{1}H-NMR (500 MHz, $CDCl_3$)

Compound **14**

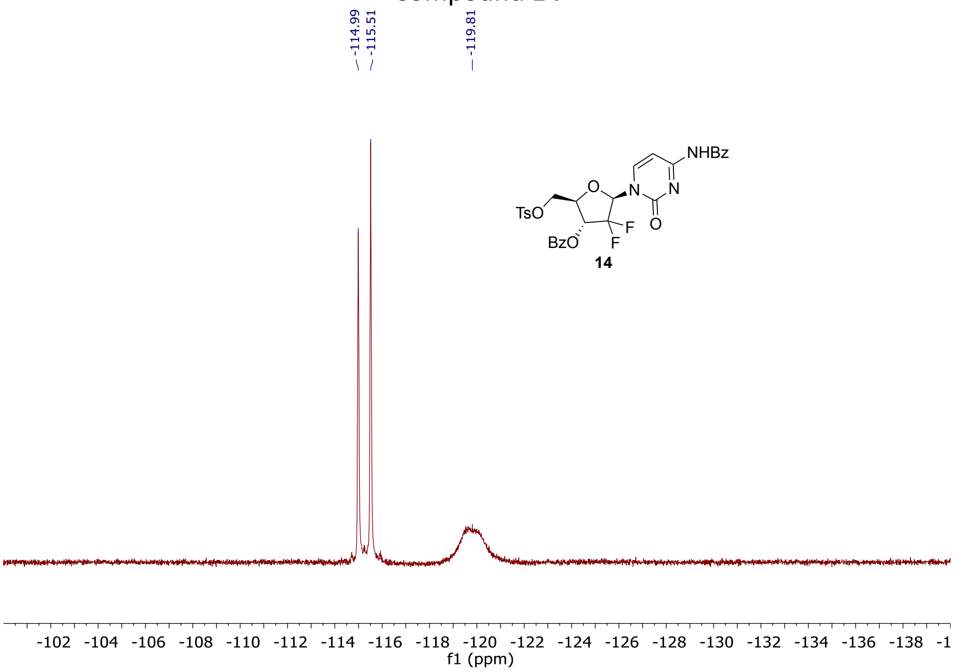

^{19}F-NMR (471 MHz, $CDCl_3$)

Compound **14**

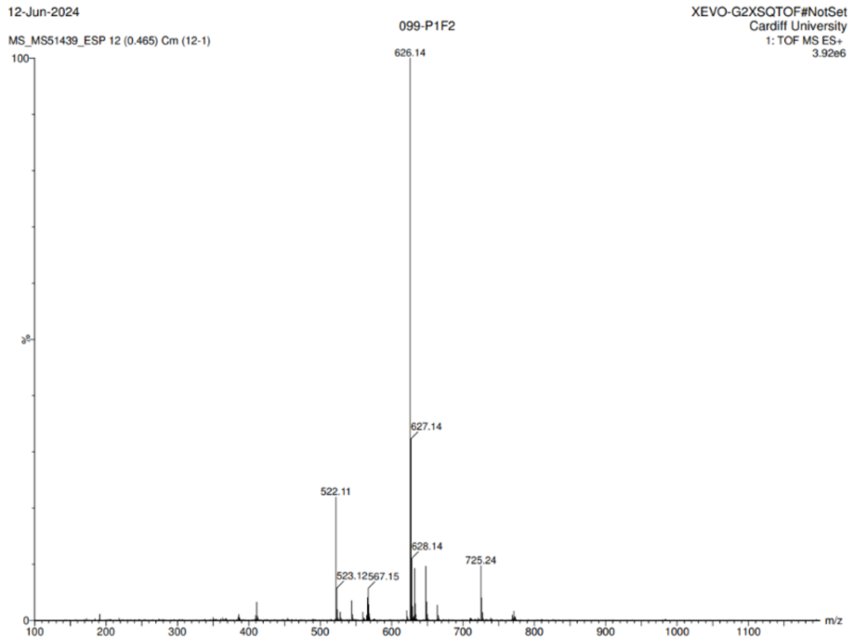

LRMS

Compound **14**

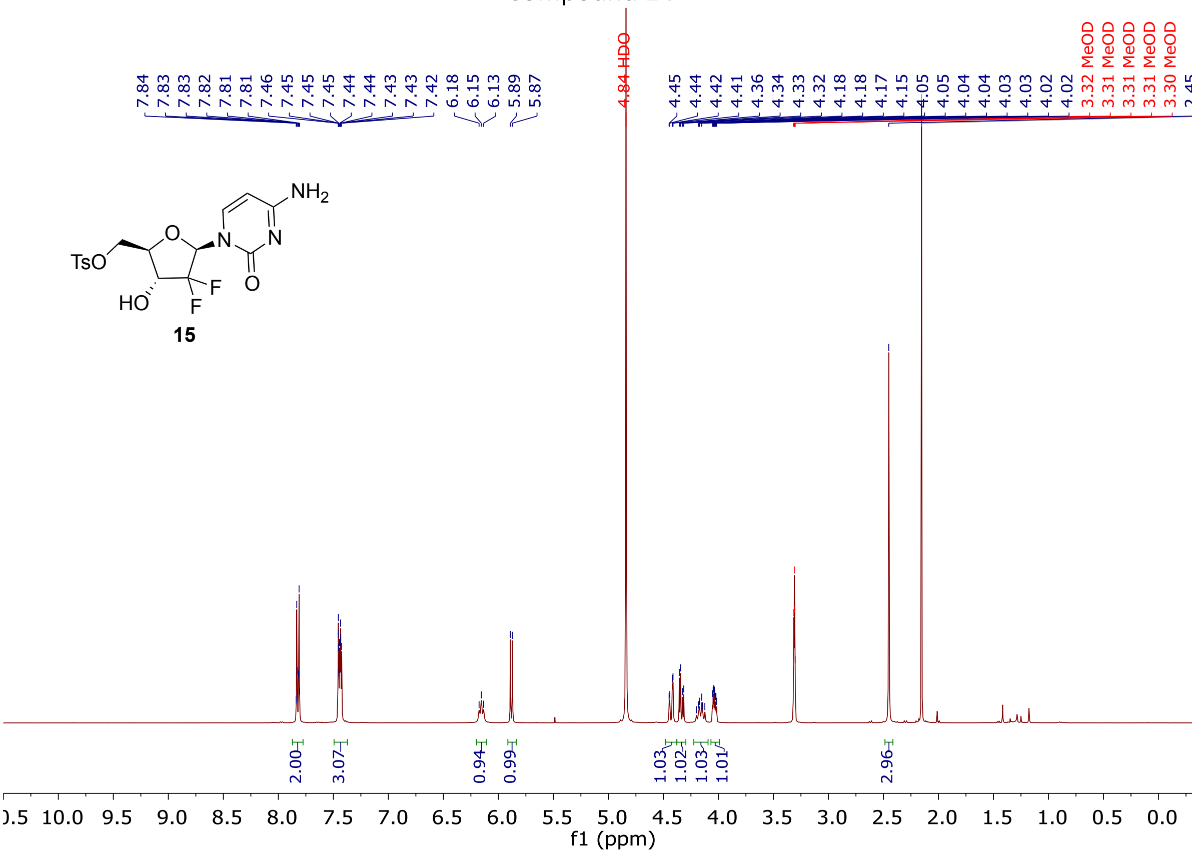

^{1}H-NMR (400 MHz, MeOD)

Compound **15**

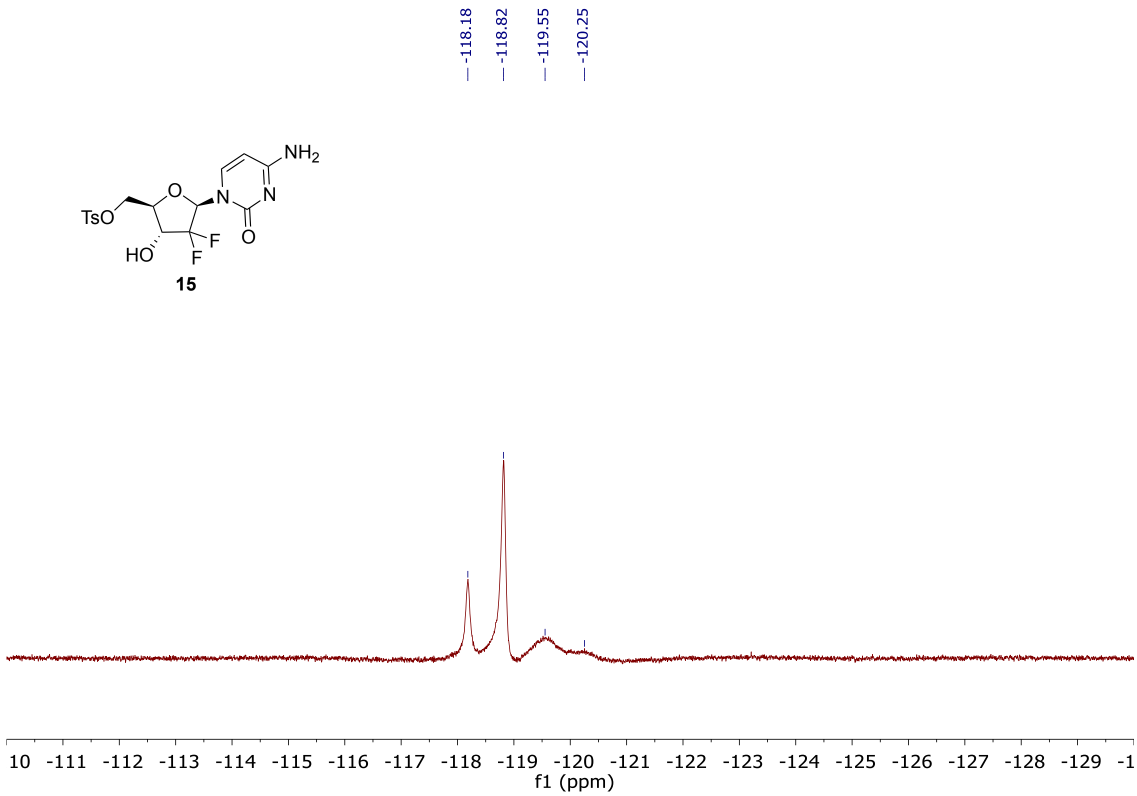

^{19}F-NMR (376 MHz, CD_3OD)

Compound **15**

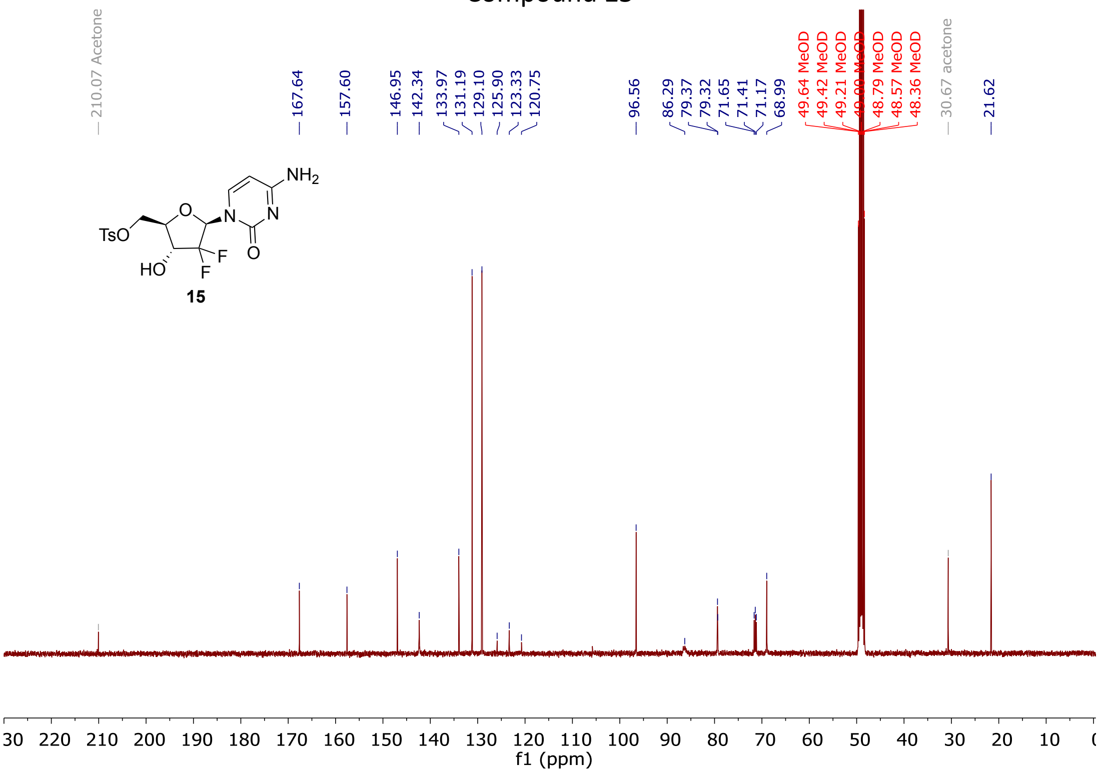

^{13}C-NMR (101 MHz, CD_3OD)

Compound **15**

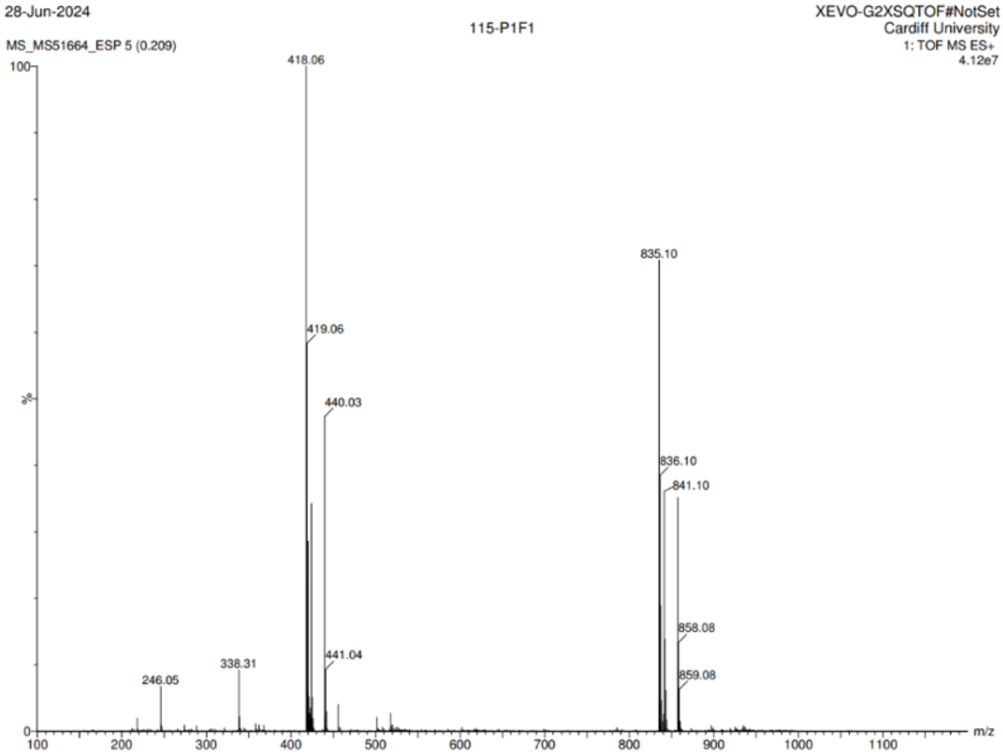

LRMS

Compound **15**

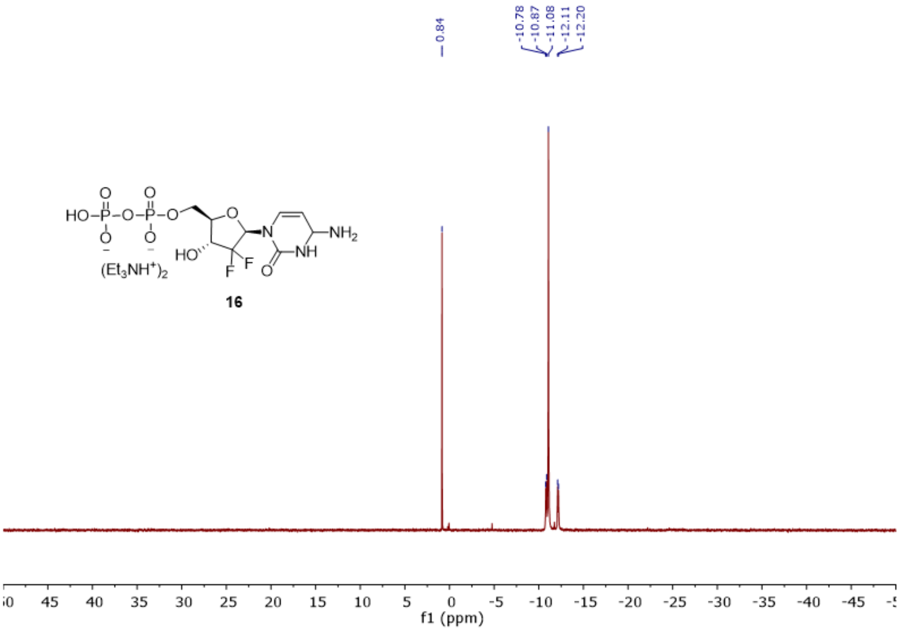

^{31}P-NMR (202 MHz, CD_3OD)

Compound **16**

^{1}H-, ^{31}P-, ^{19}F, ^{13}C-NMR spectra, HRMS spectra and HPLC chromatograms of prodrugs 17a

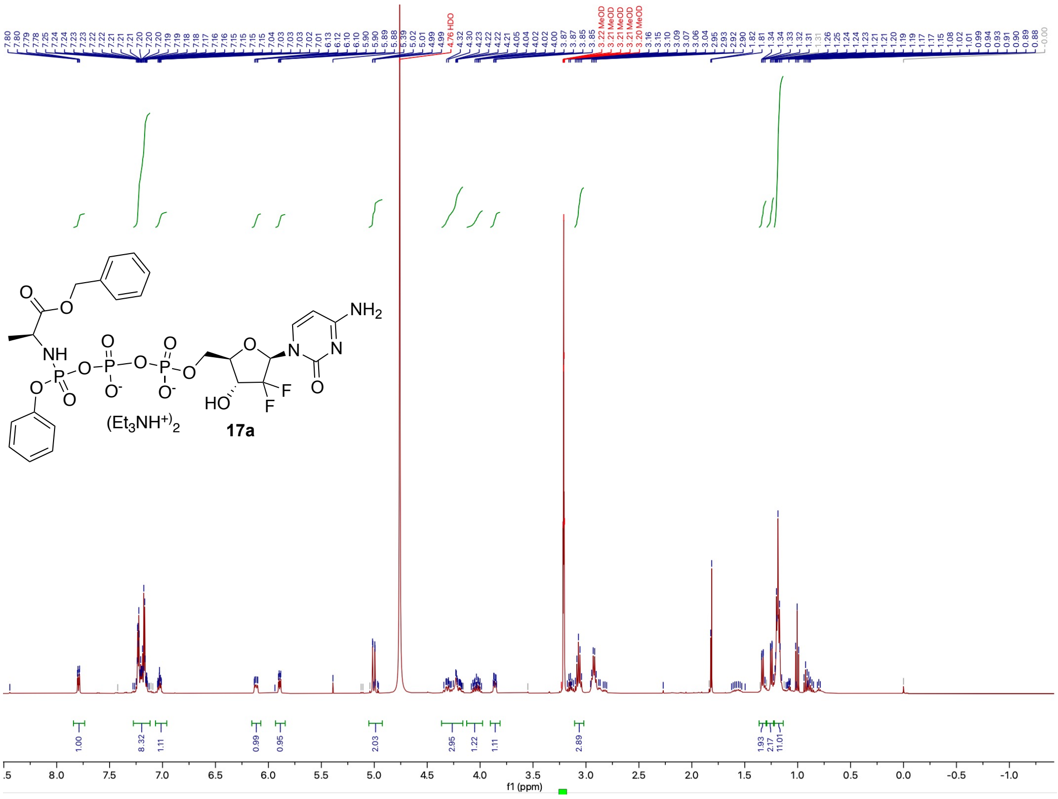

^{1}H-NMR (500 MHz, CD_3OD)
Prodrug **17a**

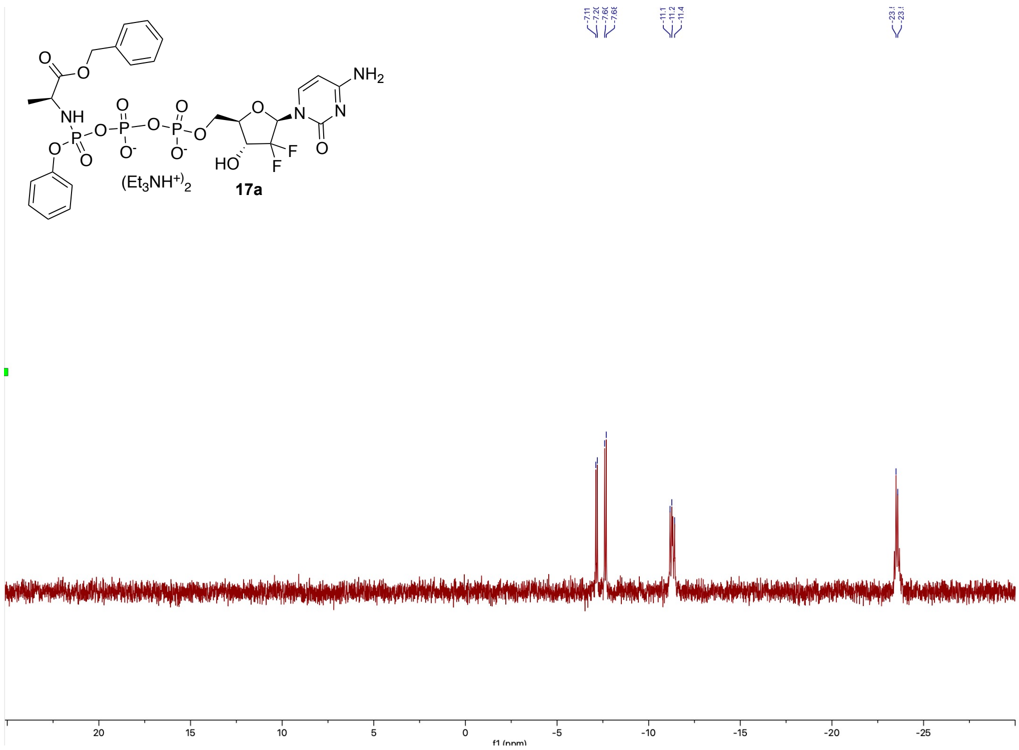

^{31}P-NMR (202 MHz, CD_3OD)
Prodrug **17a**

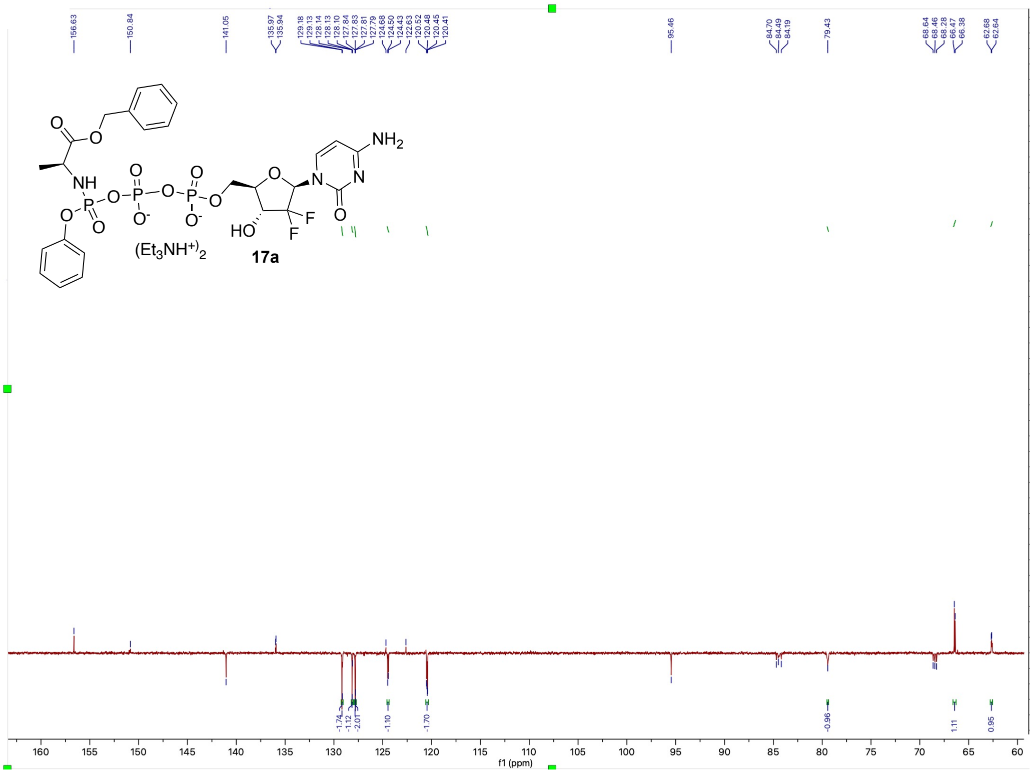

^{13}C-NMR (PENDANT) (126 MHz, CD_3OD)
Prodrug **17a**

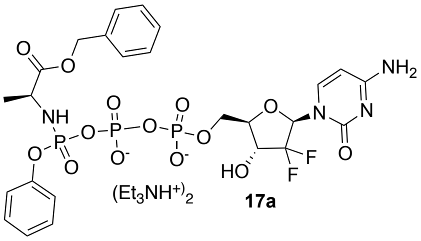

17a

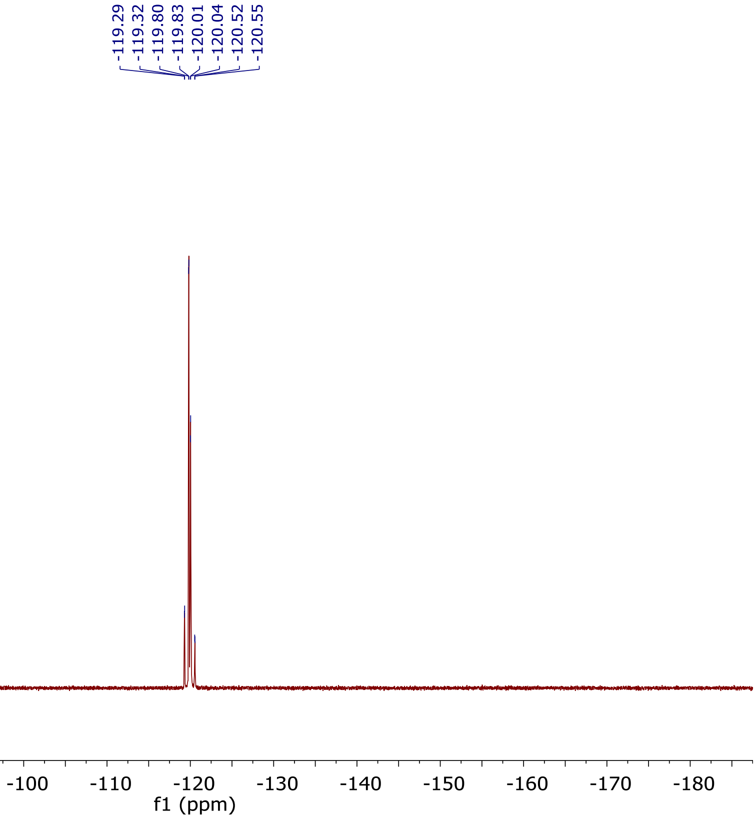

^{19}F-NMR (471 MHz, CD_3OD)

Prodrug **17a**

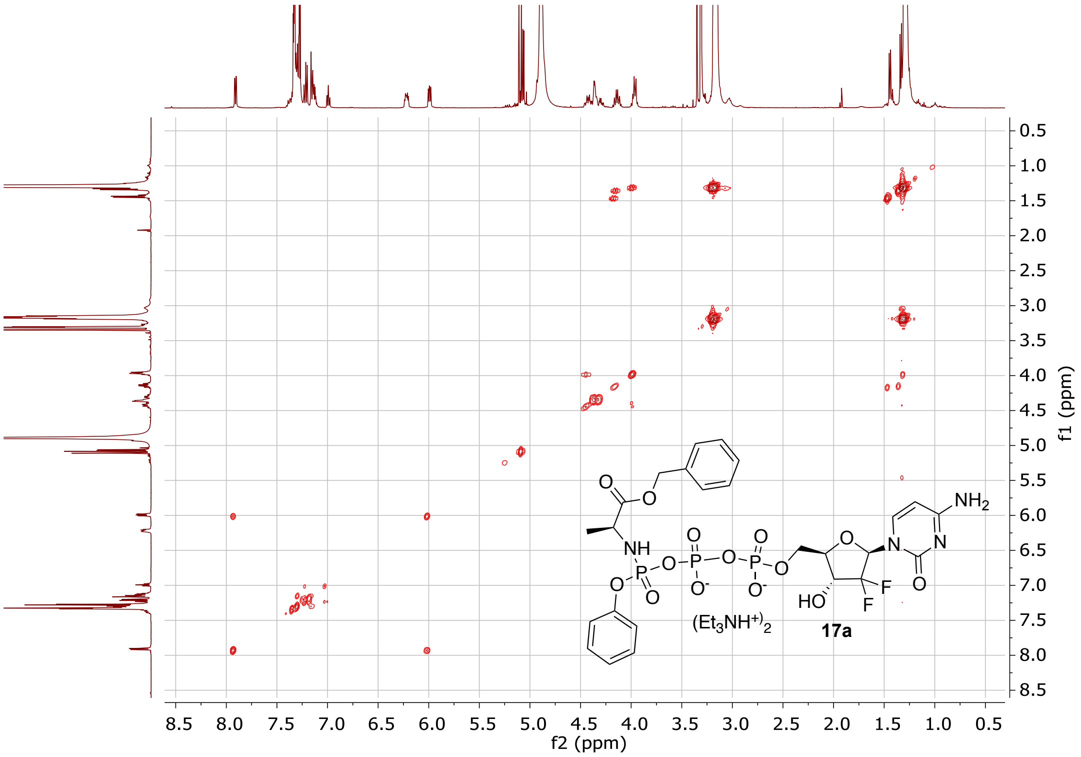

COSY NMR (500 MHz, CD_3OD)
Compound **17a**

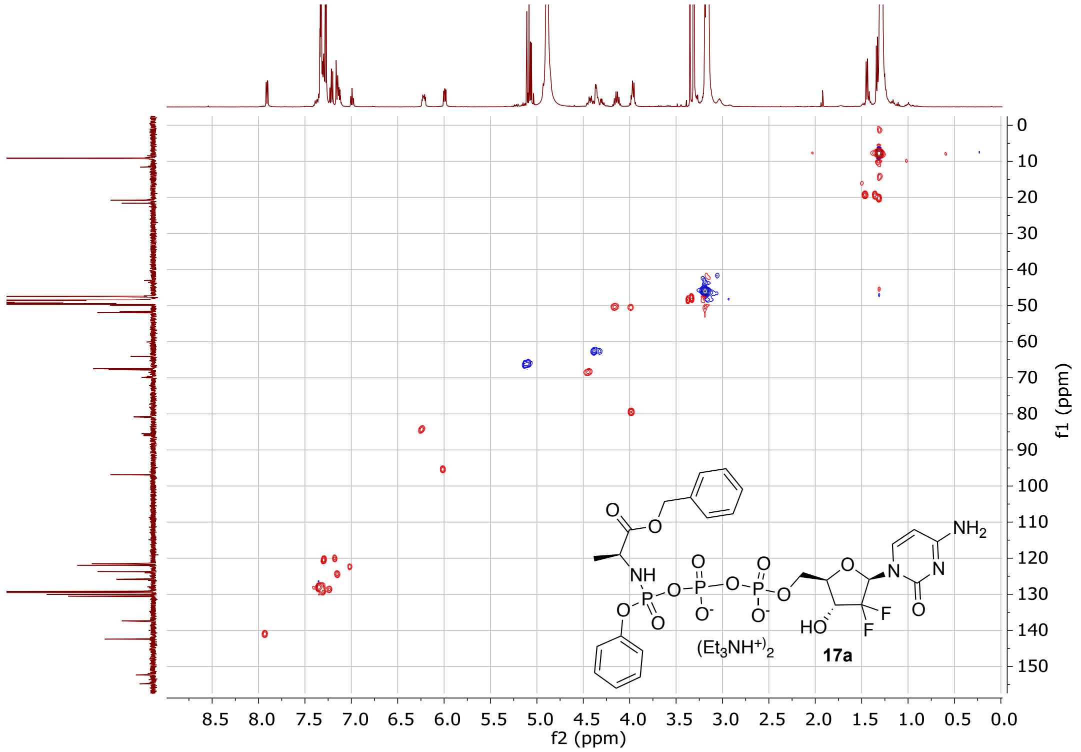

HSQC NMR (500 MHz, CD_3OD)
Compound **17a**

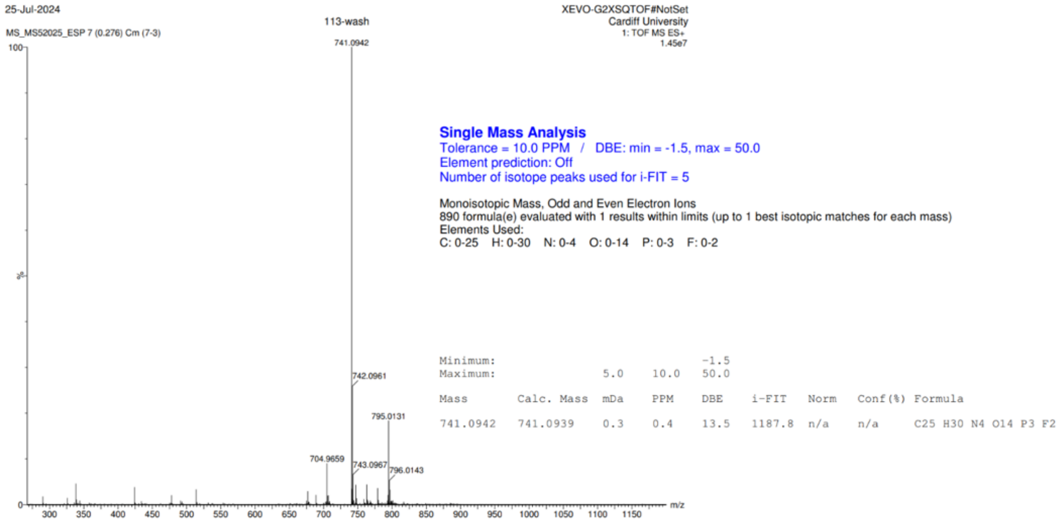

HRMS

Prodrug **17a**

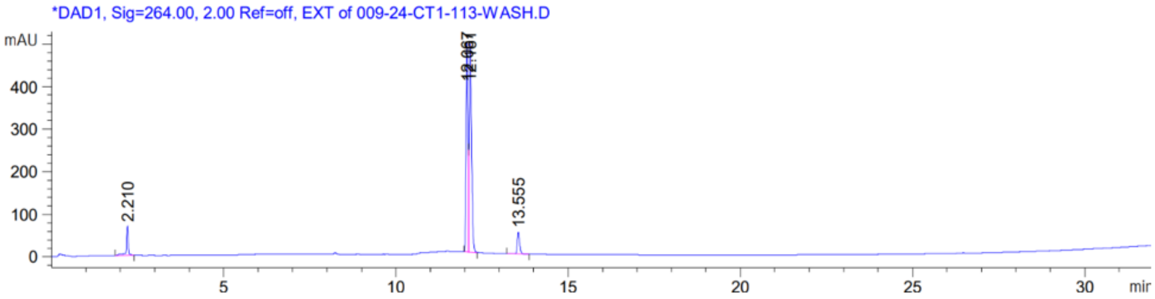

Signal 3: DAD1, Sig=264.00, 2.00 Ref=off, EXT of 009-24-CT1-113-WASH.D
Signal has been modified after loading from rawdata file!

Peak #	RetTime [min]	Type	Width [min]	Area [mAU*s]	Height [mAU]	Area %
1	2.210	VB R	0.0556	268.49652	67.99933	5.6020
2	12.067	BV	0.0620	1952.02734	494.80725	40.7276
3	12.161	VB	0.0692	2323.18213	490.60886	48.4715
4	13.555	VB R	0.0723	249.18040	51.60889	5.1990
Totals :				4792.88640	1105.02433	

HPLC

Prodrug **17a**

References:

1. Aye, Y.; Stubbe, J., Clofarabine 5′-di and -triphosphates inhibit human ribonucleotide reductase by altering the quaternary structure of its large subunit. *PNAS* **2011,** *108*, 9815-9820.
2. Kaczmarek, R.; Radzikowska, E.; Baraniak, J., Efficient Synthesis of Gemcitabine 5′-O-Triphosphate Using Gemcitabine 5′-O-Phosphoramidate as an Intermediate. *Synlett* **2014,** *25*, 1851-1854.
3. Labroli, M. A.; Dwyer, M. P.; Shen, R.; Popovici-Muller, J.; Pu, Q.; Wyss, D.; McCoy, M.; Barrett, D.; Davis, N.; Seghezzi, W.; Shanahan, F.; Taricani, L.; Beaumont, M.; Malinao, M.-C.; Parry, D.; Guzi, T. J., The identification of novel 5′-amino gemcitabine analogs as potent RRM1 inhibitors. *Bioorg. Med. Chem.* **2014,** *22*, 2303-2310.
4. Yang, S.; Luo, D.; Li, N.; Li, C.; Tang, S.; Huang, Z., New Mechanism of Gemcitabine and Its Phosphates: DNA Polymerization Disruption via 3'-5' Exonuclease Inhibition. *Biochemistry* **2020,** *59*, 4344-4352.
